# Interindividual Variation Refuses to Go Away: A Bayesian Computer Model of Language Change in Communicative Networks

**DOI:** 10.3389/fpsyg.2021.626118

**Published:** 2021-06-21

**Authors:** Mathilde Josserand, Marc Allassonnière-Tang, François Pellegrino, Dan Dediu

**Affiliations:** Laboratoire Dynamique Du Langage UMR 5596, Université Lumière Lyon 2, Lyon, France

**Keywords:** language evolution, iterated learning, interindividual variation, Bayesian agents, communicative networks

## Abstract

Treating the speech communities as homogeneous entities is not an accurate representation of reality, as it misses some of the complexities of linguistic interactions. Inter-individual variation and multiple types of biases are ubiquitous in speech communities, regardless of their size. This variation is often neglected due to the assumption that “majority rules,” and that the emerging language of the community will override any such biases by forcing the individuals to overcome their own biases, or risk having their use of language being treated as “idiosyncratic” or outright “pathological.” In this paper, we use computer simulations of Bayesian linguistic agents embedded in communicative networks to investigate how biased individuals, representing a minority of the population, interact with the unbiased majority, how a shared language emerges, and the dynamics of these biases across time. We tested different network sizes (from very small to very large) and types (random, scale-free, and small-world), along with different strengths and types of bias (modeled through the Bayesian prior distribution of the agents and the mechanism used for generating utterances: either sampling from the posterior distribution [“sampler”] or picking the value with the maximum probability [“MAP”]). The results show that, while the biased agents, even when being in the minority, do adapt their language by going against their a priori preferences, they are far from being swamped by the majority, and instead the emergent shared language of the whole community is influenced by their bias.

## 1. Introduction

As highlighted in the presentation of the Research Topic, “[t]he question whether all languages are similarly complex is at the center of some of the most heated debates within linguistics.” This statement is based on the axiomatic assumptions that, once complexity is defined, it is both *measurable* for each language and *commensurable* between languages. Needless to say, the fact that heated debates have been flourishing for at least two decades suggests that these assumptions have led to multiple interpretations of how complexity should be defined and how it should be considered, and consequently that the complexity jigsaw puzzle has still to be solved. Several contributions to this Research Topic specifically address these aspects, e.g., Ehret et al. (2021) on the equal complexity aspect, or Ehret et al. (2021) and Joseph (2021) on measuring complexity, to name just a few. Another heated debate is about the existence of putative complexity trade-offs within each language (i.e., do phonological, morphological, and syntactic complexities interact and compensate or combine?), as primarily discussed in Easterday et al. (2021). From an epistemological standpoint, this strand of research pertains to the notion of *magnitude of complexity*, a term coined as early as the beginning of the twentieth century in linguistics (e.g., Zipf, 1965, p. 66).

Here we adopt a different perspective on linguistic complexity, namely the view that language is a *complex adaptive system*. This strand of research stemmed from the field of cybernetics after World War II and thrived in the 1970s. In his more recent work, Jakobson adopted this perspective, stating that “[l]ike any other social modeling system tending to maintain its dynamic equilibrium, language ostensively displays its self-regulating and self-steering properties” (Jakobson, 1973, p. 48). More recently, the fact that language exhibits properties, such as emergence, self-organization, etc., typically explaining the dynamics and structure of complex adaptive systems, was convincingly articulated by Beckner et al. (2009) in a seminal paper, and is further supported by many theoretical, simulation-based, and experimental studies (see e.g., the contributions in Mufwene et al., 2017, among others). From this perspective, the main question is not to determine whether language A is more or less complex than language B (or whether a difference between their, let's say, phonological complexity, is compensated by a difference in syntactic complexity in the opposite direction), but to understand the mechanisms that explain the observed variation, its extension, and its evolution. As pointed by Forker (this issue), variation is probably an important aspect influencing the course of linguistic evolution, and her contribution echoes what can also be referred to as degrees of freedom in a systemic approach. In our paper, we aim at better understanding how the existence of variation among speakers within a population (or linguistic community) may shape the language (as a social convention) and its evolutionary trajectory through time (in the sense of change in a cultural evolutionary system on the glossogenetic timescale and not during human evolution at the phylogenetic timescale; Fitch, 2008). Our approach adopts a multi-agent simulation paradigm and is thus a computer modeling contribution to this Research Topic, inscribed in a productive research tradition of simulation studies using simplified languages and simplified linguistic agents acting in a simplified (socio-linguistic) environment (see below for a state of the art and references). Specifically, we focus on language change in heterogeneous populations containing a proportion of agents that are intrinsically biased toward a variant of the language. Thus, we aim to use this agent-based approach to understand whether a small proportion of individuals with such a bias can influence the structure of the language of the whole population, whether the bias of some individuals can resist to the pressure of the majority, and what effect (if any) does the structure of the network have on the rate of convergence.

Despite being so often repeated, the fact that there are about 7,000 languages being used around the world (Hammarström et al., 2018) should still evoke awe and wonder. This diversity is not restricted to the “languages,” but instead pervades all levels below and above it: from the striking geographic skew of the distribution of languages and language families, and of the number of their speakers, to intra-linguistic dialectal and sociolinguistic variation, and to the myriad ways individuals differ in how they acquire, perceive, process, and produce language (Dediu et al., 2017; Hammarström et al., 2018). Despite centuries of inquiry, the reasons for this diversity and its patterning remain one of the greatest enigmas of the language sciences (Evans and Levinson, 2009). However, one of the main explanatory factors is the way changes in language, usually small, accumulate, and amplify across time in space, resulting in this astonishing diversity (Evans and Levinson, 2009; Levinson and Evans, 2010; Bowern and Evans, 2014; Dediu et al., 2017). There are currently many proposals that identify various factors shaping language change, ranging from those *internal* to language (Lass, 1997; Campbell, 1998; Bowern and Evans, 2014), to *demography* and *population movements* (Ostler, 2005; Hua et al., 2019), to *environmental and ecological* factors (Everett et al., 2016; Bentz et al., 2018), and even to the *biology and cognition* of the language users (Dediu et al., 2019; Wong et al., 2020). However, this enigma cannot be answered without fully embracing the complexity of language itself, “evolving” and “living” at the interface of biology, cognition, society, and culture (Levinson, 2006; Mufwene et al., 2017).

Here, we take a broad *cultural evolutionary* view of language change (Cavalli-Sforza and Feldman, 1981; Croft, 2008; Richerson and Boyd, 2008; Dediu et al., 2013) in which linguistic variation is first generated through innovation, and then it may spread (or not) through the linguistic community, due to the complex interplay between random factors (akin to drift in evolutionary biology) and various types of selective pressures (or biases). Even though predicting language change (and evolutionary change, in general) is notoriously hard (Stadler, 2016), the mechanisms underlying language change have been the object of intensive study in particular in sociolinguistics (Milroy and Gordon, 2008; Meyerhoff, 2015) and historical linguistics (Bowern and Evans, 2014), but also in phonetics and phonology (Ohala, 1989; Yu, 2013). Of special interest is the so-called “*actuation problem”* (Weinreich et al., 1968; Yu, 2013; Dediu and Moisik, 2019), which can be briefly stated as “[w]hy do changes in a structural feature take place in a particular language at a given time, but not in other languages with the same feature, or in the same language at other times?” (Weinreich et al., 1968, p. 102). Multiple answers have been proposed, building upon various mechanisms. In sociolinguistics (Labov, 2010; Yu, 2013), the spread (or not) of linguistic variants is linked to their different valuations and to the frequency of interactions between interlocutors. Other explanations are based on selective forces that favor the spread of variants that are “better” functionally in some way (e.g., by optimizing articulatory effort, enhancing perception, or being cognitively easier to process; Christiansen and Chater, 2008; Croft, 2008; Blythe and Croft, 2012; Culbertson et al., 2012; Dediu et al., 2017; Blasi et al., 2019) or through frequency-dependent processes (Pagel et al., 2019). The mechanism of neutral evolution (or drift) where randomness plays the main role (Kauhanen, 2017) has also been suggested. Far from being mutually exclusive, these explanations are probably present to various degrees in many cases of language change.

However, an essential factor that is sometimes neglected by such theories is that language users differ not only with respect to their socio-economic and political roles, but in myriad other ways (Dediu et al., 2017; Dediu and Moisik, 2019), and it has been suggested that focusing on this pool of inter-individual variation may help solve the long-standing actuation problem (Baker et al., 2011; Stevens and Harrington, 2014; Dediu and Moisik, 2019). Here, we are focusing on a specific aspect of actuation, namely on the spread of linguistic variants in a network of language users that have different capacities, constraints and preferences (which we generically term *biases*). While language users may diverge with regard to their biases, they are also embedded in a converging *communicative network* that structures their repeated linguistic interactions. Biases can be found as ubiquitous variation among normal individuals in the acquisition, perception, processing, and production of language (it is important to highlight here the *normal* dimension of variation, as opposed to the much more studied extremes of this variation usually regarded as pathological). This ranges from variation in the *anatomy of the speech organs* (such as the shape of the hard palate), producing subtle effects on the production of vowels (Dediu et al., 2019) and consonants (Moisik and Dediu, 2015; Dediu and Moisik, 2019), to the *learning of a second language* (Hanulíková et al., 2012; Xiang et al., 2015), to vocabulary size (Mainz et al., 2017), *speech rate* (Coupé et al., 2019), and to the *processing of pitch* in Heschl's gyrus, affecting the perception of linguistic tone even in native speakers of tone languages (Dediu and Ladd, 2007; Wong et al., 2020). For many more examples, see, among others, Stevens and Harrington (2014) and Dediu et al. (2017). As it is the case with the most complex phenotypes, this variation is due to complex interactions between genes, environment and culture (Deriziotis and Fisher, 2013; Dediu, 2015; Devanna et al., 2018), and is *pervasive, multivariate* and usually *very small*, in the sense that it doesn't significantly impede communication.

To make this more precise, an example—in some ways, extreme—might help: some languages and varieties, such as *Spanish, Italian, Scottish English*, and *Romanian*, use the alveolar trill /r/, but there is a small minority of native speakers that apparently cannot produce this sound. While this incapacity varies in degree and is resolved, in most cases, spontaneously or through speech therapy during childhood, it does persist into adulthood in a small percentage of the population otherwise not affected by other speech and language deficits. As it happens, one of the authors is such a case, as he cannot produce the alveolar trill used in his native language, and instead systematically replaces it with a slightly retroflex approximant/ɻ/; other such native speakers might use other substitutions (such as the voiced uvular trill /ʀ/ or the voiced uvular fricative /ʁ/). Importantly, this speech deficit is recognized by the native speakers and stigmatized (in fact, there is a particular mocking word for this idiosyncrasy), and is specifically targeted by teachers and speech therapists in children. Thus, using the concepts introduced above, this incapacity represents in some speakers a strong bias against the alveolar trill and, while its etiology is currently unclear and most probably diverse, it seems safe to assume that it is stable throughout the lifespan, costly to overcome for those that do, and negatively stigmatized by the speech community.

While the example above is of a strong bias present in only very few individuals, there are other types of inter-individual variation that result in (very) weak biases at the individual level that are, however, more widely shared within a group. For such cases, previous work has shown, using mathematical modeling, computer simulations, and experimental approaches, that variants induced by weak biases may be amplified by the repeated use and transmission of language under specific conditions (see Dediu et al., 2017; Janssen, 2018, for more comprehensive reviews). Early work under the Bayesian framework (Griffiths and Kalish, 2007; Kirby et al., 2007) has produced surprising results in the sense that, when considering simple transmission chains composed of one agent per generation, Bayesian samplers always converge on the prior, while maximum a posteriori (MAP) may amplify initially weak biases. Dediu (2008, 2009) shows that *ad-hoc* and Bayesian learning mechanisms behave differently in single-agent chains, homogeneous and heterogeneous two-agent chains, and complex populations, and that, in some cases, variants induced by weak biases are indeed expressed at the level of the community language. Navarro et al. (2018) show that mixing agents with different biases in the same transmission chain results in the expression of the variants induced by the stronger biases by the repeated transmission of language (“extremists win”), but in an indirect and non-transparent way. In their seminal work, Kirby et al. (2008) found that transmission chains composed of human participants also amplify individually weaker tendencies toward compositionality, findings that have been replicated, refined and contextualized since (see reviews in Tamariz and Kirby, 2015, 2016; Culbertson and Kirby, 2016). Focusing specifically on the anatomy of the vocal tract, Dediu et al. (2019) show, using a computer model of the vocal tract capable of learning to produce vowels (using artificial neural networks and genetic algorithms), that variation in the shape of the hard palate results in very weak effects on the production of the learned vowels. These weak effects are amplified by a classic iterated learning transmission chain to the level of observed intra-dialectal variation. In the same vein, Blasi et al. (2019) show, using a combination of approaches, that variation in bite due to food consistency between agricultural and hunter-gathering populations, results in tiny differences in the effort required to produce labiodental sounds (such as “f” and “v”). These differences in effort are presumably amplified to produce robust statistical differences in the frequency of these sounds between languages.

This amplification of weak biases thus raises a crucial question relevant to language evolution, change and diversity, and, more generally, to cultural evolution: under what conditions does this amplification take place (or doesn't)? But before we proceed, we need to clarify our terminology: on the one hand, such biases have *causes* (sociolinguistic, environmental, anatomical, etc.) and any given individual may or may not be affected, i.e., the bias may be *present* or *absent* (for discrete, binary biases, such as having a frenulum of the tongue) or have a certain *numeric value* (for continuous biases, such as the degree of overjet/overbite); when zooming out at the level of a linguistic community, we are then talking about the bias being present with a certain *frequency* (for discrete biases) or have a certain *distribution* (for continuous biases). On the other hand, such a variant, when present in an individual, may or may not be expressed in the individual's linguistic behavior (e.g., not being able to articulate the alveolar trill or a lower probability of producing labiodentals); at the level of the linguistic community, a variant can be expressed with a certain frequency or have a certain distribution, and it may (or may not) be further amplified by the repeated use and transmission of language. These concepts are parallel to those from medical genetics concerning the presence of a deleterious allele in an individual's genotype (say, a mutation in one of the opsin genes on the X chromosome), its phenotypic expression (as red/green abnormal color perception), and the population frequency of such deficiencies.

With these, the simplest question concerns, for a given bias, the minimum frequency of the biased individuals in the community (i.e., the individuals expressing the bias), so that its effects are expressed and amplified in the language of the whole community. To use our “extreme” alveolar trill example, we know that about 1% of non-trilling speakers (an estimate based on the available unsystematic data) is not enough to change the Romanian language away from the alveolar trill and toward, say, a “French-style” uvular fricative, but would 10, 25, 50% do? The complementary question is: for a given frequency, what is the minimum bias strength that would allow the variant to be expressed and amplified? And what is the time trajectory of the spread for a given strength and bias? On top of these questions, we must also not think of the speech community as a shapeless pool of speakers, each equally likely to speak to, and to learn from, any other speaker, which is completely unrealistic (Milroy and Gordon, 2008; Meyerhoff, 2015). Therefore, we focus here on speakers connected through communicative networks which structure the communicative exchanges, controlling thus the probability that any two speakers will interact. To the questions above concerning the bias strength and frequency, we thus add questions concerning the influence of the size of the network (the number of speakers in the community), of the structural properties of the network (random, small world, scale-free), and of the position that biased individuals have in the network (e.g., high vs. low centrality, bridging two subnetworks, etc.) on the spread of the bias.

The spread of innovation, behaviors and attitudes (among others) in social networks has received a lot of attention. Moreover, inter-individual variation seems to play an important role in these processes of network spread (Granovetter, 1978; Karsai et al., 2016). Language is not an exception, with studies ranging from “classic” sociolinguistics (Milroy and Gordon, 2008) to more recent network-centric (Ke et al., 2008; Fagyal et al., 2010; Abitbol et al., 2018). Language change has also been studied using real-world examples, such as the vowel chain shift in Ximu or the consonant convergence in Duoxu (Chirkova and Gong, 2014, 2019), and using experimental approaches (Raviv, 2020; Raviv et al., 2020) showing that we must consider the structure of the connectivity in linguistic communities. Social structure, and more specifically the average degree, the presence of shortcuts and the level of centrality can have an effect on linguistic categorization (Gong et al., 2012a) or the degree of diffusion of a variant in a population (Gong et al., 2012b). Using a communication game model where the probability of communication between agents is influenced by their mutual understanding, Gong et al. (2004) put forward the co-evolution of language and social structure, as well as the emergence of networks exhibiting small-world characteristics (see section 2).

Considering the speakers as individuals with different properties embedded in structured networks brings to the fore, on the one hand, the intrinsic complexity of the processes governing the amplification of variants induced by weak biases, and the contribution of individual variation to the complexity, robustness, and diversity of language, on the other. We present here a computational framework that allows us to perform an initial exploration of these questions, and we show that, in apparent contradiction with the “common sense” view (but see Navarro et al., 2018, for similar results in simpler social settings), even relatively weak individual biases affect the shared language of the whole community in structured communicative networks. Thus, far from being “swamped” by the tyranny of the majority, individual variation affects language and may even be one of the drivers behind the emergence of linguistic diversity and complexity. As Trudgill (2011a,b) points out, there are three decisive factors influencing the emergence of linguistic complexity: population size, degree of language contact, and the density of social networks—our framework naturally models the first and the third, while the second represents a natural future extension.

In section 2, we present our Bayesian agent-based model and the different parameters used in this analysis, such as the network type and size, the proportion of biased agents and the strength of the bias, the proportion of biased influencers, and the initial language of the society. In section 3, we investigate if, and how, the inclusion of biased agents in the network changes the language of the society, and the factors affecting the stabilization of the language. We close by discussing the limitations and implications of our findings, and suggest several future directions of study.

## 2. Methods

Our simulation framework is based on previously published models (Dediu, 2008, 2009) and has three main components: the *language*, the *agents*, and the *communicative network*. The language is modeled here as being composed of one (or more) *binary features*, that are obligatorily expressed in each individual utterance produced or perceived by the agents. We may think of these abstract features as representing, for instance, the use of the alveolar trill /r/ (value 1) or of a different r-like sound (value 0), the use of pitch to make a linguistic distinction (1) or not (0), having a subject-verb word order (1) or a verb-subject order (0), making a gender distinction (1) or not (0), using center embedding (1) or not (0), or any other number of such alternatives. Thus, if we take the /r/ interpretation, a set of utterances 1,1,1 might be produced by an agent that can trill without issues, a 0,0,0 by one that cannot, and 1,0,1 by an agent that either does not make the distinction or whose propensity to trill is affected by other factors (e.g., socio-linguistic or co-articulatory). Each agent embodies three components: language *acquisition*, the *internal representation* of language, and the *production* of utterances. The first concerns the way observed data (in the form of “heard” utterances) affect (or not) the internal representation of language that the agent has. The second is the manner in which the agent maintains the information about language. And the third, the way the agent uses its internal representation of the language to produce actual utterances.

We opted here for a *Bayesian model of language evolution* as introduced by Griffiths and Kalish (2007), and widely used in computational studies of language evolution and change (e.g., Kirby et al., 2007; Dediu, 2008, 2009, among others). In this approach, there is a *universe of possible languages* (discrete or continuous), *h* ∈ *U*, and an agent maintains at all times a *probability distribution* over all these possible languages. Initially, before seeing any linguistic data, the agent has a *prior distribution* over these possible languages, *p*(*h*), and, following exposure to new data (in the form of observed utterances), *d* = {*u*_1_, *u*_2_, …*u*_*n*_}, this probability is updated following Bayes' rule, resulting in the *posterior distribution* $p\left(h|d\right)=\frac{p\left(d|h\right)\cdot p\left(h\right)}{p\left(d\right)}$ that reflects the new representation that the agent has of the probability of each possible language *h* ∈ *U*. In this, *p*(*d*|*h*) is the likelihood that the observed data *d* was generated by language *h*, and *p*(*d*) is a normalization factor ensuring that *p*(*h*|*d*) is a probability bounded by 0.0 and 1.0. When it comes to producing utterances, we implemented two widely-used strategies (among, the many possible ones; Griffiths and Kalish, 2007): a language *h* can be sampled at random from the universe of possible languages proportional to its probability in the posterior distribution *p*(*h*|*d*)—a so-called *sampler strategy* (or SAM), or the agent can systematically pick the language *h*_*m*_ that has the maximum posterior probability *max*_*h*∈*U*_[*p*(*h*|*d*)]—a so-called *maximum a posteriori strategy* (or MAP).

In this paper, we model a single binary feature and consequently the utterances, *u*, collapse to a single bit of information, “0” or “1.” The observed data, *d*, become binary strings, and one of the simplest models of language is that of throwing a (potentially unfair) coin that returns, with probability *h* ∈ [0, 1], a “1” (otherwise, with probability 1 − *h*, a “0”). Thus, the universe of our languages, *h*, is the real number interval *U* = [0, 1] ⊂ IR, and the likelihood of observing an utterance *u* ∈ {0, 1} is given by the Bernoulli distribution with parameter *h*; for a set of utterances *d* = {*u*_1_, *u*_2_, …*u*_*n*_}, the likelihood is given by the *binomial distribution* with parameters *k* = |{_*u*_*i*_ = 1}*i* = 1..*n*_| (the number of utterances “1”), *n* (the total number of utterances), and $h:p\left(d|h\right)=Binomial\left(k,n,h\right)=\frac{n!}{k!\left(n-k\right)!}h^{k}{\left(1-h\right)}^{n-k}$, where *x*! = 1 · 2 · … ·(*x* − 1) · *x*; thus, we can reduce the set of utterances forming the data *d*, without any loss of information, to the number of “1” utterances (*k*) and the total number of utterances (*n*). In Bayesian inference we sometimes use the conjugate prior of a given likelihood, in this case, the Beta distribution defined by two shape parameters, α and β^1^, with probability density $f\left(x,\alpha ,\beta \right)=\frac{1}{B\left(\alpha ,\beta \right)}x^{\alpha -1}{\left(1-x\right)}^{\beta -1}$, where *B*(α, β) normalizes the density between 0.0 and 1.0. With these, the prior distribution of language *h* is *f*(*h*, α_0_, β_0_), with parameters α_0_ and β_0_ defining the shape of this distribution (see below), and the posterior distribution, updated after seeing the data *d* = (*k, n*), is *p*(*h*|*d*) = *f*(*h*, α_1_, β_1_), where α_1_ = α_0_ + *k* and β_1_ = β_0_ + (*n* − *k*); thus, the posterior distribution is also distributed Beta, with the shape parameter α “keeping track” of the “1” utterances, and β of the “0” utterances, and the Bayesian updating is reduced to simple (and very fast) arithmetic operations. When it comes to utterance production, a SAM agent chooses a value *h* ∈ [0, 1] from the *B*(α_1_, β_1_) distribution [i.e., proportional to *f*(*h*, α_1_, β_1_)], while a MAP picks the mode of the distribution, $h_{M}=\frac{\alpha_{1}-1}{\alpha_{1}+\beta_{1}-2}$; afterward, the agent uses this number between 0.0 and 1.0 as the parameter of a Bernoulli distribution (a coin throw) to extract a single “0” or “1” value with this probability—this value then is the utterance that the agent produces.

**Figure 1:**
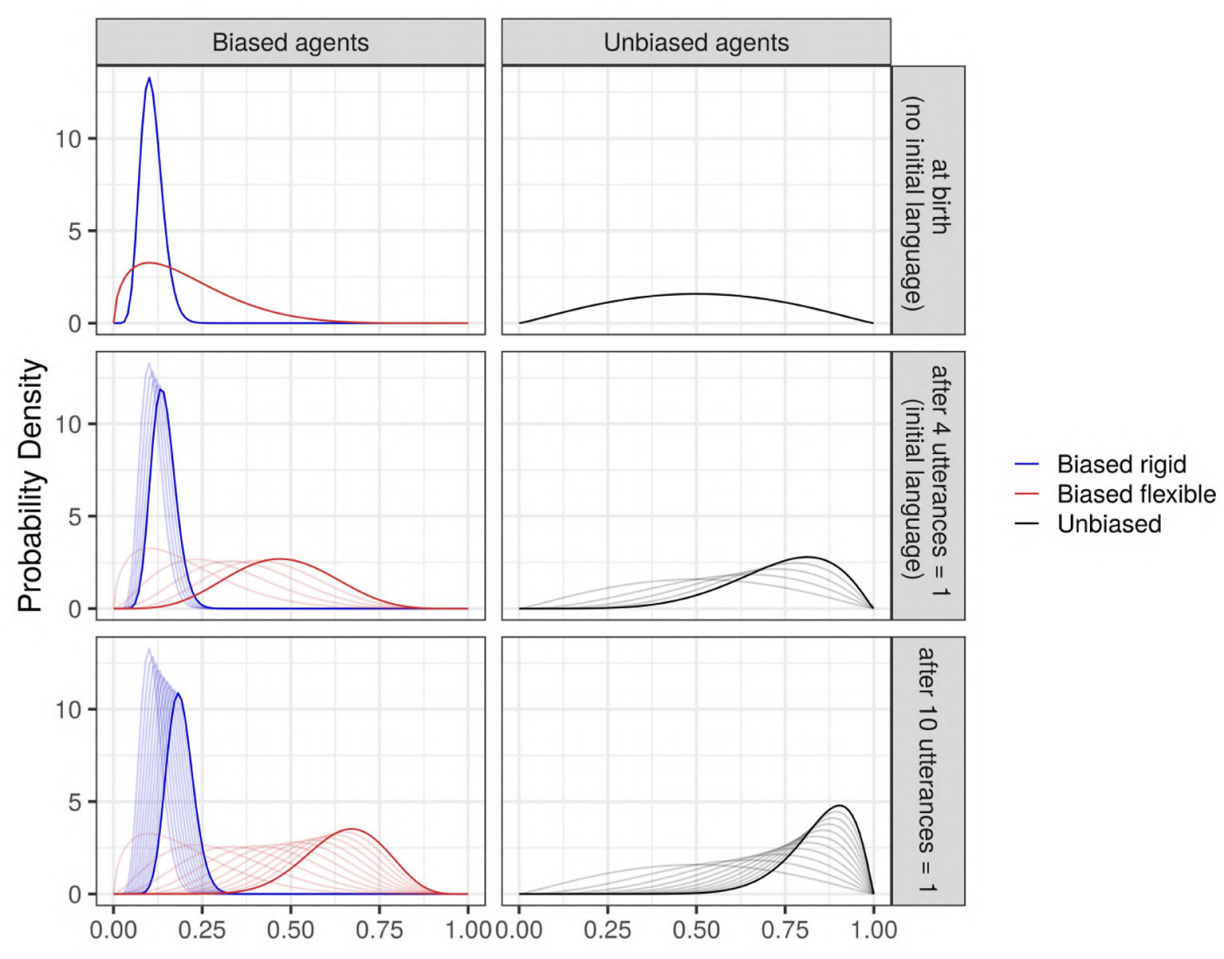
The evolution of some examples of Beta priors (thick solid curves) after seeing some data (utterances), to become successive Beta posterior distributions (thin curves). Blue: an agent strongly biased against the feature; red: an agent weakly biased against the feature; and black: an unbiased agent. **(Top)** The prior distributions before seeing any data (“at birth”), which corresponds to the case where no initial language exists in the society. **(Middle)** The Beta distributions updated after seeing *n* = 4 utterances all containing the value “1” (an initial language is present in the society; mildly biased toward “1”); **(Bottom)** An example to see the evolution of a Beta prior after seeing *n* = 10 utterances “1.” The evolution of the priors highly depends on the bias' strength: it is very fast for weak bias, and slower for strong bias.

This choice (Bernoulli/Beta) does not necessarily reflect how data is used by real humans in learning a language, but it has several major advantages, most notably its simplicity, transparency, and computational efficiency making it possible to run very large simulations on a consumer-grade computer in reasonable time (Dediu, 2009). Probably the most relevant here concerns the fact that the bias can be modeled only through the shape parameters of the prior Beta distribution, α_0_ and β_0_, as the likelihood function is fixed to the Binomial, and the utterance produced offers only a limited choice between SAM and MAP. However, the Beta distribution is flexible, and can be used to represent from (almost) flat (or uninformative) distributions, to extremely peaked and to “U”-shaped ones. Moreover, for unimodal cases, we can model not only the *mode* (i.e., the “preferred” value), but also the *variance* (i.e., how “strong” is this preference, operationally, how much data is needed to change the preferred value). In our simulations, we chose four different initial prior Beta distributions. The first one is almost flat, and centered around 0.5 (unbiased agents). In the three other conditions (biased agents), the agents have an intrinsic bias toward the variant “0” (the mode of their initial prior Beta distribution is 0.1), with various bias strength. This is visually captured by the “narrowness” of the Beta distribution, which may vary from quite flat and skewed to very narrow. See Supplementary Materials for more information for these parameters' choice, and Figure 1 for a visual representation of these distributions and of how they are updated upon seeing data. Note that here, the terms “biased agents” and “unbiased agents” do *not* refer to the mathematical properties of their Beta distributions. Instead, these terms refer only to the presence of an *intrinsic* bias, that is, a bias oriented toward the variant “0” before the agents hear any utterances (from the community convention, or from each other).

The *initial language* parameter corresponds to two situations (see Figure 1): on the one hand, it can model the (quite unrealistic) case where agents are born in a society without any pre-existing language or where they are not exposed to any linguistic input (*k*_0_ = 0, *n*_0_ = 0), so that the agents must create their first utterances based only on their prior bias. On the other hand, it can model the more common case where agents are born in a society with a pre-existing language already biased toward the use of the feature (*k*_0_ = 4, *n*_0_ = 4); this is modeled by presenting all the agents with the same 4 utterances “1” in the initial iteration, so that the first utterances generated by the agents are based both on their prior bias and the linguistic input from the society. In this analysis, the variant supported by agents having a bias (both strong or weak) is always the utterance “0.” In the case of absence of pre-existing language, biased and unbiased agents both start without input. In the case of a pre-existing language, biased and unbiased agents both start with an input (exposure to four utterances of “1”): thus, the “unbiased agents” start communicating with an internal distribution of language biased toward the community convention (the variant “1”). We remind here that the terms “unbiased” and “biased” used to describe the agents refer only to the presence or absence of an *intrinsic* bias acquired by the agents before they start hearing any type of utterance. For a visualization of this dynamic (see Figure 1).

**Figure 2:**
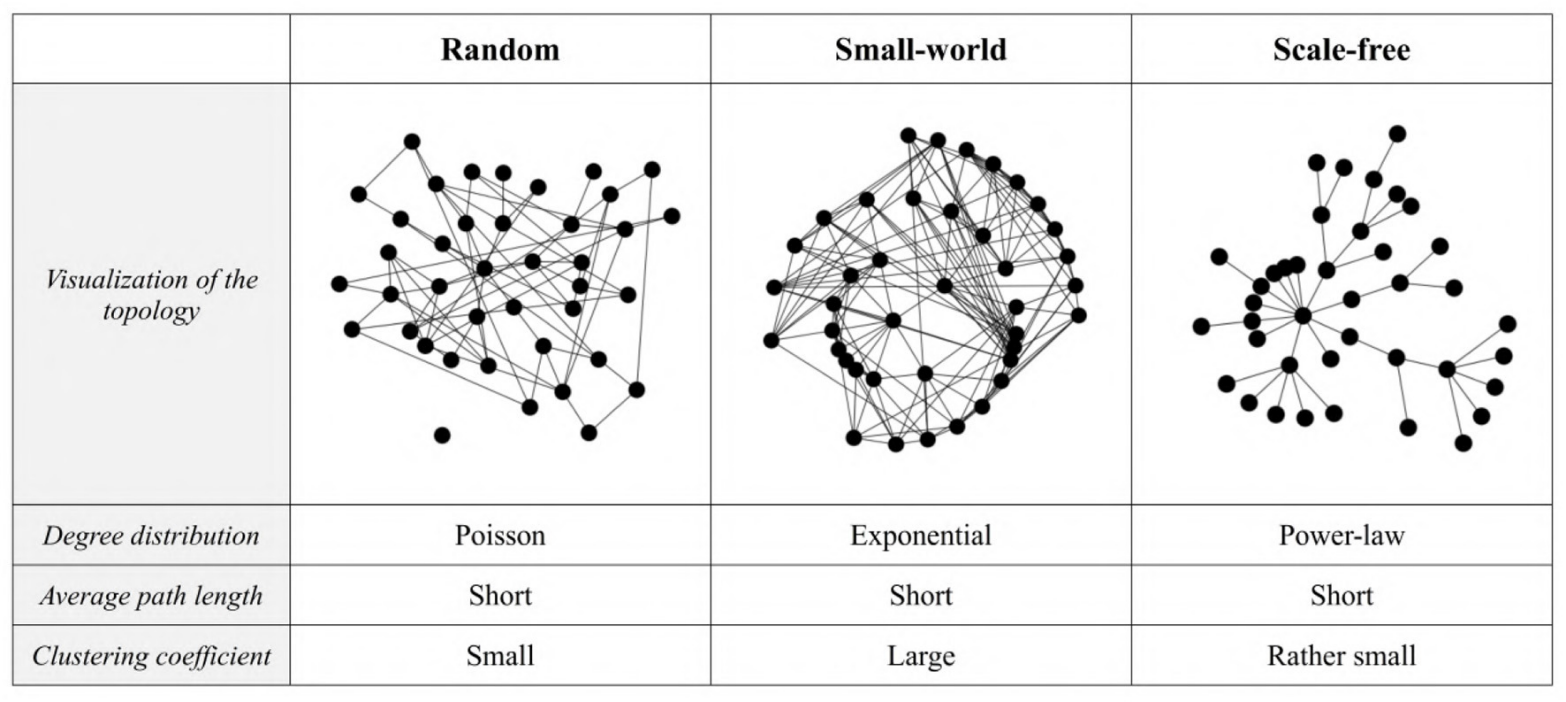
Examples of random, small-world, and scale-free networks with *N* = 40 nodes. The degree distribution is the probability distribution of the nodes' degree (the number of connections each node has to other nodes) over the whole network. The average path length is the average number of steps along the shortest paths for all possible pairs of network nodes. The clustering coefficient corresponds to the density of neighborhood, i.e., the degree to which nodes in a graph tend to cluster together (Watts and Strogatz, 1998).

Finally, the *network* represents the socio-linguistic structure of a community, and constrains the linguistic interactions between agents. The agents are the network nodes, and if there is an edge between two nodes then those two agents will engage in linguistic interactions. Note that we consider here only static networks: there is no change, during a run, in the number of nodes and the topology of the network (i.e., the pattern of edges connecting the nodes). The only change implemented in the properties of the nodes is the update of the posterior distribution, *p*(*h*|*d*), which is the agent's internal representation of the community's language, and does change with new data. Likewise, our model does not include directed nor weighted edges (i.e., the two connected agents can interact symmetrically, and there is no way to specify that two agents might interact “more” than others), but we do think that dynamic weighted directed networks are an important avenue to explore in the future. Here, we use three classes of network topology, namely *random, small-world*, and *scale-free* networks (Figure 2). The first is a highly unrealistic baseline model (Erdős and Rényi, 1959), where we specify the number of agents and the overall connectivity of the graph (in this model, always equal to 0.1^2^) giving the probability of adding an edge between any two nodes. However, as real-world networks are not generated randomly, we focus instead on small-world and scale-free networks. To generate the *small-world networks*, we use the classic “*beta* model” of the Watts-Strogatz algorithm (Watts and Strogatz, 1998): the algorithm first creates a ring of nodes, where each node is connected to a number *N* of neighbors on either side (here, *N* = 4), and then rewired with a chosen probability *p* (*p* = 0.1). This process leads to the creation of hubs and the emergence of short average path lengths. Small-world properties were popularized by Milgram (1967)'s “Six degrees of separation” idea, and are found in many real-world phenomena, such as social influence networks (Kitsak et al., 2010) and semantic networks (Kenett et al., 2018). Contrary to small-world and random networks, *scale-free* ones exhibit a power-law degree distribution: very few nodes have a lot of connections, while a lot have a limited number of links, and are found, for example, on the Internet (Albert et al., 1999) or in cell biology (Albert, 2005). To generate them, we used the preferential attachment algorithm (Barabási et al., 2000), which starts from a seed of nodes and gradually adds new ones; new links are created between the newly-added nodes and the pre-existing nodes following the rule that the more a node is connected, the greater its chance to receive new connections. Formally, the probability *p*_*i*_ that a new node is connected to node *i* is $p_{i}=\frac{k_{i}}{\underset{j}{\sum }k_{j}}$, where *k*_*i*_ is the degree of node *i*, and the sum is over all pre-existing nodes *j*.

**Figure 3:**
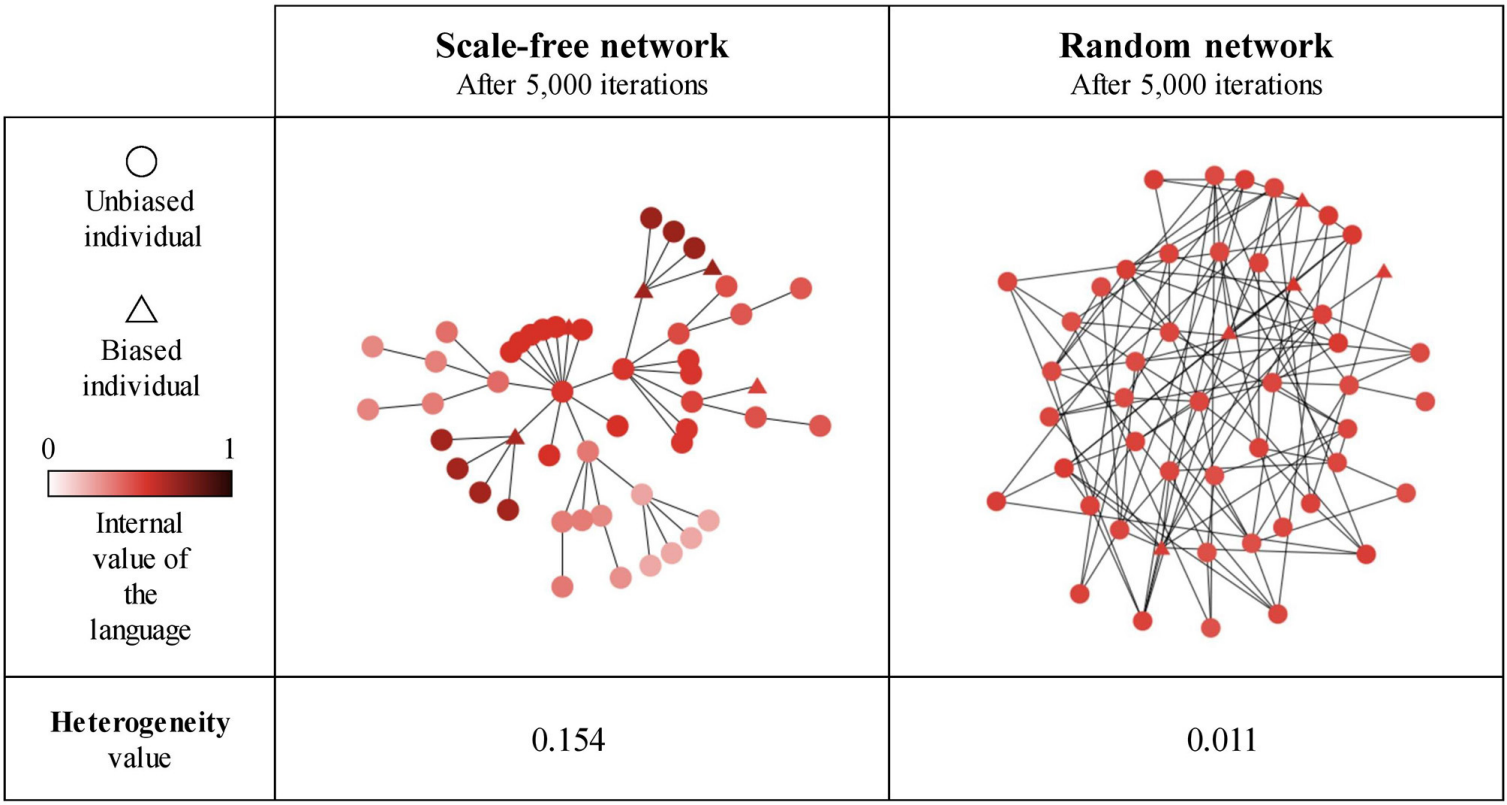
The evolution of the agents' internal representation of the language (node color) after 1 and 30 iterations in a scale-free network of *n* = 30 agents. In each iteration, all individuals speak and receive the utterance produced by all their neighbors. **(Left)** Initial state of the network before any interaction (i.e., reflecting the prior biases); **(Middle)** after 1 iteration; **(Right)** after 30 iterations.

Putting everything together (Figure 3), time is discretized into *iterations*, starting with iteration 0 (the initial condition of the simulation) in increments of 1. At each new iteration, *i* > 0, all agents produce one utterance, *u* ∈ {0, 1}, using their own internal representation of language and production mechanism (as described above). These utterances are “heard” by their neighbors (the “listeners”), who update their own internal representation of the language (also as described above) using a broadcasting mode. More precisely, in a given iteration, each agent is selected in turn in a random order (random permutation) and is allowed to produce one utterance (“speak”), utterance which is “heard” by all its network neighbors. The network is *asynchronous*, which means that the language value of listeners is updated immediately after hearing the speaker's utterance (in opposition to the *synchronous* network, where the language values of all agents are updated simultaneously at the end of each iteration, after all agents have talked). The choice of using an asynchronous network was driven by its lower computational cost; but the model was also run in a synchronous mode and the results were very similar (see Supplementary Materials). A special case is represented by the initial iteration *i* = 0, where the model can either start with the agents' own prior distributions (as defined, for each agent, by its own parameters α_0_ and β_0_, that may differ between agents), or we can “train” all agents on the same set of initial utterances *u*_1_, *u*_2_…*u*_*l*_ ∈ {0, 1} representing a pre-existing language shared by the whole community before the experiment starts. Note that not all agents in a network must share the same prior distribution (defined by α_0_ and β_0_) or utterance generating mechanism (SAM or MAP), and this is, in fact, one of the most important parameters we manipulate in our simulations. With time, due to how the Bayesian model was implemented, the internal distribution of agents' language becomes narrower and narrower (that is, the α and β parameters of their posterior distribution increase with time). Thus, utterances heard earlier have a larger impact on the internal representation of the language, compared with utterances encountered later. This, in turn, leads to a progressively reduced difference between the SAM and MAP strategies (see Supplementary Materials). In other terms, one could say that agents gain some confidence in their conception of the language, as they become more resistant to change with time.

With these, our simulation framework allows the manipulation of several parameters (see Table 1), but we limited ourselves to the conditions given in Table 2.

**Table 1:** Parameters defining our simulations (see also Table 2).

**Parameter**	**Variable name**	**Dependencies**	**Comments**
Network size, *N*	*size_net*	None	The number of nodes (i.e., agents); it is fixed for a given run
Bias location and strength, μ_0_ and λ_0_	*bias_strength*	None	See Figure 1
Utterance production mechanism, *UPM*	*learners*	None	
Frequency of biased agents, υ	*prop_biased*	None	The proportion of agents in the network that are biased; note that here we consider networks containing a single type of biased agents
Proportion of highest centrality agents that are biased, *TOP*	*influencers_biased*	Depends on υ	“Random” means that the biased agents are randomly placed in the network agent centrality, while “biased influencers” ensures that the top 10% highest centrality agents are biased (if υ ≥ 10%, otherwise υ)
Network type, *T*	*network*	None	Controls the class of network topology (see Figure 2 for examples).
Initial language, *k*_0_ and *n*_0_	*init_lang*	None	The total number of utterances (*n*_0_) and the number of utterances “1” (*k*_0_) presented to all the agents in the network in theinitial iteration *i* = 0 (see Figure 1)
Maximum number of iterations, *I*	*tick*	None	The maximum number of iterations to run
Number of independent replications per condition, *R*	*rep_id*	none	The number of independent runs (replications) for a given condition

**Table 2:** Values used in our analysis.

**Parameter**	**Values—Main study**	**Values—Systematic bias effects study**
Network size, *N*	10 (“tiny”) 50 (“small”) 150 (“medium”), 500 (“large”) and 1,000 (“very large”)	150 (“medium”)
Bias location and strength, μ_0_ and λ_0_	μ_0_ = 0.5, λ_0_ = 0.9 (“unbiased”) μ_0_ = 0.1, λ_0_ = 0.6 (“biased flexible”) μ_0_ = 0.1, λ_0_ = 0.1 (“biased rigid”) μ_0_ = 0.1, λ_0_ = 0 (“biased fixed”)	μ_0_ = 0.1 (biased) λ_0_ = 0.01 to 0.99, **in steps of 0.01**
Utterance production		
mechanism, *UPM*	*SAM* (“sampler”)	*SAM* (“sampler”)
	*MAP* (“a posteriori maximizer”)	
Frequency of biased agents, υ	0% (“fully unbiased”)	0–100%, **in steps of 1%**
	10%	
	30%	
	50%	
	100% (“fully biased”)	
Proportion of highest centrality agents that are biased, *TOP*	0% (“random”)	0% (“random”)
	10% (“biased influencers”)	50% (“biased influences”) 100% (“biased extremely influent”)
Network type, *T (for random and smallworld, same parameters as before)*	“Random” “Scale-free” “Small-world”	“Random” “Scale-free” “Small-world”
Initial language, *k*_0_ and *n*_0_	*k*_0_ = 0, *n*_0_ = 0 (“no initial language”) *k*_0_ = 4, *n*_0_ = 4 (“initial language”)	*k*_0_ = 4, *n*_0_ = 4 (“initial language”)
Maximum number of iterations, *I*	5,000	500
Number of independent replications per condition, *R*	100	50

*Due to computational costs, we performed two different types of analysis, using different sets of parameters. In both cases, all possible combinations of parameters were performed **R** times. While the variables in “main study” are used to understand which predictors affect the language value of agents and in which ways, the “systematic bias effect study” helps us understand to which extent the strength of the bias and the proportion of biased agents in the population affect the language value of the population after I iterations. See the text for more details*.

The size of social networks depends on how social networks are defined in the literature, they can vary between a few individuals and 5,000 or more individuals (Hill and Dunbar, 2003). Small groups, such as support cliques and sympathy groups, have in general a clustering of relationships between 5 and 15 people, while modern hunter-gatherer societies are usually described as containing from 30 to 50 individuals (Dunbar, 1993). As reported in the ethnographic literature, there are also higher-level grouping such as the mega-bands (500 individuals) and tribes (1,500–2,000 individuals) (Dunbar, 1998). Here, due to the computational costs involved, we were limited to 1,000 people in a population.

In order to test our hypotheses and to further explore the simulation results, we use the following *outcomes* (dependent variables): the *language value*, *l*_*a*_, the *heterogeneity* between groups, *h*_*s*_, and the *stabilization time*, *t*_*s*_.

### Language Value

The language value of an agent at a given moment varies between 0 and 1, and is the *mode* of the Beta distribution representing the internal belief of the agent concerning the distribution of the probability of utterances “1” in the language. Biased agents typically start with a lower *l*_*a*_ than the unbiased agents, thus favoring the variant “0.” We also define the language value of a given group of agents (for example, a community or the whole network) as the *mean of the language values* of all the agents in the group. We decided to focus on the language value observed after 5,000 iterations, because the language value was always stabilized after this period (see **Figure 13**). Given that our focus here is on understanding the effect of various parameters on the emergent language and the fact that we need to aggregate over multiple agents, we also estimate various types of *variation*. First, the *inter-replication* variation is estimated by computing the standard deviation of the language values obtained among the R replications after 5,000 iterations. It captures the influence of various sources of randomness on each particular run of a given condition, and it depends on the size and the type of network, the strength of the bias, and the initial value of the language (see Supplementary Materials). It is higher for random networks compared to scale-free and small-world networks, and higher for smaller networks. Furthermore, a weak bias and the absence of an initial language both amplify this variation. However, inter-replication variation is low, confirming the relevance of the mean of the agents' language values across the different replications. Second, *inter-individual* variation across the agents in a given network is an important outcome: we found that most biased and unbiased agents have very similar behaviors within their respective groups, justifying the use of the mean language values of the biased (*langval_biased*) and the unbiased agents (*langval_control*). We also computed the mean language value of the whole population (*langval_all*): even if there may be variation between groups (the biased vs the unbiased agents) and between agents, this value is a global indicator of the average language used in the population. Third, there are *differences between the unbiased and the biased agents (diff)*: here we used the signed difference between the mean language values of the unbiased agents and the mean language values of biased agents, as this gives very similar results to the much more computationally expensive method of computing all pairwise differences between all unbiased and biased agents.

### Heterogeneity Between Groups

**Figure 4:**
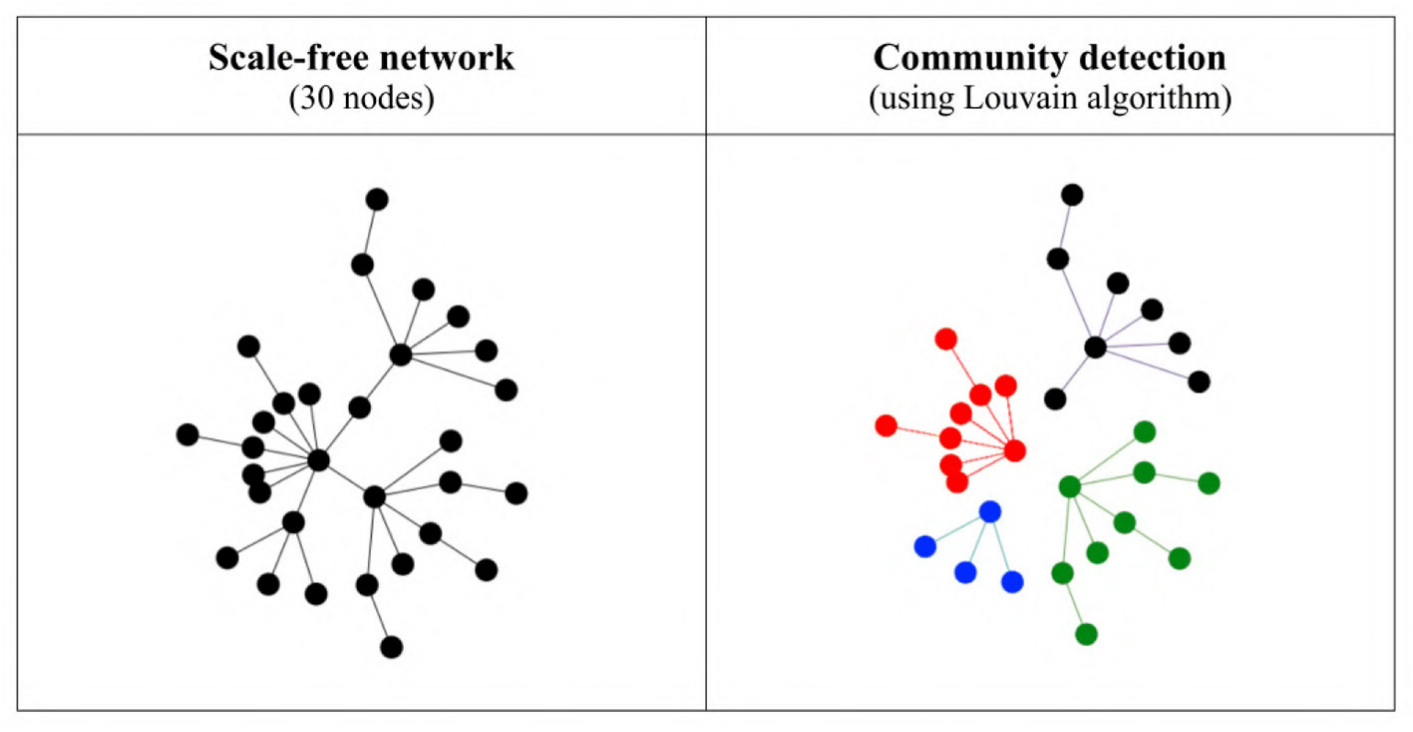
Structural communities detected in a very simple scale-free network using the Louvain algorithm. On the left is the original network with all connections, and on the right the four communities detected by the algorithm (shown in different colors and with the inter-community connections removed).

In order to study the possible differences in the language values of the agents belonging to different communities, we first detect the structural communities within the network using the Louvain community detection algorithm (Blondel et al., 2008) as implemented in NetLogo's nw extension package, which detects communities by maximizing modularity based on the connections agents share with each other, and not on the agents' language values (see Figure 4). Since the network is static, we then use the detected communities to compute the language value of each community for each iteration. Our measure of heterogeneity between groups is the standard deviation of these mean language values across communities. Thus, a low number indicates that all communities have approximately the same mean language value, whereas a high number indicates that the communities have rather different language values. (Note that this value was not computed for networks containing only 10 agents).

### Stabilization Time

**Figure 5:**
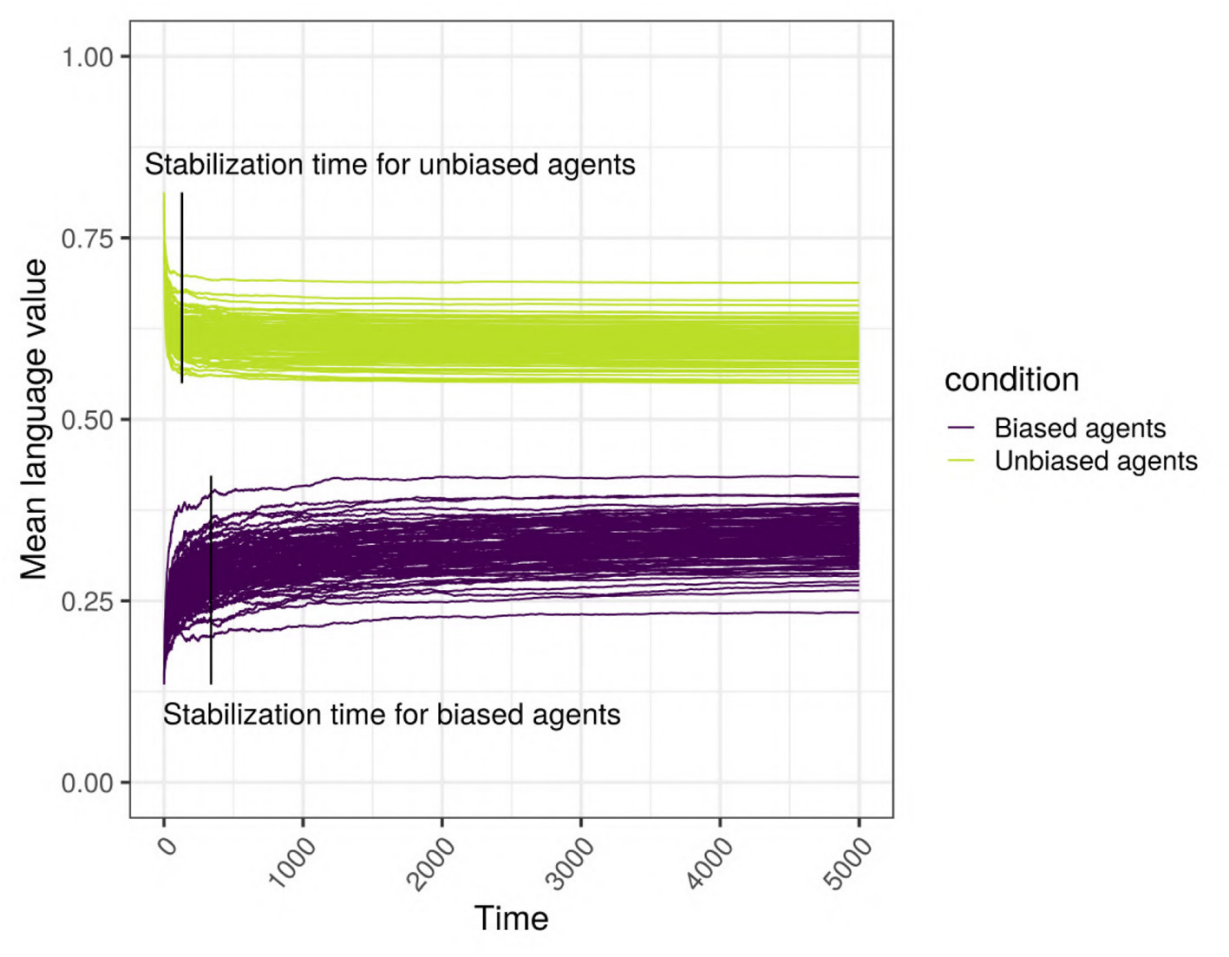
Stabilization times for the biased and the unbiased agents. This example uses a scale-free network with 500 agents, with SAM agents, where 10% of the top influencers are strongly biased, in the presence of an initial language.

Intuitively, stabilization time captures how long (in terms of interaction cycles) it takes for the language of a given network to reach a stable state. Given the inhomogeneous nature of the network, we consider two measures: the moment when *the language value of the biased agents* stabilizes (*stab_biased*) and the moment when the *language value of the control agents* stabilizes (*stab_control*) (Figure 5); these measures are estimated using the language values of their respective populations. The estimation is based on the method developed in Janssen (2018, p. 79) and used a fixed-size sliding window within which we estimate the change in the language value, we multiply this number by 10,000, round it, and stop if this number is equal to zero (i.e., the slope is within ±0.00001 of 0.0) for 50 consecutive steps. Practically speaking, the maximum number of ticks of our model is *nIterations* = 5, 000, and the size of the sliding window is ω = *nIterations*/10. For a given window, we estimated the change, *t*(*e*_*g*_) using the following formula, where *g* is the number of iterations.

(1) $$\begin{array}{l} t\left(e_{g}\right)=\frac{\left(e_{g+w}-e_{g}\right)}{\omega }*10,000 \end{array}$$

On the rounded *t*(*e*_*g*_) values, we find the first value of *g*, *g*_*stabilization*_, when the rounded value of *t*(*e*_*g*_) = 0, and we stop if for 50 consecutive steps (i.e., *g* ∈ [*g*_*stabilization*_..(*g*_*stabilization*_+50)]), there is no change, *t*(*e*_*g*_) = 0; in this case, the stabilization time is the first moment where there was no change, namely *g*_*stabilization*_.

Our framework is implemented in NetLogo 6.1.1 (see here), the experiments were run on an Intel(R) Xeon(R) W-2255, 64 Gb RAM system under Ubuntu 18.04, and the results analyzed using R 3.6.3/Rstudio 1.4 on machines running Ubuntu 18.04 and macOS 10.15 (Catalina); the full source code and results are available at Github (mathjoss/bayes-in-network). The runtimes were between 6 h (scale-free networks) and 3 days (random networks) for the main analysis. It is possible to study networks up to 2,000 agents, but above 1,000 agents, the computations are too slow and would require access to a computer cluster.

## 3. Results

We present here a summary of the most relevant results for our discussion, with the full results, including the actual data and R code, being available in the accompanying Supplementary Materials, to which we also make explicit reference in some cases. Note that the predictors are systematically standardized (z-scored, with mean 0 and standard deviation 1) for all regression analyses (so that we can directly compare their regression slopes, β), and the *p*-values of all the pairwise tests are corrected for multiple testing using Bonferroni's method.

### 3.1. Can a Minority of Biased Agents Affect the Language of the Whole Population?

**Figure 6:**
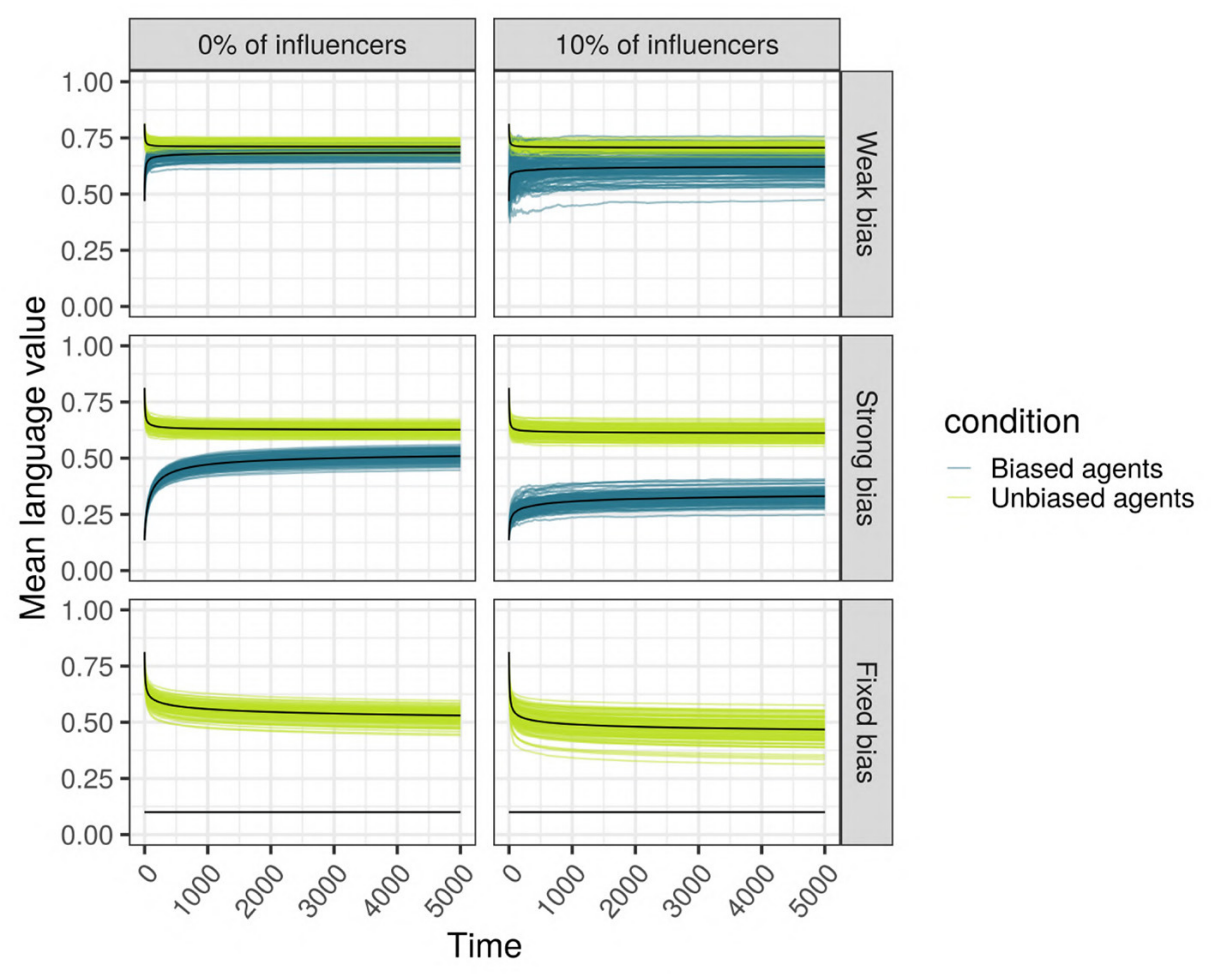
Language (vertical axis, as language values) is changing across time (horizontal axis, in ticks) in a scale-free network with 500 SAM agents of which 10% are biased. Each individual curve represents the mean language value of the biased minority (purple) and the unbiased majority (light green) for 100 independent replications. Top: The minority is strongly biased; bottom: the minority is weakly biased. **(Left)** The biased minority is not overrepresented among the most influential agents in the network; **(Right)** the 10% most influential agents are occupied by biased agents.

We hypothesized that the bias of a minority of agents present in a population is not swamped by the unbiased majority, but contributes to the language of the whole population. More concretely, the population containing biased agents will use more of the variant “0” compared to the population without any biased agents. As an example, Figure 6 shows the change across time in the language value of a scale-free network with 500 SAM agents, of which 10% are biased. It can be seen that the language of the network is clearly affected by the biased minority, in that even the language value of the unbiased majority is “attracted” away from its initial language toward the language value of the biased minority, resulting in an overall language, qualitatively somewhere in between the unbiased majority's and the biased minority's languages.

**Figure 7:**
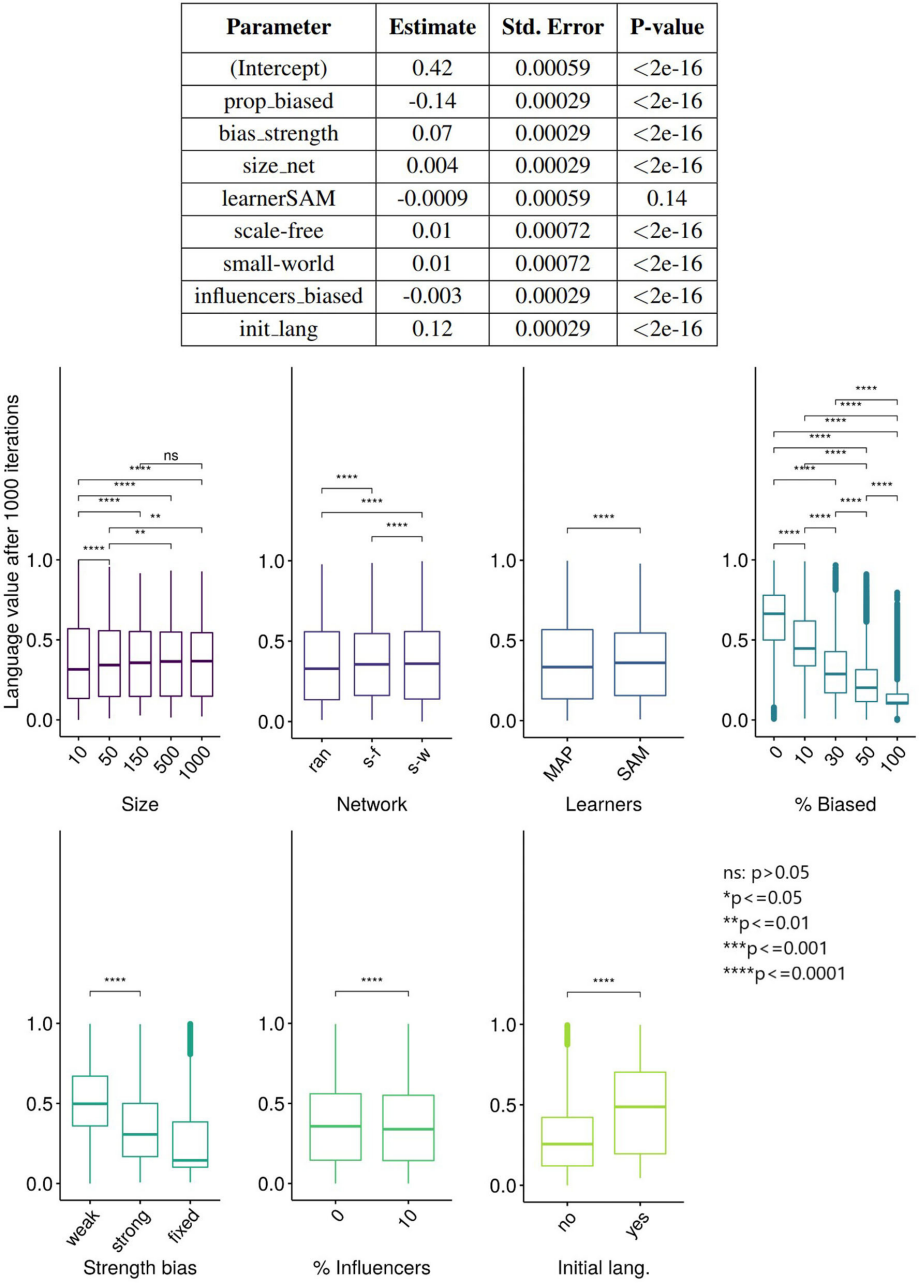
**(Top)** The results of the linear regression of the language values of all agents after 5,000 ticks on various parameters. Degrees of freedom (df) = 119,991, adjusted R2 = 79.4%. The variable “learner” is a factor with two levels (SAM and MAP) and treatment contrast, with the baseline level MAP included in the intercept. The same applies to the variable “network,” with “random” being the baseline level included in the intercept. **(Bottom)** The results of unpaired Wilcoxon tests (with adjusted significance stars, where ns: *p* > 0.05; ^*^p < = 0.05; ^**^p < = 0.01; ^***^p < = 0.001; ^****^p < = 0.0001) between the language values (vertical axis) across multiple replications vs. the parameters (horizontal axis).

But what factors, and how exactly, allow the minority's variant to be expressed in the language of the population? We used linear regression using lm function (R Core Team, 2020) to investigate the influence of the parameters on the language values of all the agents after 5,000 ticks, and the results (Figure 7) show that almost all variables have a statistically significant effect on the language value, but only the proportion of the population that is biased (*prop_biased*), the strength of the bias (*bias_strength*), and the initial language value (*init_lang*) have large effect sizes.

A different quantification of the influence of these parameters is shown in the bottom part of Figure 7. Interestingly, we found that the effect of *influencers_biased* is negligible. However, it has a small interacting effect with network type, the bias' strength and the percentage of biased agents: the language value of the population in scale-free networks with strongly biased agents is lower when there are 10% of biased influencers (note that no interactions were entered in this regression model; however, interactions effects are available in the Supplementary Materials). A very small effect size is also observed for the network type: the language value of the population is relatively similar for scale-free, small-world and random networks.

**Figure 8:**
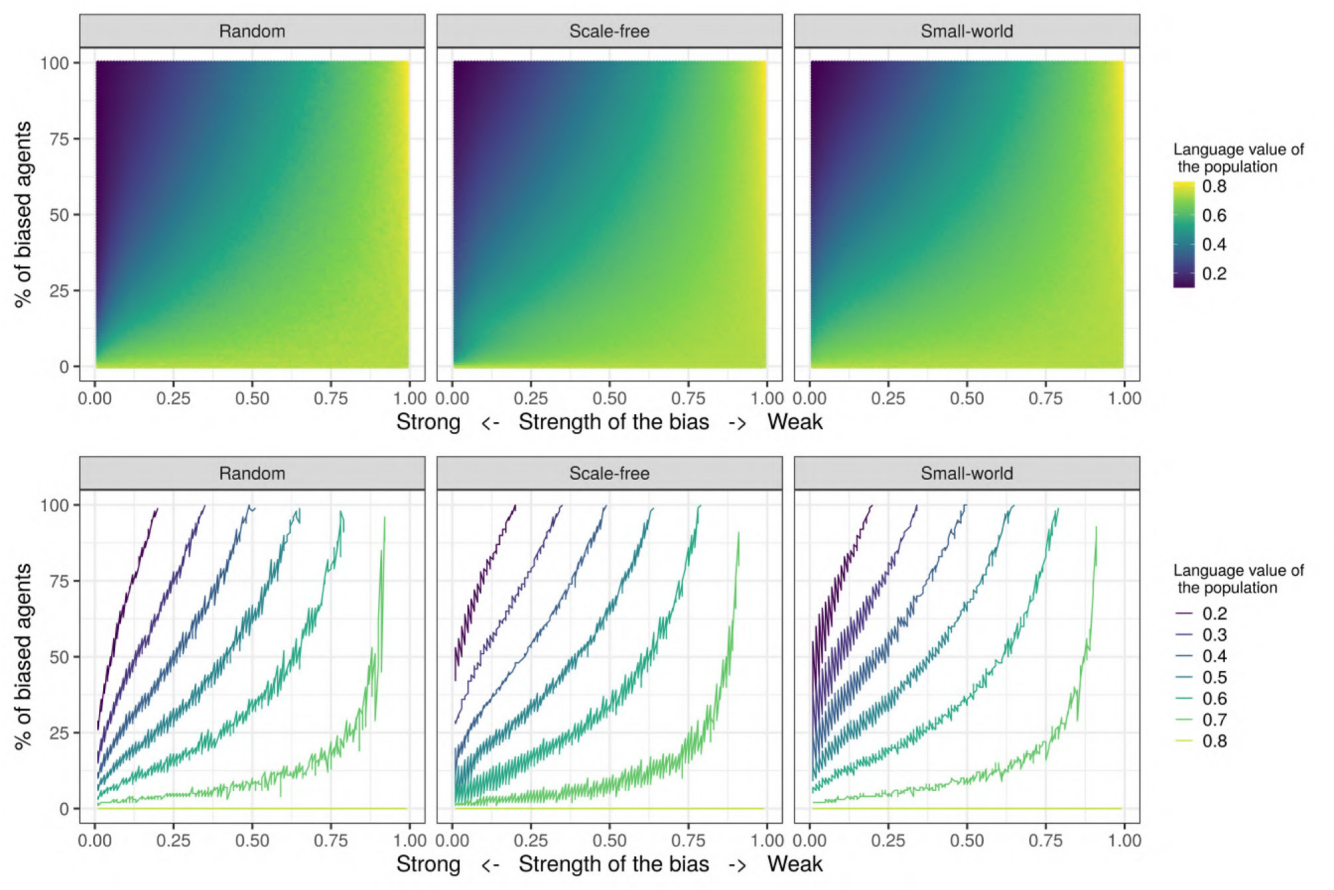
**(Top)** The aggregated mean language value (color) function of bias strength (horizontal axis, systematically varying between 0.0 and 1.0 in steps of 0.01) and proportion of biased agents in the population (vertical axis, varying between 0 and 100% in steps of 1%). For each of the possible combinations of these two parameters, a colored dot indicates the aggregated mean language value of the population. **(Bottom)** The different language values are plotted with isolines, in contrast to the continuous scale used in the figure on the top. The isolines are the maximum values of the combination of *bias_strength* and *prop_biased* for a set of language values, and color represents the value of the language isolines. We used a network with an initial language, containing 150 SAM agents.

Figure 8 shows the joint influence of the proportion of biased agents and the strength of the bias on the population's language value for the set of values in the “Systematic bias effects study” (see Table 2). We decided to further investigate the effect of these two parameters due to their large effect sizes (see Figure 7). In this study, we ran 50 independent replications for each of all the possible combinations of the bias strength (going from 0.0 = very strongly biased to 1.0 = very weakly biased, in steps of 0.01) and the proportion of biased agents in the population (going from 0 to 100% in steps of 1%). For each replication, we computed the mean language value of the population after 500 iterations, and we then averaged the 50 independent replications for each combination by taking their mean: for example, the averaged mean language value of the population for the condition {*bias_strength*=0.70 & *prop_biased*=35} is 0.67, but is 0.22 for the condition {*bias_strength*=0.15 & *prop_biased*=80}. As Figure 8 shows, in general, the aggregated mean language value progressively increases with the proportion of biased agents and the strength of the bias.

In order to better visualize the shape of the relationship between the bias strength and frequency (i.e., linear or not), and also to check if the proportion of biased influencers impacts the results, we also show the set of *isolines* for the mean language value of the population (see Figure 8). These isolines are defined as the maximum values of the combination of *bias_strength* and *prop_biased* for a given set of language values. Interestingly, the relationship between the strength of the bias and the proportion of biased agents is relatively linear when the proportion of biased agents is high and/or when the bias is strong, but becomes nonlinear for low frequencies of the biased agents and for weak biases. In this latter case, the effect of biased agents on the language value of the population is much stronger than expected. Moreover, this analysis helps to understand under what conditions an initial language strongly favoring “1” may change to a language favoring the variant “0”: while only in populations with a large proportion of strongly biased agents (>50%) does the language strongly favor “0” (a language value of 0.2), it is enough for only 15–20% of the populations to have a strong bias for the language to reach a moderate preference for “0” (language value of 0.4). However, note that while these particular values critically depend on the initial language (i.e., the number of initial utterances and the distribution of “0” and “1” utterances), they do support qualitative inferences concerning the influence of biased agents in a population.

Taken together, these results clearly show that biased agents, even if in minority, can have an impact on the language of the whole population: indeed, the bias of the agents is far from being swamped by the majority! In the remaining sections we will unpack the reasons for these findings by exploring different hypotheses. First, as we could see in Figure 6, we test in which way the biased and the unbiased agents influence each other, and we suggest that the biased agents “drag down” the language value of unbiased ones. Second, we hypothesize that biased agents maintain a trace of their bias in their language, even after interacting with the unbiased agents; this thus “lowers” the mean language value of the whole population, and makes the biased agents use a different language compared to the unbiased agents, the different types of languages “cohabiting” together in the same population. Third, we explore the hypothesis that inter-individual variation within a population leads to the emergence of linguistic communities using different languages. We note that these hypotheses are not mutually exclusive, but can be all true to some extent, beyond the framework provided by the rather simple and naive modeling approach proposed here.

### 3.2. How Do the Biased Agents Affect the Language of the Whole Population?

#### 3.2.1. Hypothesis 1: The Biased Agents Affect the Unbiased Agents (the “Language Compromise” Hypothesis)

**Figure 9:**
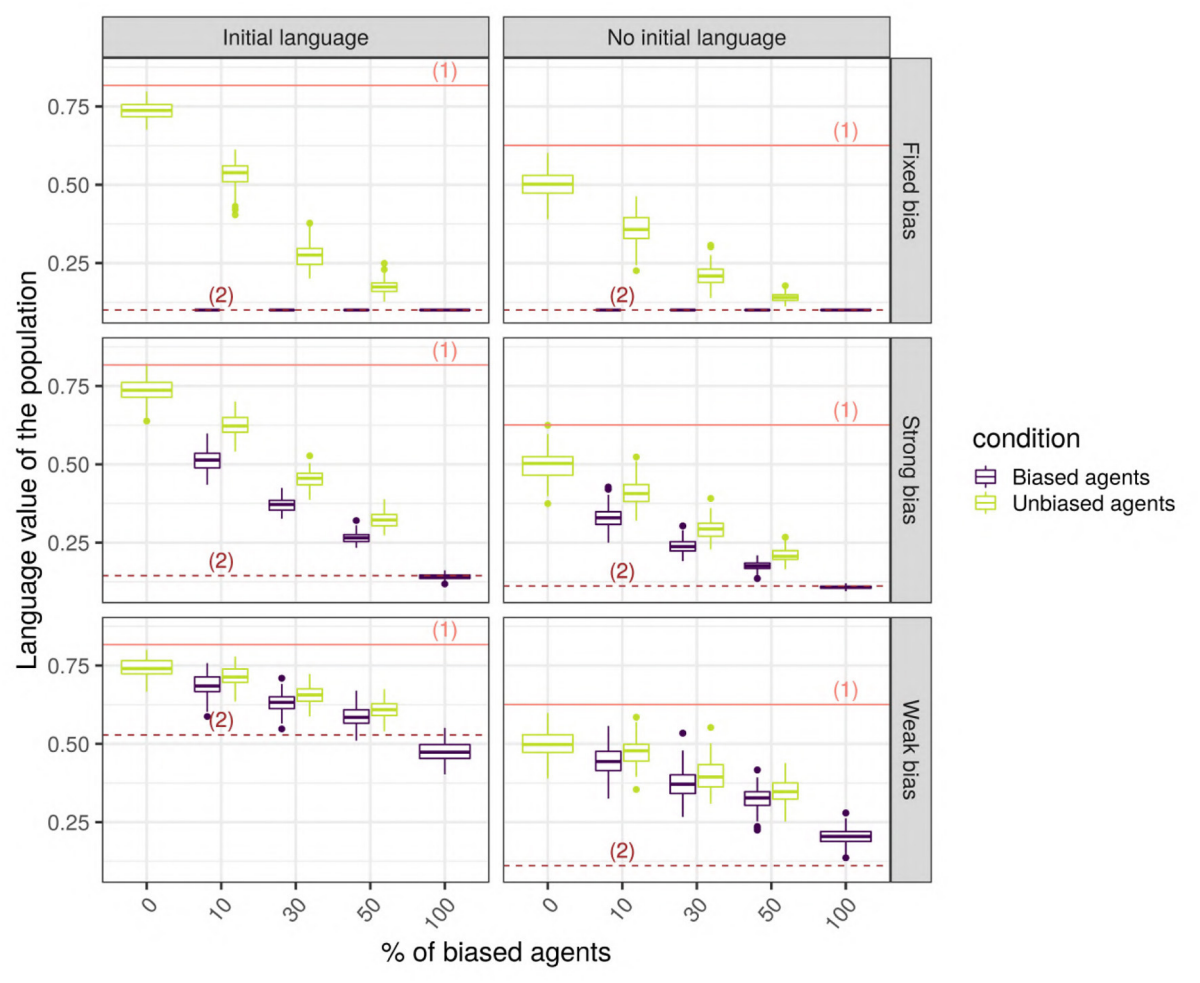
The final language value of the whole population for a scale-free network with 150 SAM agents. The solid line (1) shows the initial value of the language for the unbiased agents, while the dotted lines (2) show the initial value of the language for biased agents. The horizontal axis shows the different cases considered (combinations of bias strength and proportion of biased agents in the populations), the vertical axis is the language value of the population, and the colored boxplots show the distribution of the language values among the biased (blue) and unbiased (green) agents.

When biased and unbiased agents are mixed together in a network, their language values, very different at first, tend to converge toward a common language value (Figure 9). Adding an initial language to the society drastically changes the language value of the population, which is not surprising since the unbiased agents, after hearing the four initial utterances of “1,” learn a high language value, while the biased agents will shift toward intermediate language values. In the following, we focus on the more realistic case where an initial language is present. Indeed, even if the case without an initial language is interesting from a theoretical perspective on the origins of linguistic systems, we assume that, normally, the individuals are born embedded in a society with a pre-established language system.

We performed unpaired Wilcoxon tests comparing the language values of the unbiased agents in a population with biased agents to those in a population without biased agents, for all possible combinations of parameters, and we corrected the *p*-values for multiple testing using the Bonferroni method. These adjusted p-values show that, in the vast majority of the combinations (93%, 670/720), the language values of the unbiased agents in a society with biased agents are significantly different from those of a homogeneous unbiased population. Among the 50 replications with no significant differences, 33 were networks with only 10 agents, and the remaining 17 were random or small-world networks with a low proportion of weakly biased agents. In these simulations, the biased agents are distributed randomly in the network, so that both the biased and the unbiased agents are likely to hear utterances that will change the posterior probability of their language value: each utterance “0” heard by an unbiased agent will slightly modify the distribution of its internal language value.

This hypothesis is supported: agents within a finite population tend to share quite a similar language, which means that the biased agents do affect the unbiased agents, and vice-versa. However, are the inter-individual differences always swamped by communicating within such a population? Thus, does communication necessarily force conformity among agents? We hypothesize that this is not the case, and that instead the biased agents manage to maintain a trace of their initial bias in their language, even after interacting repeatedly with the unbiased agents.

#### 3.2.2. Hypothesis 2: The Biased Agents Do Maintain a Trace of the Initial Bias in Their Language, Even After Repeatedly Interacting With the Unbiased Nodes (the “Bias Resilience” Hypothesis)

**Figure 10:**
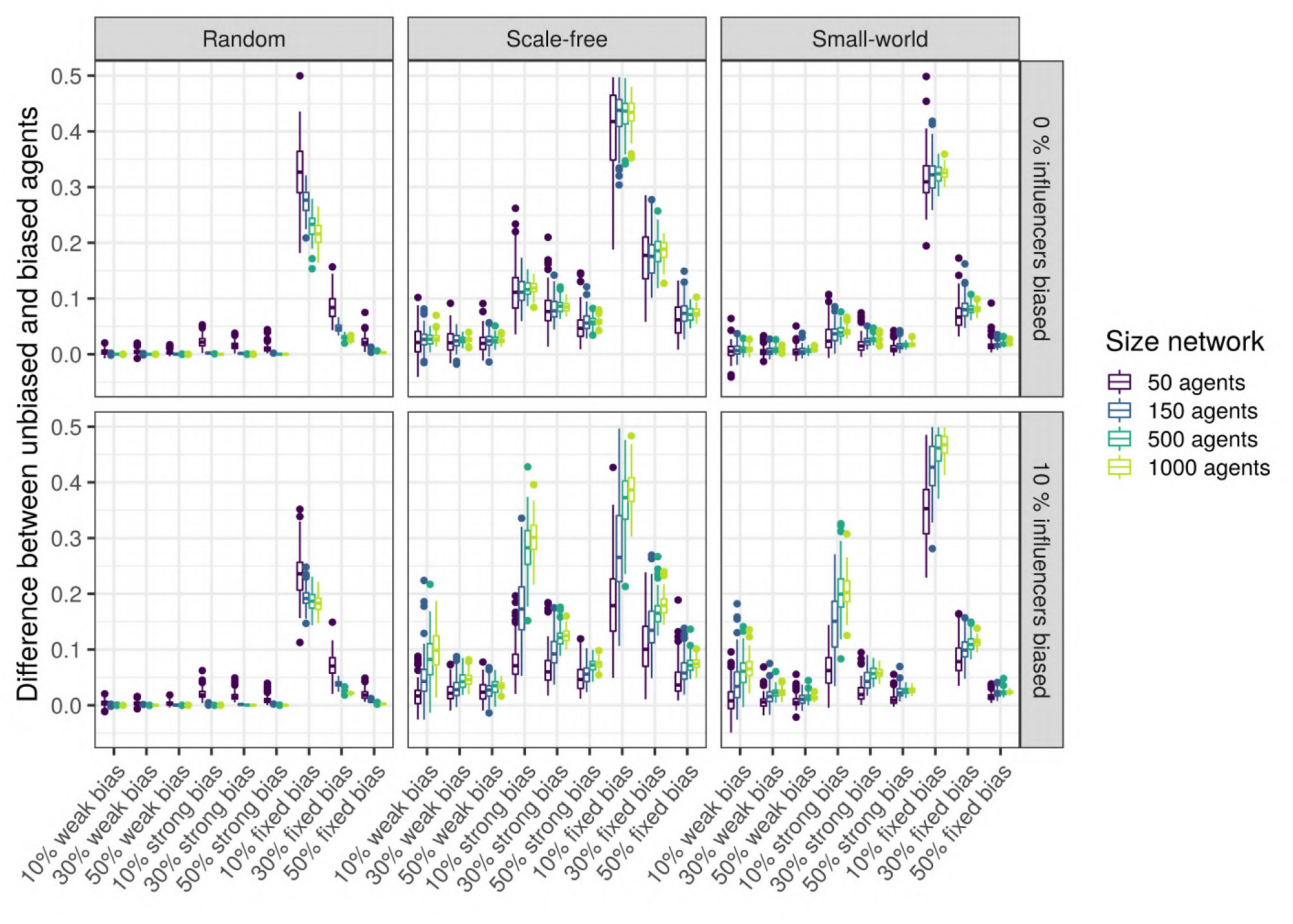
Top row: The difference between the languages of the unbiased and the biased agents after 5,000 iterations, function of network type and influencers biased (panels), size (color), and bias frequency and strength (horizontal axis). We used SAM agents, there is no enrichment of biased agents among the top influencers, and agents were exposed to an initial language.

To test this hypothesis, we measure the difference in the language values between the unbiased agents and the biased agents after 5,000 iterations: the higher the signed difference, the more different the languages used by the two types of agents are. A multiple regression analysis shows that only the network type (*network*) and size (*size_net*), the proportion of biased agents (*prop_biased*), and the strength of their bias (*bias_strength*) have a large effect size (Figure 10 zooms in on their effects and the Supplementary Materials). We observe that in random networks, this difference is very small, while in scale-free and small-world networks, this difference is present and depends on the proportion of biased agents and the strength of their bias.

We also performed unpaired Wilcoxon tests comparing the language values of the biased and the unbiased agents in all sets of combinations, using Bonferroni multiple testing correction. The adjusted *p*-values are almost always significant for scale-free networks (except for 44 networks with 10 or 50 agents, often weakly biased); significant for 52% of the small-world networks, especially for big networks with strong biases; however, most random networks do not show a significant difference, with the exception of a few very small networks. Particularly in scale-free networks, the proportion of the top-influencers that are biased also affects the difference in language values between the unbiased and the biased agents (see Figure 10), especially in networks with 10% of strongly biased agents.

These results allow a more nuanced view of the first hypothesis' conclusions: while the biased agents do affect the unbiased agents and all agents do tend to reach a language compromise, the biased agents still manage to maintain a trace of their initial bias in their language, even after interacting with the unbiased agents.

#### 3.2.3. Hypothesis 3: The Emergence of Linguistic Communities With Different Languages (the “Linguistic Polarization” Hypothesis)

**Figure 11:**
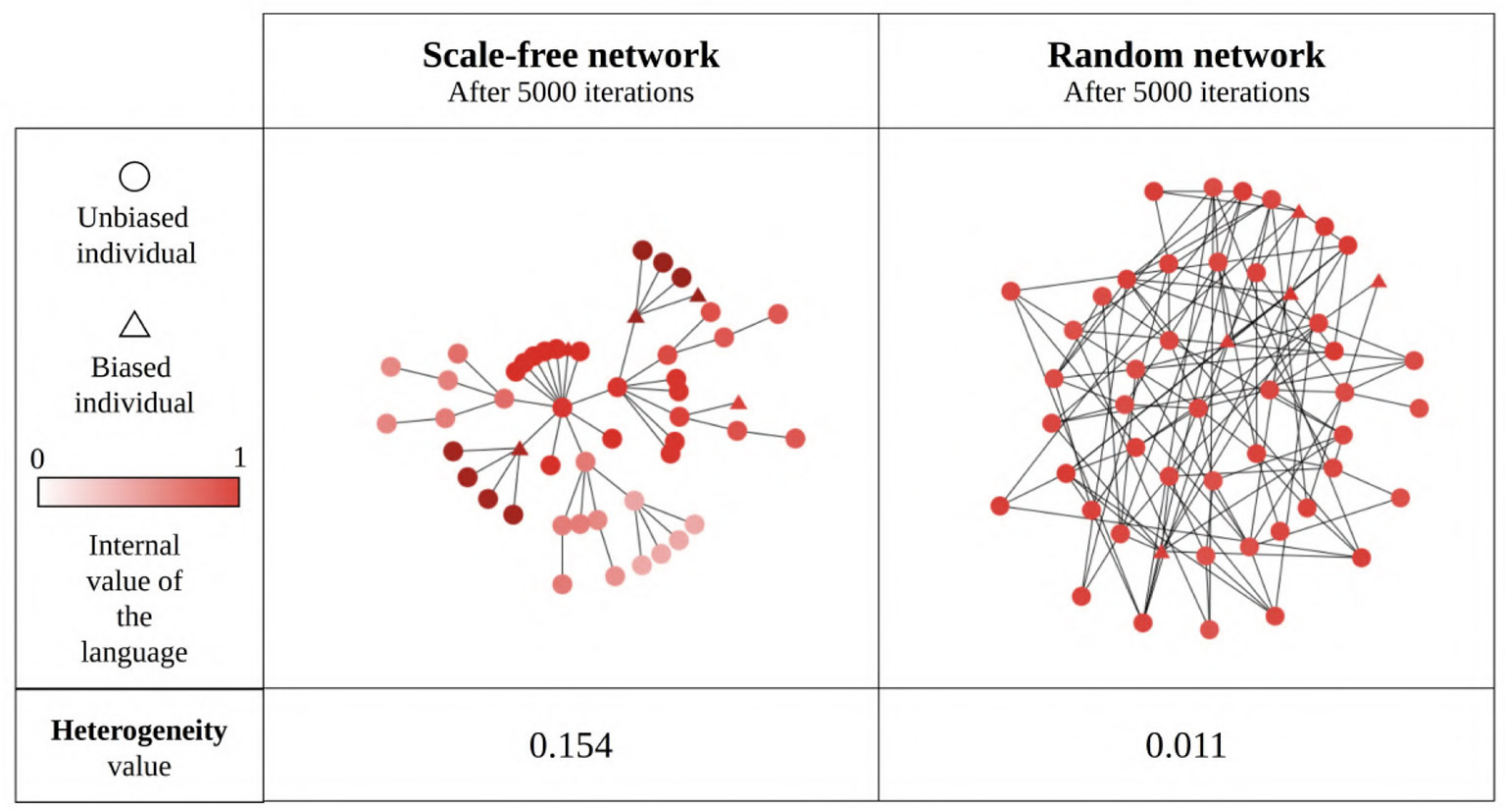
An example of linguistic community emergence in scale-free **(left)** and random **(right)** networks with 50 SAM agents, 10% of which are strongly biased (triangles) while the remaining 90% are unbiased (circles). Agent colors represent their language value; agents were exposed to an initial language.

**Figure 12:**
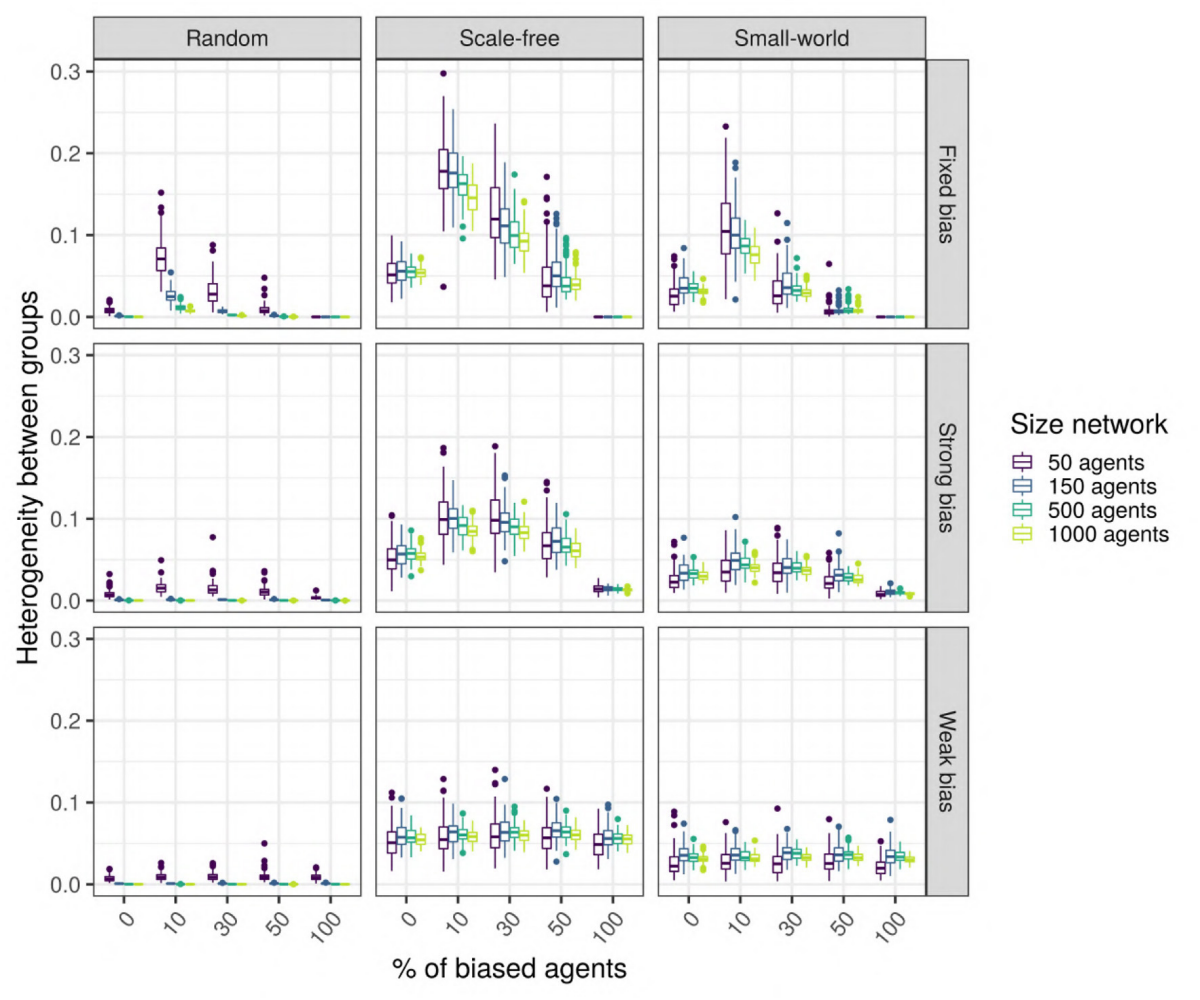
The difference in heterogeneity between linguistic communities function of network type (columns) and size (colors), and bias strength (rows) and frequency (horizontal axis). The networks contain SAM agents, no influencers are biased, and there is an initial language.

Our results so far show that network type and size generally influence the language value of the population, suggesting that this may be due (in part) to the emergence of linguistic communities using different languages within the network (Figure 11). We estimate the existence of such linguistic communities through the heterogeneity of the language values between structural communities in the network (as detected by the Louvain community-detection algorithm). A multiple regression analysis (see Supplementary Materials for full results) shows that only network type has a big effect size on the heterogeneity between communities (Figure 12). It can be seen that the linguistic communities do not generally emerge in random networks^3^. On the other hand, scale-free and small-world networks tend to behave differently: even when there are only unbiased agents in the network, we can see the emergence of linguistic communities differing in their language, suggesting that network structure itself favors the emergence of linguistic communities. However, if the network contains only strongly biased agents, all agents will share the same language before and after interacting with each other, precluding the emergence of linguistic communities. The maximum heterogeneity between communities is found in scale-free networks when there is a minority of strongly biased agents.

We performed unpaired Wilcoxon tests comparing the heterogeneity of, on the one hand, the unbiased agents in a population with biased agents, to that of the unbiased agents in a population without biased agents, on the other, for all possible combinations of parameters, and we corrected the *p*-values for multiple testing using the Bonferroni method. These adjusted *p*-values show that, in scale-free networks with a strong bias, having biased agents in the network significantly affects the emergence of linguistic communities (86%, 83/96); this is also true, to a smaller extent, for small-world networks with strongly biased agents (75%, 72/96). However, in scale-free and small-world networks containing weakly biased agents, only about half of the time the comparisons are significant (45% for scale-free, and 54% for small-world); thus, the heterogeneity observed in these networks is probably mostly due to the structure of the network itself.

Thus, the hypothesis 3 is supported by our results to a certain extent: heterogeneity between linguistic communities seems to naturally emerge in heterogeneous scale-free and small-world networks but only with agents who are not too weakly biased; moreover, strongly biased agents amplify the language differences between linguistic communities in scale-free networks.

#### 3.2.4. Putting the Three Hypotheses Together: Even Rare and Weak Biases Matter!

The results show that the bias, even in a minority, is not swamped by the majority: instead, it affects the language of the whole population. As the agents are interacting, the biased and the unbiased agents are influencing each other's language: consequently, the biased agents “pull” the language values of the others toward the value preferred by their bias. In random networks, all agents eventually agree on the same language value (unless the network is very small), but, due to their internal structure, both small-world and scale-free networks see the emergence of linguistic communities diverging in their languages. Moreover, in scale-free networks (and, to a smaller extent, also in small-world networks), the biased agents do retain a trace of their bias in language, and, when strongly biased, they help amplify the differences between linguistic communities. Thus, network structure is a key parameter for understanding the structural properties of the emergent languages, but does it also affect the speed with which the language reaches its stable state?

### 3.3. When Does the Language Stabilize?

**Figure 13:**
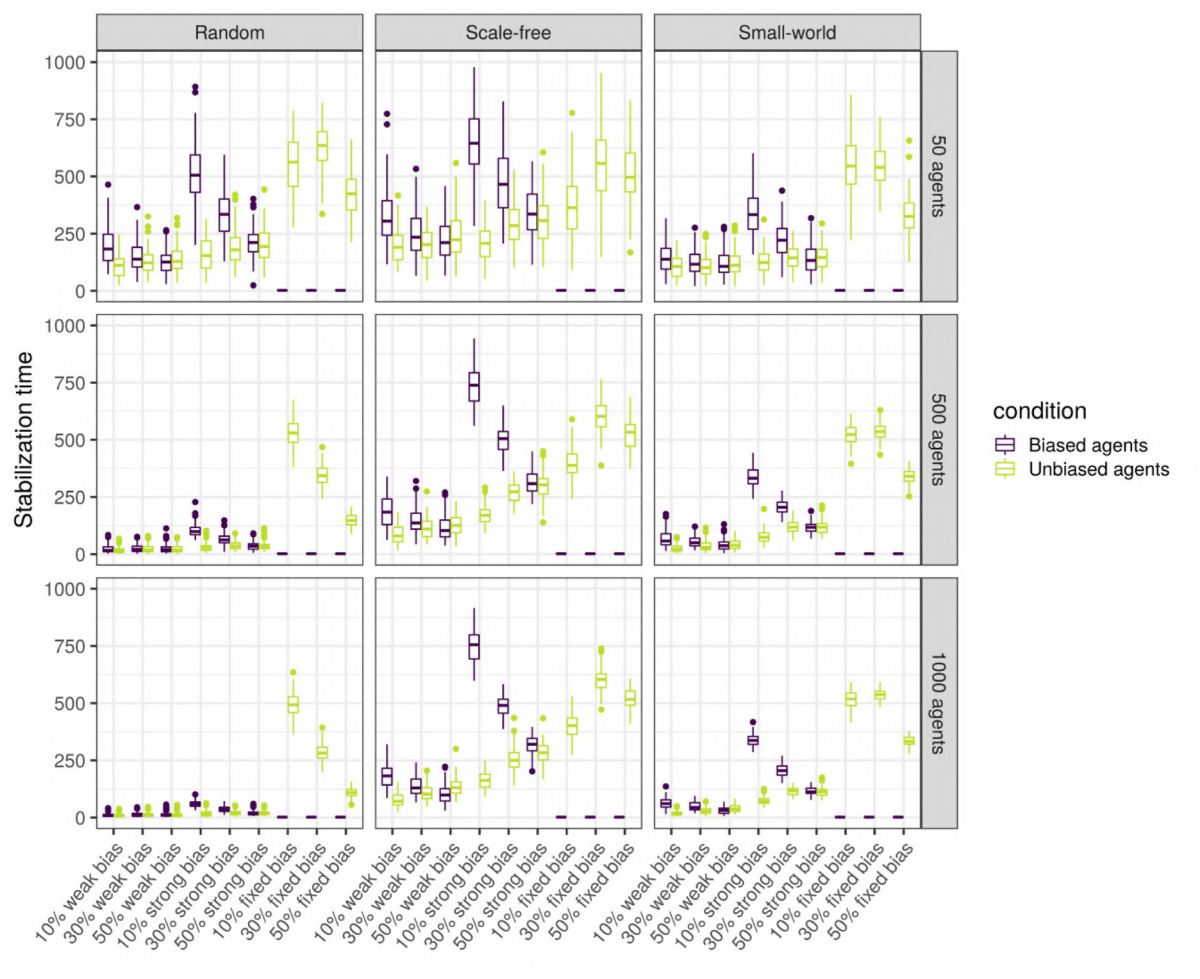
Stabilization time for the biased and the unbiased agents (color), in different types of networks (columns) with two different sizes (rows), for various bias frequencies and strength (horizontal axis). The agents are SAM, there are no biased influencers, and there is an initial language.

To answer this question, we analyse the agents separately depending on their type (unbiased vs. unbiased) as the languages of the two types might stabilize at different times. Thus, we performed linear regressions for the biased agents, and for the unbiased agents separately (see Supplementary Materials for full results). While most of the variables have a significant effect, only the proportion of biased agents, the strength of the bias, and the size and type of network have a large effect size. As we can see in Figure 13, there is an interaction between network size and type: while stabilization time decreases with size in random networks, it is stable in small-world and scale-free networks. The stabilization time for biased and unbiased agents in all types of networks with weakly biased agents is approximately the same. However, for networks with strongly biased agents, the proportion of biased agents influences the stabilization of the language of the two types of agents differently: the lower the proportion of biased agents, the bigger the difference in stabilization time between the biased and the unbiased agents. That is to say, when only a small proportion of the population is biased, the language of these biased agents will need a long time to stabilize, but when half of the population is biased, unbiased and biased agents will reach stability at approximately the same time. In scale-free and small-world networks, this difference is positively affected by network size, and is higher for scale-free networks.

Thus, stabilization time varies widely depending on network type and size, and the strength and frequency of the bias. In all three types of networks, the language stabilizes at roughly the same time when the networks are small, but only in random networks the language stabilizes faster as network size increases. Overall, agents in scale-free networks tend to require more time to stabilize. When the agents are strongly biased, the difference in stabilization time between the biased and the unbiased agents is negatively influenced by the proportion of biased agents.

## 4. Discussion

We introduced here an agent-based model that quantitatively investigates the dynamics of amplification and expression, to the level of the population's language, of linguistic variants influenced by individual-level biases. While our study is by far not the first to investigate the influence of communicative structure on language transmission (Gong et al., 2004, 2012a) nor of the effects of biases on language change and evolution (e.g., Kirby and Hurford, 2002; Kirby et al., 2007), we are the first (to our knowledge) to combine the two in a non-trivial way, by allowing agents with intrinsically different biases to interact through a structured communicative network. We show that, contrary to the “intuitive view” that the biased minority ends up adopting the language of the unbiased majority, even weakly biased agents present in a small part of the population *can* affect the language of the whole population, when the communicative network of the population is structured. The reverse is also true, as biased agents are accommodating to the unbiased agents. Thus, the language value of the population reflects often mostly the initial language of the society carried by unbiased agents. The influence of the bias increases with the strength and the population frequency of the bias, but, unlike Navarro et al. (2018), we do not find here evidence for a disproportionately large influence of strongly biased agents. However, our results show that even weak and rare biases can exert a stronger influence than a priori expected, as the relationship between population language, bias strength and bias frequency is not linear. Maybe counter-intuitively, far from being “swamped” by the majority, weakly biased agents representing but a minority, can nevertheless disproportionately influence the language of the majority. With hindsight, these results may appear unsurprising given our use of a Bayesian model which, by definition, given enough data should move away from its prior and come to reflect the observed data. However, we have to point out that it is far from clear what “enough data” means, how the structured nature of the interactions affects this process, and that real languages might be far from a state of equilibrium (e.g., Cysouw, 2011)—therefore, even in this constrained context our results are arguably unexpected, showing that even weak and rare biases, implemented in a way that favors erasure by the incoming data, do survive in the emergent, community-wide behavior.

We tested here three hypotheses concerning the manner in which individual-level biases may influence the population's language. First, we investigated the way in which the biased and the unbiased agents interact and influence each other. Our findings match the prediction that the presence of biased agents has a significant effect on the language that emerges in the population, as their bias affects the language of the unbiased agents. More generally, all agents tend to converge, after interacting repeatedly, toward a compromise in their language somewhere between the initial language of the biased and the unbiased agents. Interestingly, while the network structure does not affect the final language at which the population stabilizes, it does affect the speed with which it stabilizes: this is faster in larger random networks, and generally slower in scale-free networks. This is consistent with Raviv et al. (2020)'s experimental findings, where it is suggested that stability is faster in denser networks, while sparser networks would be slower to stabilize. Differences in convergence times between different network structures was also found in the statistical physics literature that studies cultural dynamics (Baxter et al., 2008; Castellano et al., 2009; Blythe, 2015). In our simulations, the high connectivity in random networks led agents to receive many utterances from their neighbors at each iteration, while in scale-free networks, each agent heard, on average, less utterances at each iteration. However, in scale-free networks, it is important to note that the internal representation of the influencers evolves faster (i.e., become “narrower” around a specific value) than for poorly connected individuals.

The role of network structure is also highlighted by our second hypothesis: we expected that the biased agents would manage to retain a trace of their bias in their language even after interacting repeatedly with the unbiased agents. Strikingly, our findings match this expectation, but only in scale-free networks (and, to a smaller extent, also in small-world networks). In such networks, the biased agents stabilize on a slightly different language than the unbiased agents, making the two groups easily identifiable even after repeated interactions. Moreover, our results show that the presence of the bias among the top influencers in the network (agents with the highest network centrality) results in the amplification of these inter-individual differences (especially through the creation of an “elite” community with a different language), but, importantly, does not have a strong effect on the final language of the whole population (except for very small scale-free networks with 10% of strongly biased agents). Thus, communication does not necessarily enforce uniformity among the agents, but instead inter-individual variation persists even after repeated interactions in structured networks. But then, how do these types of networks match the reality of human linguistic interactions? While a consensus has not yet been reached (Ke et al., 2008), most authors (Xiao Fan Wang and Guanrong Chen, 2003; Kaiser and Hilgetag, 2004) suggest that a realistic model should incorporate features of both scale-free and small-world networks, and that random networks are definitely out. As such, our own results can be taken to support these suggestions: indeed (as discussed in section 1), there is widespread inter-individual variation in language that persists into adulthood, but our simulated random networks lost all traces of inter-individual variation (see Heterogeneity *intra* group in the Supplementary Materials).

The third hypothesis further explores the idea that inter-individual variation may lead to the emergence of linguistic communities using different languages. Our results show, indeed, that even without any inter-individual differences in the beginning, as long as the initial bias is too strong, the structure of scale-free and small-world networks leads to the emergence of communities differing in their languages. This is broadly in line with fundamental sociolinguistic theory and data showing that multi-level structured linguistic variation within linguistic communities is the norm (Labov, 1975; Milroy and Gordon, 2008; Meyerhoff, 2015). Our study addresses these issues in a novel way, by explicitly modeling both inter-individual variation and structured linguistic interaction. We found that adding biased agents (and especially strongly biased agents) randomly in the scale-free and small-world networks amplify the linguistic variation between the communities, but how does such inter-individual variation influence the emergence of such communities? We suggest that randomly placing biased agents within a network may lead to the presence of several biased agents within the same structural community (i.e., a community due to the connectivity structure of the network), while some other structural communities may end up without any biased agents. Therefore, communities with many biased members will tend to differ in the use of the variant affected by the bias from the communities without any biased members. However, in reality the biases may not always be randomly distributed in the population, but instead have a patterned distribution (due to a combination of geographic, historical, and demographic factors), as found for biases rooted in human genetics (Dediu and Ladd, 2007; Wong et al., 2020) or the vocal tract (Dediu et al., 2017; Blasi et al., 2019; Dediu and Moisik, 2019), feeding precisely into this amplification and differentiation process.

Interestingly, our results also contribute to the debate concerning the differences between modeling the linguistic agents as Bayesian samplers (SAM) or maximizers (MAP). Early influential studies of simple transmission chains (Griffiths and Kalish, 2007; Kirby et al., 2007) found that SAM and MAP differ fundamentally in their asymptotic behavior, in that SAM always converge to their prior distribution, while MAP's behavior is more complex (including the amplification of weak biases). However, these simple results don't generalize in more complex settings (Dediu, 2009; Ferdinand and Zuidema, 2009; Smith, 2009; Perfors and Navarro, 2014), and our results are in line with these findings: allowing the interactions between agents to be structured by non-random networks fundamentally alters the way language emerges in populations of SAM and MAP agents and may even erase the alleged differences between them.

Most studies of language change suggest that the replacement of one variant by another tends to follow an “S”-shaped (or sigmoid) curve (Ke et al., 2008; Blythe and Croft, 2012), where the new variant starts as very rare, increases in frequency initially slowly, then very rapidly, then slows down again, until the total replacement of the old variant. However, our simulations do not show such results because our agents have no mechanism that forces them to pick one variant over the other, their choices being instead probabilistic. Thus, it is very unlikely that one variant will completely replace the other in their languages, but, in future work, if such a behavior is deemed necessary, we could easily implement such a selection mechanism.

Despite its novelty, the work presented here suffers from several limitations that may impact its generalizability and realism. First, we use a Bayesian approach to model language acquisition and production: while this has a respectable pedigree both in the cognitive sciences in general and in studying language evolution and change in particular, it is also heavily debated to what degree the Bayesian paradigm reflects reality (e.g., Kirby et al., 2007; Griffiths et al., 2008; Dediu, 2009; Ferdinand and Zuidema, 2009; Perfors, 2012; Hahn, 2014). Our choice here was rather pragmatic, in the sense that our Bayesian models are very simple mathematically, computationally fast, flexible enough, and arguably realistic enough *given the aims of our study*: “[a]ll models are wrong, but some are useful” and here, a Bayesian agent usefully abstracts away from the enormous (and only partially understood) complexity of language acquisition and production but still captures the fact the linguistic behavior of one's community affects one's own representation of language, as well as the many factors affecting one's use of language. Importantly, we do believe that the main *qualitative* findings of our study do not critically depend on the use of Bayesian models, but this is, of course, an empirical question to be answered by future studies where only the agent model is changed, keeping everything else the same. Moreover, this is but a first step in a longer research programme and we do consider a variety of models, Bayesian and not (see for some examples of such non-Bayesian models in our own work, Dediu, 2008), as appropriate, given the parameters of interest in each study. Second, we only modeled at most two discrete types of agents co-existing in a population (biased and unbiased), but the reality is much more complex and continuous; while this shortcoming can be addressed by allowing more types of agents (or a continuous distribution of agents) in a network, it greatly complexifies the experimental design and the analysis of the results. Third, the structure of our networks is rather artificial and is fixed in time; while the first issues can be addressed through the use of real-world data (e.g., sociolinguistic case studies or data derived from social media such as Facebook and Twitter), the second is more complex to implement, as it requires not only the change in network topology and connection strength, but also the removal of agents (death or emigration) and the introduction (birth or immigration) of new agents (naive or with a pre-existing language), and the move to a multi-generation paradigm. However, the fixity of our network structure might affect our results, as it is expected that the linguistic interactions themselves alter the topology and strength of the connections, creating thus complex feedback loops between the evolution of the network and of the language. Fourth, our results must be critically combined with real-world data derived from observational (Abitbol et al., 2018) and experimental studies (Raviv, 2020), in order to refine the model but also to inform future real-world experimental design, data collection and analysis.

In conclusion, our results—while preliminary—show that inter-individual variation, especially when structured by communicative networks, does affect language, and may even be one of the drivers behind the emergence of linguistic diversity and complexity. They also highlight that, when discussing the influence of biases on language change and diversity, inquiring only about the effects of bias strength and frequency in the population misses the essential role played by the fact that there is structure to human interactions, and that rarely two linguistic exchanges are mirror images of each other. Combined with other types of evidence, with the ubiquity of inter-individual variation, and the quintessentially structured nature of human interactions, this suggests that we must focus our attention on this rather neglected factor in the origins and evolution of the bewildering patterns of linguistic diversity still visible around the world.

## Data Availability Statement

The datasets presented in this study can be found in online repositories. The names of the repository/repositories and accession number(s) can be found in the article/Supplementary Material.

## Author Contributions

DD, MJ, MA-T, and FP designed the research. MJ and MA-T performed the research. MJ wrote and ran the simulations, and performed data analysis and plotting. DD, MJ, and MA-T drafted the manuscript. DD and MJ acquired funding. All authors read, contributed to, and approved the manuscript.

## Conflict of Interest

The authors declare that the research was conducted in the absence of any commercial or financial relationships that could be construed as a potential conflict of interest.

## Footnotes

^1^In our simulations, the initial values of α and β are always higher than 1.

^2^We slightly modified the Erdos-Renyi algorithm, in order to study networks without sub-graphs and/or isolated nodes (by randomly adding a link to isolated nodes). This can change the overall connectivity of the graph in very small networks (see Supplementary Materials).

^3^Note that the relevance of using Louvain algorithm to extract communities in random networks is debatable.
