# Supplementary material for: Interindividual Variation Refuses to Go Away: A Bayesian Computer Model of Language Change in Communicative Networks
Source: Front Psychol. 2021 Jun 21;12:626118. doi: 10.3389/fpsyg.2021.626118 (PMC8257003; doi:10.3389/fpsyg.2021.626118)
Supplement: Supplementary file 1 [file Data_Sheet_1.ZIP › Supplementary_materials_ABM.html]

 

 

 

 
 
 


 


 Interindividual variation refuses to go away: a Bayesian computer model of language change in communicative networks 

 
 
 
 
 
 
 
 
 
 
 
 

 

 
 
 


 


 


 

 

 


 


 

 


 


 
 
 
 
 
 

 


 


 Interindividual variation refuses to go away: a Bayesian computer model of language change in communicative networks 
 Full statistical analysis and plots 
 Mathilde Josserand, Marc Tang, François Pellegrino and Dan Dediu 
 Sun May 9 18:03:33 2021 

 


 
 Introduction 
 
 Dataset 
 We ran simulations with Netlogo model (see Github  mathjoss/bayes-in-network ), using different combination of parameters (structure of the network, percentage of biased people, shape of the bias…) and measuring multiple variables related to the language value of the population. 
 The results are stored in 3 different files : 
 
 
  
  
  
  
  
  
 
 
 
 Input file 
 Format 
 DV 
 IV 
 Number of replications 
 Maximum number of ticks 
 
 
 
 
  example_time.csv  
 CSV file 
 language value (all agents, biased agents, unbiased agents) 
 Scale-free, 500 agents, SAM, 10% biased agents, initial language 
 100 
 5000 
 
 
  analysis.csv  
 CSV file 
 - language value (all agents, biased agents, unbiased agents)   - stabilisation time (all agents, biased agents, unbiased agents)   - communities (mean and std of the language value + number of agents in each community) 
 set of combination  1  (see  Set of combination 1 - analysis.csv ) 
 100 
 5000 
 
 
  extra_analysis.csv  
 CSV file 
 language value (all agents) 
 set of combination  2  (see  Set of combination 2 - extra_analysis.csv ) 
 50 
 500 
 
 
  synchr_versus_asynchr.csv  
 CSV file 
 language value (all agents) 
 Set of combination  3  (see  Set of combination 3 - synchr_versus_asynchr.csv ) 
 100 
 5000 
 
 
  stabilization.csv  
 CSV file 
 stabilization time for biased and unbiased agents  (rounded by 10,000 or 100,000)  
 Only one condition   (scalefree, 500 SAM agents, 10% strong bias, 10% influencers, initial lang) 
 100 
 5000 
 
 
 
 
 
 Framework - methods 
 Our framework is implemented in NetLogo 6.1.1 ( https://ccl.northwestern.edu/netlogo/ ), the experiments were run on an Intel Core i7-8700, 32Gb RAM system under Ubuntu 18.04, and the results analysed using R 3.6.3/Rstudio 1.4 on machines running Ubuntu 18.04 and macOS 10.15 (Catalina); the full source code and results are available at Github ( mathjoss/bayes-in-network ). 
 Our simulation framework is based on previously published models  (Dediu, 2009) ,  (Dediu, 2008)  and has three main components: the language, the agents, and the communicative network. The language is modelled here as being composed of one (or more) binary features, that are obligatorily expressed in each individual utterance produced or perceived by the agents. We may think of these abstract features as representing, for instance, the use of the alveolar trill /r/ (value 1) or of a different r-like sound (value 0), the use of pitch to make a linguistic distinction (1) or not (0), having a subject-verb word order (1) or a verb-subject order (0), making a gender distinction (1) or not (0), using center embedding (1) or not (0), or any other number of such alternatives. Thus, if we take the /r/ interpretation, a set of utterances {1,1,1} might be produced by an agent that can trill without issues, a {0,0,0} by one that cannot, and {1,0,1} by an agent that either does not make the distinction or whose ability to trill is affected by other factors (e.g., socio-linguistic or co-articulatory). Each agent embodies three components: language acquisition, the internal representation of language, and the production of utterances. The first concerns the way observed data (in the form of “heard” utterances) affect (or not) the internal representation of language that the agent has. The second is the manner in which the agent maintains the information about language. And the third, the way the agent uses its internal representation of the language to produce actual utterances. 
 We opted here for a Bayesian model of language evolution as introduced by  (Griffiths & Kalish, 2007) , and widely used in recent studies of language evolution and change (e.g.  (Dediu, 2009) ,  (Dediu, 2008) ,  (Kirby, Dowman, & Griffiths, 2007) , among others). To do so, we used agent-based modeling in Netlogo, where we created societies of agents sharing connections with each other. Netlogo programs is available on Github  mathjoss/bayes-in-network  and contains a lot of functionalities not used in this analysis. To understand how to use our Netlogo code and parameters, please refer to  Appendix: Netlogo guide . 
 As a general approach, it proposes that there is a universe of possible languages (discrete or continuous),  \(h \in U,\)  and that an agent maintains at all times a probability distribution over all these possible languages. Initially, before seeing any linguistic data, the agent has a prior distribution over these possible languages,  \(p(h)\) , and, as new data (in the form of observed utterances),  \(d = \{u_{1}, u_{2}, … u_{n}\}\) , come in, this probability is updated following Bayes’ rule, resulting in the posterior distribution:  \[p(h|d) = \frac{p(d|h).p(h)}{p(d)}\]  that reflects the new representation that the agent has of the probability of each possible language  \(h \in U\)  after having heard the utterances composing the data  \(d\) . In this,  \(p(d|h)\)  is the likelihood that the observed data  \(d\)  was generated by language  \(h\) , and  \(p(d)\)  is a normalisation factor ensuring that  \(p(h|d)\)  is a probability bounded by 0.0 and 1.0. 
 In this paper, we model a single binary feature and consequently the utterances,  \(u\) , collapse to a single bit of information, “0” or “1”. The observed data,  \(d\) , become binary strings, and one of the simplest models of language is that of throwing a (potentially unfair) coin that returns, with probability  \(h \in [0,1]\) , a “1” (otherwise, with probability  \(1-h\) , a “0”). Thus, the universe of our languages,  \(h\) , is the real number interval  \(U = [0,1] \subset {\rm I\!R}\) , and the likelihood of observing an utterance  \(u \in \{ 0, 1 \}\)  is given by the Bernoulli distribution with parameter  \(h\) ; for a set of utterances  \(d = \{u_{1}, u_{2}, … u_{n}\}\) , the likelihood is given by the  binomial distribution  with parameters  \(k = |\{u_{i}=1\}_{i=1..n}|\)  (the number of utterances “1”),  \(n\)  (the total number of utterances), and  \(h: p(d|h) = Binomial(k,n,h) = \frac{n!}{k!(n-k)!}h^{k}(1-h)^{n-k}\) , where  \(x! = 1 \cdot 2 \cdot ... \cdot (x-1) \cdot x\) ; thus, we can reduce the set of utterances forming the data  \(d\) , without any loss of information, to the number of “1” utterances ( \(k\) ) and the total number of utterances ( \(n\) ). In Bayesian inference we sometimes use the conjugate prior of a given likelihood, in this case, the Beta distribution defined by two shape parameters,  \(\alpha\)  and  \(\beta\) , with probability density  \(f(x,\alpha,\beta) = \frac{1}{B(\alpha,\beta)}x^{\alpha-1}(1-x)^{\beta-1}\) , where  \(B(\alpha,\beta)\)  normalizes the density between 0.0 and 1.0. With these, the prior distribution of language  \(h\)  is  \(f(h,\alpha_{0},\beta_{0})\) , with parameters  \(\alpha_{0}\)  and  \(\beta_{0}\)  defining the shape of this distribution (see below), and the posterior distribution, updated after seeing the data  \(d=(k,n)\) , is  \(p(h|d) = f(h,\alpha_{1},\beta_{1})\) , where  \(\alpha_{1} = \alpha_{0} + k\)  and  \(\beta_{1} = \beta_{0} + (n-k)\) ; thus, the posterior distribution is also distributed Beta, with the shape parameter  \(\alpha\)  “keeping track” of the “1” utterances, and  \(\beta\)  of the “0” utterances, and the Bayesian updating is reduced to simple (and very fast) arithmetic operations. When it comes to utterance production, a SAM agent chooses a value  \(h \in [0,1]\)  from the  \(B(\alpha_{1},\beta_{1})\)  distribution (i.e., proportional to  \(f(h,\alpha_{1},\beta_{1})\) )), while a MAP picks the mode of the distribution,  \(h_{M} = \frac{\alpha_{1}-1}{\alpha_{1}+\beta_{1}-2}\) ; afterward, the agent uses this number between 0.0 and 1.0 as the parameter of a Bernoulli distribution (a coin throw) to extract a single “0” or “1” value with this probability – this value then is the utterance that the agent produces. 
 This choice (Bernoulli/Beta) not necessarily reflects how data is used by real humans in learning a language, but it has several major advantages, most notably its simplicity, transparency, and computational efficiency making it possible to run very large simulations on a consumer-grade computer in reasonable time  (Dediu, 2009) . Probably the most relevant here concerns the fact that the bias can be modeled only through the shape parameters of the prior Beta distribution,  \(\alpha_{0}\)  and  \(\beta_{0}\) , as the likelihood function is fixed to the Binomial, and the utterance produce offers only a limited choice between SAM and MAP. However, the Beta distribution is notoriously flexible, and can be used to represent from (almost) flat (or uninformative) distributions, to extremely peaked and to “U”-shaped ones. Moreover, for unimodal cases, we can model not only the  location  of the peak (i.e., the “preferred” value), but also the  spread  of this peak (i.e., how “strong” is this preference, operationally, how much data is needed to change the preferred value); we actually describe the Beta distribution using these alternative parameters, the mode  \(\mu\)  (describing the “preferred location”) and the “spread”  \(\lambda\) , which are linked to the shape parameters  \(\alpha\)  and  \(\beta\)  (see Box 1). Thus, arguably, the Beta distribution is flexible enough to model relatively well an intuitive view of how such a bias might look like – not just a preferred value but also a strength of this preference. See Figure 1 for an example of how different prior distributions are updated upon seeing some data. 
 You can see more details about this process in  Strength and location of the bias . 
 
 
 Independent variables 
 In our Netlogo model, we used the following variables: 
 
 
  
  
  
  
 
 
 
 Parameters 
 Variable name 
 Dependencies 
 Comments 
 
 
 
 
 Network size 
  size_net  
 none 
 The number of agents (i.e., agents); it is fixed for a given run 
 
 
 Frequency of biased agents 
  prop_biased  
 none 
 The proportion of agents in the network that are biased; please note that here we consider networks containing a single type of biased agents 
 
 
 Bias location and strength 
  bias_strength  
 none 
 Only the strength value of biased agents varies ; the value for unbiased agent is fixed and set to  \(\mu_{0} = 0.5\) ,  \(\lambda_{0} = 0.9\) . See more information in  Strength and location of the bias  
 
 
 Proportion of highest centrality agents that are biased 
  influencers_biased  
 depends on  prop_biased  
 More information in  Influencers biased  
 
 
 Utterance production mechanism 
  learners  
 none 
 More information in  Learners  
 
 
 Network type 
  network  
 none 
 More information in  Network type  
 
 
 Initial language 
  init_langval  
 none 
 The total number of utterances (n0) and the number of utterances “1” (k0) presented to all the agents in the network in the initial iteration i = 0. More information in  Initial language  
 
 
 
 
 Set of combination 1 - analysis.csv 
 The parameters we used in the set of combination  1  are the following: 
 
 
  
  
 
 
 
 Variable 
 Values 
 
 
 
 
  size_net  
 10 (“tiny”)   50 (“small”)   150 (“medium”)   500 (“large”)   1000 (“very large”) 
 
 
  prop_biased  
 0% (“fully unbiased”)   10%   30%   50%   100% (“fully biased”) 
 
 
  bias_strength  
  \(\mu_{0} = 0.1,\)   \(\lambda_{0} = 0\)  (“biased fixed”)    \(\mu_{0} = 0.1,\)   \(\lambda_{0} = 0.6\)  (“biased flexible”)    \(\mu_{0} = 0.1\) ,  \(\lambda_{0} = 0.1\)  (“biased rigid”) 
 
 
  influencers_biased  
 0% (“Random”)   10% (“biased influences”) 
 
 
  learners  
 SAM (“sampler”)   MAP (“a posteriori maximizer”) 
 
 
  network  
 Random   Scale-free   Small-world 
 
 
  init_langval  
 k0 = 0, n0 = 0 (“no initial language”)   k0 = 4, n0 = 4 (“initial language”) 
 
 
 
 
 
 Set of combination 2 - extra_analysis.csv 
 The parameters we used in the set of combination  2  are the following: 
 
 
  
  
 
 
 
 Variable 
 Values 
 
 
 
 
  size_net  
 150 (“medium”) 
 
 
  prop_biased  
 0 to 100%,  in steps of 1%  
 
 
  bias_strength  
  \(\mu_{0}\)  = 0.1 (biased),  \(\lambda_{0}\)  = 0.01 to 0.99,  in steps of 0.01  
 
 
  influencers_biased  
 0% (“Random”)   50% (“biased influences”)   100% (“biased extremely influent”) 
 
 
  learners  
 SAM (“sampler”) 
 
 
  network  
 Random   Scale-free   Small-world 
 
 
  init_langval  
 k0 = 4, n0 = 4 (“initial language”) 
 
 
 
 
 
 Set of combination 3 - synchr_versus_asynchr.csv 
 The parameters we used in the set of combination  3  are the following: 
 
 
  
  
 
 
 
 Variable 
 Values 
 
 
 
 
  size_net  
 10 (“tiny”)   50 (“small”)   150 (“medium”)   500 (“large”) 
 
 
  prop_biased  
 0% (“fully unbiased”)   10%   30%   50%   100% (“fully biased”) 
 
 
  bias_strength  
  \(\mu_{0} = 0.1,\)   \(\lambda_{0} = 0.6\)  (“biased flexible”)    \(\mu_{0} = 0.1\) ,  \(\lambda_{0} = 0.1\)  (“biased rigid”) 
 
 
  influencers_biased  
 0% (“Random”) 
 
 
  learners  
 SAM (“sampler”) 
 
 
  network  
 Random   Scale-free 
 
 
  init_langval  
 k0 = 4, n0 = 4 (“initial language”) 
 
 
   Condition   
  Asynchronous     Synchronous  
 
 
 
 
 
 Strength and location of the bias 
 The first  internal representation of the language  (at  \(t = 0\) ) is represented by a Beta distribution ( alpha ,  beta ). However, the Beta distribution is notoriously flexible, and can be used to represent from (almost) flat (or uninformative) distributions, to extremely peaked and to “U”-shaped ones. For unimodal cases, we can model: 
 
  \(\mu_{0}\) : location of the bias (or mode), i.e., the “preferred” value. The higher, the more likely the individual will produce utterances = 1 
  \(\lambda_{0}\) : the spread of the peak (or strength of the bias), i.e., how “strong” is this preference, operationally, how much data is needed to change the preferred value. The higher, the less strongly biased. 
 
 We actually describe the Beta distribution using these alternative parameters, the mode  \(\mu_{0}\)  and the “spread”  \(\lambda_{0}\) , which are linked to the shape parameters ( alpha ,  beta ) using a small algorithm: 
 
 the user can select the mode of the Beta distribution (= the location of the bias), and the learning acceptance (= how strong the bias is); 
 the program computes the lower and upper uncertainty limits from the given mode and learning acceptance, such that these limits are within the interval [0, 1]; 
 the values for the mode and the upper and lower limits are passed to the  betaExpert  from from the  prevalence  package, which computes the unique values of  alpha  and  beta ; for optimization and future-proofing reasons, we precomputed and hard-coded the  alpha  and  beta  values used in this paper within our NetLogo script (available in the GitHub repository  mathjoss/bayes-in-network ). 
 
 (see hidden code below for more information) 
 In order to run faster simulation, and to prevent compatibility problems in Netlogo, we saved these alpha and beta values directly inside the Netlogo code. For example, this algorithm creates the following  alpha  and  beta  values according to  \(\mu_{0}\)  and  \(\lambda_{0}\) : 
 
 
 
 ( \(\mu_{0}\) ,  \(\lambda_{0}\) ) 
 (alpha, beta) 
 Parameter 
 
 
 
 
 (0.1, 0) 
 ( \(3*10^{8}\) ,  \(27*10^{8}\) ) 
 (“biased fixed”) 
 
 
 (0.1, 0.1) 
 (10.96, 90.62) 
 (“biased rigid”) 
 
 
 (0.1, 0.6) 
 (1.58, 6.19) 
 (“biased flexible”) 
 
 
 (0.5, 0.9) 
 (2.2, 2.2) 
 (“unbiased”) 
 
 
 
They can be visually represented like this:
 
 
 
 Figure 1.  Visualization of Beta distributions.
 
 
 For the “bias fixed”, we set a Bayesian curve so thin and high that it won’t change, even after a huge number of interactions. Then, for plotting purpose, we did not draw it on this plot, but on a separate plot (see below). 
 
 
 
 Figure 2.  Visualization of Beta distribution for the fixed bias. Please note that the y axis is completely different.
 
 
Here is a zoom on the curve:
 
 
 
 Figure 3.  Visualization of Beta distributions for the fixed bias, zooming on the curve.
 
 
 Hearing utterances will gradually change the internal representation of the language for each agent, whatever their starting distribution. As the fixed bias is not changing with time, we did not plot it in the following plots. 
 For example, after hearing 10 and 20 utterances = 1, the internal representation of the language will be like: 
 
 
 
 Figure 5.  The evolution of some examples of Beta priors (thick solid curves) after seeing some data (utterances), to become successive Beta posterior distributions (thin curves). Blue: an individual strongly biased against the feature; red: an individual weakly biased against the feature; and black: an unbiased individual. Top row: the prior distributions before seeing any data (“at birth”)’ middle row: the Beta distributions updated after seeing n=10 utterances all containing the value “1”; bottom row: after seeing n=20 such utterances.
 
 
 
 
 Initial language 
 The initial language parameter corresponds to two situations: 
 
  on the one hand, it can model the (quite unrealistic) case where agents are born in a society without any pre-existing language or where they are not exposed to any linguistic input (k0 = 0, n0 = 0), so that the agents must create their first utterances based only on their prior bias ( init_langval = 0  or  init_langval = no  in some plots).  
  on the other hand, it can model the more common case where agents are born in a society with a pre-existing language already biased towards the use of the feature (k0 = 4, n0 = 4); this is modelled by presenting all the agents with the same 4 utterances “1” in the initial iteration, so that the first utterances generated by the agents are based both on on their prior bias and the linguistic input from the society. In this analysis, the variant supported by agents having a bias (both strong or weak) is always the utterance “0” ( init_langval = 4  or  init_langval = yes  in some plots).  
 
 Here is a visualization of the Beta distribution curve of biased and unbiased agents, for both conditions of the initial language of the society: 
 
 
 
 Figure 6.  With or without an initial language: these show the Beta distributions of the agents in the case where no initial language exists in the society (bottom row) and when such an initial language (mildly biased toward “1”) does exist (top row). The colors of the curves represent the three types of agents in our simulation (unbiased, and weakly and strongly biased; see also Figure 1).
 
 
 As the condition with an initial language value of the society is more realistic, we will use this one preferentially in the computations. 
 Why did we choose 4 for the number of initial utterances = 1 heard by the agents? 
 This value of 4 is arbitrary, and it could have been another value indeed. Nevertheless, the value was chosen in order to generate an ‘interesting’ distribution of bias. The weak bias became biased in the middle, while the strong bias is still on the side of utterance=0. We generated plots with the evolution of the Beta priors according to the number of utterances = 1 given, and we used this plot to select this value (see below). 
 
 
 
 Figure 7.  Analysis used to find the number of initial utterances heard by the agents. The vertical panels shows the number of utterances, and we can see the posterior distribution of the Beta distribution for biased and unbiased agents after hearing these initial utterances.
 
 
 
 
 Learners 
 There are two widely-used strategies to produce utterances (among, the many possible ones;  (Kirby et al., 2007) ): 
 
   sampler strategy (SAM) : a language h can be sampled at Random from the universe of possible languages proportional to its probability in the posterior distribution  \(p(h|d)\) .  
   maximum a posteriori strategy (MAP) : we can pick the language  \(h_M\)  that has the maximum posterior probability  \(max_{h \epsilon U}(p(h|d))\)   
 
 
 
 Time 
 It is possible to use two types of networks: 
 
   synchronous : the language values of all agents are updated simultaneously at the end of each iteration, after all agents have talked once. More precisely, in a given iteration, each agent is selected in turn in a random order and is allowed to produce one utterance (“speak”), utterance which is “heard” by all its network neighbours. However, the agents do not update their internal representation of language until all have “spoken” (i.e., at the end of the iteration). In this way, each agent has the chance to “speak” and it does so using its representation of language from the previous iteration (if any), unaffected by any utterance they might have “heard” during the current iteration.  
   asynchronous : In a given iteration, each agent is selected in turn in a random order (random permutation) and is allowed to produce one utterance (“speak”), utterance which is “heard” by all its network neighbors. The language value of “listeners” is updated immediately after the they hear the speaker’s utterance. This is more similar to real-world situation, and also computationally less costly.  
 
 In this paper, we used an  asynchronous  network. However, an option is available on  Netlogo  to switch to  synchronous  network. We ran a set of simulations in order to observe the potential difference, and we did not find any: 
 
 
 
 Figure 8.  Asynchronous (blue) versus synchronous (green) network: these compare the final value of language of the population in different types of network. We did not plot all possible sets of combinations, but only Random and Scale-free networks, with Bayesian agents, 10% of influencers and an initial value of the language. Please note that there are no difference in other set of conditions; this set has been selected for plotting purposes.
 
 
 The aim of the plot above is only to compare the difference between the language values in a synchronous versus asynchronous network, in different sets of conditions: an analysis of the result will be possible in the following parts. 
 Each round, each individual says one utterance, and listen to the utterance(s) of his neighbor(s). 
 In this analysis, we call each round a  tick . 
 We study the evolution of language on a period of time containing  5000  ticks for the main analysis, and  500  ticks for the systematic bias effect study. 
 
 
 Network type 
 The network represents the socio-linguistic structure of a community, and constrains the linguistic interactions between agents. The agents are the network’s agents, and if there is an edge between two agents then those two agents will engage in linguistic interactions. 
 Please note that we consider here only static networks: there is no change, during a run, in the number of agents, the properties of the agents (bias and utterance production mechanism and in the topology of the network (i.e., the pattern of edges connecting the agents). 
 Likewise, our model does not include directed nor weighted edges (i.e., the two connected agents can interact symmetrically, and there is no way to specify that two agents might interact “more” than others), but we do think that dynamic weighted directed networks are an important avenue to explore in the future. 
 In this analysis, we use 3 different types of networks, always generated randomly in Netlogo:  Scale-free ,  Random , and  Small-world  networks. 
 
 Scale-free networks 
 
 Algorithm 
 We use the preferential attachment algorithm  (Barabási, Albert, & Jeong, 2000) . It starts from a seed of agents and gradually adds new ones; new links are created between the newly-added agents and the pre-existing agents following the rule that the more a agent is connected, the greater its chance to receive new connections. Formally, the probability  \(p_i\)  that a new agent is connected to agent  \(i\)  is  \(p_i= \frac{k_i}{\sum_{j}k_j}\) , where  \(k_i\)  is the degree of agent  \(i\) , and the sum is over all pre-existing agents  \(j\) . 
 
 
 Characteristics 
 Scale-free networks exhibit a  power law degree distribution : very few agents have a lot of connections, while a lot have a limited number of links. These type of networks are found, for example, on the Internet  (Albert, Jeong, & Barabási, 1999)  or in cell biology  (Albert, 2005) . 
 Let’s observe the degree distribution of our generated scale-free networks: 
 
 
 
 Figure 9.  Degree distribution for random networks, for different sizes of networks. Please note that the x and y axis are different according to the network size.
 
 
 … And their connection probability: 
 
 
 
 Figure 10.  The connection probability for random networks, for different sizes of networks.
 
 
 
 
 
 Small-world networks 
 
 Algorithm 
 We use a classic  beta model  of the Watts-Strogatz algorithm  (Watts & Strogatz, 1998) . The algorithm first creates a ring of agents, where each agent is connected to a number  \(N\)  of neighbours on either side, and then rewired with a chosen probability  \(p\) . 
 In this model, we always use the value  \(N = 4\)  and  \(p=0.1\) . 
 
 
 Characteristics 
 This process leads to the creation of hubs and the emergence of  short average path lengths . Small-world properties were popularized by  (Milgram, 1967) ’s “Six degrees of separation” idea, and are found in many real-world phenomena, such as social influence networks  (Kitsak et al., 2010)  and semantic networks  (Kenett et al., 2018) . 
 We saved the degrees of each node inside a column, in order to observe the distribution among all replications. 
 Let’s observe the degree distribution of our generated small-world networks: 
 
 
 
 Figure 11.  Degree distribution for small-world networks, for different sizes of networks. Please note that the x and y axis are different according to the network size.
 
 
 … And their connection probability: 
 
 
 
 Figure 12.  The connection probability for small-world networks, for different sizes of networks. Please note that the x-axis and y-axis are different compared to random and scale-free networks. Please note that the x scale is different than for scale-free and random networks.
 
 
 
 
 
 Random networks 
 
 Algorithm 
 We use Erdos & Renyi popular algorithm  (Erdős & Rényi, 1959) . We specify the number of agents and the overall connectivity of the graph giving the probability of adding an edge between any two agents ( \(p\) ); in this model, we always use  \(p=0.1\) . 
 
 
 Characteristics 
 It is an unrealistic baseline model, which does not represent the structure of real-world networks. 
 Let’s observe the degree distribution of our generated random networks: 
 
 
 
 Figure 13.  Degree distribution for random networks, for different sizes of networks. Please note that the x and y axis are different according to the network size.
 
 
 … And their connection probability: 
 
 
 
 Figure 14.  The connection probability for random networks, for different sizes of networks. Please note that the x-axis and y-axis are different compared to scale-free and small-world networks.
 
 
 
 
 
 Visualization 
   
 
 
 
 Influencers biased 
 We are interested to know what happens if the most influential people in a network are biased. To investigate it, we created a variable  influencers_biased : it corresponds to the percentage of highest centrality agents that are biased. 
 As an example, if there are 20% of biased agents in the network, and 15% of influencers biased, it means that the 15% most influential agents will be biased, and 5% of the rest of the network will be randomly biased. Practically speaking: 
 
 if ( prop_biased )  \(\geq\)  ( influencers_biased ), the ( influencers_biased ) most popular agents are biased, the rest of biased agents being randomly chosen in the population 
 if ( prop_biased )  \(<\)  ( influencers_biased ), then ( prop_biased ) most popular agents are biased. 
 
 In the  main analysis , we selected only 2 values for  influencers_biased  : 
 
  0%  of influencers biased, so the biased agents in the population are selected randomly; 
  10%  of influencers biased, so the 10% most influential agents are biased (if  prop_biased  >= 10, otherwise the  prop_biased  most influential agents are biased) 
 
 In the  systematic bias effect study , we selected 3 values for  influencers_biased  : 
 
  0%  of influencers biased, so the biased agents in the population are selected randomly; 
  50%  of influencers biased, so the 50% most influential agents are biased (if  prop_biased  >= 50, otherwise the  prop_biased  most influential agents are biased) 
  100%  of influencers biased, so the 100% most influential agents are biased (if  prop_biased  = 100, otherwise the  prop_biased  most influential agents are biased) 
 
 Here, most influential agents are determined using measures for  eigenvector centrality  (see more  here ). 
 
 
 
 Dependent variables 
 We measured different variables using Netlogo BehaviorSpace tool. 
 Here is the exhaustive list of all variables that we used in this analysis: 
 
 Language value 
 The language value of an agent at a given moment varies between 0 and 1, and is the mode of the Beta distribution representing the internal belief of the agent concerning the distribution of the probability of utterances “1” in the language. Biased agents typically start with a lower la than the unbiased agents, thus favoring the variant “0”. We also define the language value of a given group of agents (for example, a community or the whole network) as the mean of the language values of all the agents in the group. We decided to focus on the language value observed after 1,000 iterations, because the language value was always stabilized after this period. 
  Inter-individual variation  across the agents in a given network is an important outcome: we found that most biased and unbiased agents have very similar behaviors within their respective groups, justifying the use of the mean language values of the biased ( langval_biased ) and the unbiased agents ( langval_control ). We also computed the mean language value of the whole population ( langval_all ): even if there may be variation between groups (the biased vs the unbiased agents) and between agents, this value is a global indicator of the average language used in the population. 
 To summarize, we use the 3 following variables: 
 
 mean language value of  all  agents ( langval_all ) at final tick 
 mean language value of  biased  agents ( langval_biased ) at final tick 
 mean language value of  unbiased  agents ( langval_control ) at final tick 
 
 These variables were recorded directly inside BehaviorSpace, Netlogo. 
 
 
 Difference between unbiased and biased agents 
 Here, we used the signed difference between the mean language values of the unbiased agents and the mean language values of biased agents, as this gives very similar results to the much more computationally expensive method of computing all pairwise differences between all unbiased and biased agents: 
 
 difference of mean language value of  unbiased  agents and mean language value of  biased  agents ( diff_group ) 
 
 We computed this variable from the previous language value means, in R. 
 
 
 Stabilization time 
 Intuitively, stabilisation time captures how long (in terms of interaction cycles) it takes for the language of a given network to reach a stable state. Given the inhomogeneous nature of the network, we consider two measures: the moment when the language value of the whole population stabilize ( stab_all ), the moment when the language value of the biased agents stabilizes ( stab_biased ) and the moment when the language value of the unbiased agents stabilizes ( stab_control ); these measures are estimated using the language values of their respective populations. To summarize, we use: 
 
 moment when the language value of the  society  stabilizes ( stab_all ) 
 moment when the language value of the  biased  agents stabilizes ( stab_biased ) 
 moment when the language value of the  unbiased  agents stabilizes ( stab_control ) 
 
 Please note that the measure  stab_all  is the less accurate and representative of the actual behaviour of agents. Consequently, we decided to mainly study the results of  stab_biased  and  stab_control . 
 These variables were computed using the mean language values above, on R. We used 2 different algorithms to compute the stabilization time ( method 1  and  method 2 ). After checking the results, we found  method 2  to be more accurate, so this Rmarkdown only shows the results of the analysis using  method 2 . 
 
 Method 1 
 We used a discrete sliding window in which we estimate the derivative (i.e., change) and we recorded this change. After the window slided along the whole period of time, we selected the 15 values closest to 0. The value we chose as the stabilization time was the  earliest  value among these 15 values. 
 This method is based on the method used by  (Jannsen, 2018)  (p. 79). Pratically speaking: 
 The maximum number of ticks of our model is  \(nIterations = 5000\) , and the size of the sliding window is  \(w = nIterations/10\) . We applied a  loess  function on the language values in each window, which is a local polynomial regression fitting (see more  here ). Then, we ran the following equation on the predicted point (regression line): 
  \[t(e_g) = \frac{e_{g+w} - e_g}{w}\]  
 and we obtain a sequence of elite fitness scores  \(\vec{e}= (t(e_1), t(e_2), ...)\) . The algorithm terminates at the end, so  \(length(\vec{e}) = nIterations - w\) . Then, we selected the 15 values closest to 0 in  \(\vec{e}\) . Among these values, we selected the value  \(t(e_g)\)  with the minimum  \(g\) . 
 
 
 Method 2 
 The estimation is based on the method developed in Jannsen (2018:79) and used a fixed-size sliding window within which we estimate the change in the language value, we multiply this number by 10,000, round it, and stop if this number is equal to zero (i.e., the slope is within  \(\pm\)  0.001 of 0.0) for 50 consecutive steps. Practically speaking, the maximum number of ticks of our model is  \(nIterations = 5,000\) , and the size of the sliding window is  \(\omega= nIterations/10\) . For a given window, we estimated the change,  \(t(e_{g})\)  using the following formula: 
  \[t(e_g) = \frac{e_{g+w} - e_g}{w}*10,000\]  
 On the rounded  \(t(e_{g})\)  values, we find the first value of  \(g\) ,  \(g_{stabilization}\) , when the rounded value of  \(t(e_{g})=0\) , and we stop if for 50 consecutive steps (i.e.,  \(g \in [g_{stabilization}.. (g_{stabilization}+50)]\) ), there is no change,  \(t(e_{g})=0\) ; in this case, the stabilization time is the first moment where there was no change, namely  \(g_{stabilization}\) . 
 Let us visualize where is the stabilization point found for an example: 
 
 
 
 Figure 15.  Stabilization times for the biased and the unbiased agents. This example uses a scale-free network with 500 agents, with SAM agents, where 10% of the top influencers are strongly biased, in the presence of an initial language.
 
 
 For this very same example, we can select a more  precise  way of computing the stabilization time (round by 100,000 instead of 10,000): 
 
 
 
 Figure 16.  Stabilization times for the biased and the unbiased agents. This example uses a scale-free network with 500 agents, with SAM agents, where 10% of the top influencers are strongly biased, in the presence of an initial language. Here, we selected a more precise way of computing stabilization time.
 
 
 We chose to select the first way of computing the stabilization point (round by  10,000 ). Indeed, even if for few examples (such as the one on the plot above) the language value of biased agents still evolves a little bit after the stabilization time, in a bigger number of examples (random, small world networks), it is more sensitive to the exact moment where the curve “looks” flat. 
 We also mention that changing the precision increases the stabilization time, but does not the conclusion on our results in  Stabilisation time . 
 
 
 
 Dissemination 
 First, the inter-replication variation is estimated by computing the standard deviation of the language values obtained among the R replications after 1,000 iterations. It captures the influence of various sources of Randomness on each particular run of a given condition, and we computed it for 3 different groups: 
 
 dissemination of the results of different replications for  all  agents ( diss_all ) 
 dissemination of the results of different replications for  biased  agents only ( diss_biased ) 
 dissemination of the results of different replications for  unbiased  agents agents ( diss_unbiased ) 
 
 These variables were computed using the mean language values above, on R. 
 The results are recorded inside a new table, which gather the values of dissemination for each combination of conditions:  data_dissemination . 
 
 
 Community detection 
 In order to study the possible differences in the language values of the agents belonging to different communities, we first detect the structural communities within the network using the  Louvain community detection  (see more info  here ) algorithm (as implemented in  NetLogo ’s  nw  extension package), which detects communities by maximizing modularity based on the connections agents share with each other, and not on the agents’ language values. 
   
 Since the network is static, we then use the detected communities to compute the language value of each community for each iteration: 
 
 mean language value of each community ( \(list mean\) ) ; 
 std language value of each community ( \(list std\) ) ; 
 number of agents in each community ( \(list nb node\) ). 
 
 The results are a list of data, the size depending on the number of communities detected by Louvain algorithm. Then, on R, we extracted 2 values from these 3 lists: 
 
  het_inter_5000 : heterogeneity  between  communities, computed with  \(sd(list mean)\)  
 
  Interpretation : A  low  number indicates that all communities have approximately the same language value, whereas a  high  number indicates that the communities inside the network have different language value. 
 
  het_intra_5000 : heterogeneity  within  communities, computed with  \(mean(list sd)\)  
 
  Interpretation : A  low  number indicates that people share the same language value inside each community, whereas a  high  number indicates that people can have pretty different language value inside each community. 
 
 
 
 Cleaning data 
 The computations used are available in  Netlogo ’s  BehaviorSpace  tool. Once we got the resulting  analysis.csv  file, we cleaned it and computed new values using the file  clean_compute.R  (available in Github). It: 
 
 changes variables’ name and keep only necessary columns 
 converts missing data to NaN (see  Missing data ) 
 computes the stabilization time for all, biased and unbiased agents using the language values for all ticks according the algorithm presented in  Stabilization time  
 reduces the dataset’s size by keeping only necessary information:
 
 keep only the language value and the communities value at tick 0, 1 and 5000 
 change the format so that they appear in different columns 
  
 creates a column for heterogeneity (between and within groups) using the communities mean and std 
 
 Consequently, the following variables are only recorded at tick 0, 1 and 5000 in the  analysis.csv  file: 
 
 Language values (for all, biased and unbiased agents) 
 Heterogeneity (inter and intra group) 
 
 
 
 Missing data 
 The following categories contain missing data: 
 
 When there are 0% of biased agents, the  language value  of biased agents, the  stabilization time  of biased agents, the  difference  between biased and unbiased agents are missing data. 
 When there are 0% of unbiased agents, the  language value  of unbiased agents, the  stabilization time  of unbiased agents, the  difference  between biased and unbiased agents are missing data. 
 In some very few cases (230 cases out of 180,000 cases), the Louvain algorithm detects only one community (for 10 agents networks). In this case, the value for heterogeneity inter group is missing data. 
 
 
 
 Summary 
 
 example_time.csv 
 Here is a summary of our dataset  example_time.csv : 
 Please note that in order to save some time to compute the replications, we computed the values for communities only for the tick 0, 1 and 5000. 
 As a quick reminder, this file only contains a subset of replications in order to show an example. It does  not  contain all the possible combinations of our independent variables. Go to  Dataset  for more information. 
 
 Table continues below 
 
  
  
  
  
  
 
 
 
 rep_id 
 prop_biased 
 bias_strength 
 influencers_biased 
 ticks 
 
 
 
 
 Min. :3701 
 10:3000600 
 0 :1000200 
 0 :1500300 
 Min. : 0 
 
 
 1st Qu.:3776 
 NA 
 0.1:1000200 
 10:1500300 
 1st Qu.:1250 
 
 
 Median :4750 
 NA 
 0.6:1000200 
 NA 
 Median :2500 
 
 
 Mean :4750 
 NA 
 NA 
 NA 
 Mean :2500 
 
 
 3rd Qu.:5725 
 NA 
 NA 
 NA 
 3rd Qu.:3750 
 
 
 Max. :5800 
 NA 
 NA 
 NA 
 Max. :5000 
 
 
 
 
 
  
  
  
 
 
 
 langval_all 
 langval_biased 
 langval_control 
 
 
 
 
 Min. :0.2679 
 Min. :0.0002 
 Min. :0.2976 
 
 
 1st Qu.:0.4790 
 1st Qu.:0.0004 
 1st Qu.:0.5322 
 
 
 Median :0.5967 
 Median :0.3761 
 Median :0.6184 
 
 
 Mean :0.5787 
 Mean :0.3470 
 Mean :0.6045 
 
 
 3rd Qu.:0.6906 
 3rd Qu.:0.6071 
 3rd Qu.:0.6987 
 
 
 Max. :0.7808 
 Max. :0.7368 
 Max. :0.8125 
 
 
 
 
 
 analysis.csv 
 Here is a summary of our dataset  analysis.csv : 
 Please note that the last number (in our dependent variables) indicates the  tick : for example,  langval_control_0  indicates the language value recorded at  tick 0  for unbiased agents, while  langval_control_5000  indicates the language value recorded at  tick 5000  for unbiased agents, etc. 
 It is the file that will be mainly used during our analysis. Go to  Dataset  and  Set of combination 1 - analysis.csv  for more information. 
 
 Table continues below 
 
  
  
  
  
  
 
 
 
 degrees 
 rep_id 
 prop_biased 
 learners 
 bias_strength 
 
 
 
 
 Length:180000 
 Min. : 1 
 0 :36000 
 MAP:90000 
 0 :60000 
 
 
 Class :character 
 1st Qu.: 3751 
 10 :36000 
 SAM:90000 
 0.1:60000 
 
 
 Mode :character 
 Median : 7500 
 30 :36000 
 NA 
 0.6:60000 
 
 
 NA 
 Mean : 7500 
 50 :36000 
 NA 
 NA 
 
 
 NA 
 3rd Qu.:11250 
 100:36000 
 NA 
 NA 
 
 
 NA 
 Max. :15000 
 NA 
 NA 
 NA 
 
 
 NA 
 NA 
 NA 
 NA 
 NA 
 
 
 
 
 Table continues below 
 
  
  
  
  
 
 
 
 size_net 
 init_langval 
 influencers_biased 
 network 
 
 
 
 
 10 :36000 
 0:90000 
 0 :90000 
 Random :60000 
 
 
 50 :36000 
 4:90000 
 10:90000 
 Scale-free :60000 
 
 
 150 :36000 
 NA 
 NA 
 Small-world:60000 
 
 
 500 :36000 
 NA 
 NA 
 NA 
 
 
 1000:36000 
 NA 
 NA 
 NA 
 
 
 NA 
 NA 
 NA 
 NA 
 
 
 NA 
 NA 
 NA 
 NA 
 
 
 
 
 Table continues below 
 
  
  
  
  
 
 
 
 het_inter_1 
 het_intra_1 
 langval_all_1 
 langval_control_1 
 
 
 
 
 Min. :0.00000 
 Min. :0.00000 
 Min. :0.0002 
 Min. :0.10 
 
 
 1st Qu.:0.01372 
 1st Qu.:0.05116 
 1st Qu.:0.2516 
 1st Qu.:0.43 
 
 
 Median :0.05215 
 Median :0.10924 
 Median :0.4325 
 Median :0.56 
 
 
 Mean :0.05956 
 Mean :0.11416 
 Mean :0.4315 
 Mean :0.57 
 
 
 3rd Qu.:0.09007 
 3rd Qu.:0.16018 
 3rd Qu.:0.6211 
 3rd Qu.:0.75 
 
 
 Max. :0.50297 
 Max. :0.49277 
 Max. :0.9241 
 Max. :0.92 
 
 
 NA’s :230 
 NA 
 NA 
 NA’s :36000 
 
 
 
 
 Table continues below 
 
  
  
  
  
 
 
 
 langval_biased_1 
 het_inter_5000 
 het_intra_5000 
 langval_all_5000 
 
 
 
 
 Min. :0.00 
 Min. :0.00000 
 Min. :0.000000 
 Min. :0.0000 
 
 
 1st Qu.:0.10 
 1st Qu.:0.00080 
 1st Qu.:0.001825 
 1st Qu.:0.1370 
 
 
 Median :0.12 
 Median :0.02193 
 Median :0.011200 
 Median :0.3452 
 
 
 Mean :0.19 
 Mean :0.03734 
 Mean :0.021448 
 Mean :0.3695 
 
 
 3rd Qu.:0.21 
 3rd Qu.:0.05710 
 3rd Qu.:0.032000 
 3rd Qu.:0.5546 
 
 
 Max. :0.86 
 Max. :0.44392 
 Max. :0.235132 
 Max. :0.9968 
 
 
 NA’s :36000 
 NA’s :230 
 NA 
 NA 
 
 
 
 
 Table continues below 
 
  
  
  
  
 
 
 
 langval_control_5000 
 langval_biased_5000 
 stab_all 
 stab_control 
 
 
 
 
 Min. :0.00 
 Min. :0.00 
 Min. : 2.0 
 Min. : 2.0 
 
 
 1st Qu.:0.24 
 1st Qu.:0.10 
 1st Qu.: 20.0 
 1st Qu.: 52.0 
 
 
 Median :0.42 
 Median :0.19 
 Median : 78.0 
 Median : 137.0 
 
 
 Mean :0.43 
 Mean :0.26 
 Mean : 130.3 
 Mean : 191.1 
 
 
 3rd Qu.:0.62 
 3rd Qu.:0.41 
 3rd Qu.: 198.0 
 3rd Qu.: 284.0 
 
 
 Max. :1.00 
 Max. :0.99 
 Max. :1110.0 
 Max. :1433.0 
 
 
 NA’s :36000 
 NA’s :36000 
 NA 
 NA’s :36000 
 
 
 
 
 
  
  
 
 
 
 stab_biased 
 diff_group 
 
 
 
 
 Min. : 2 
 Min. :-0.17 
 
 
 1st Qu.: 2 
 1st Qu.: 0.00 
 
 
 Median : 44 
 Median : 0.02 
 
 
 Mean : 116 
 Mean : 0.06 
 
 
 3rd Qu.: 183 
 3rd Qu.: 0.08 
 
 
 Max. :1498 
 Max. : 0.77 
 
 
 NA’s :36000 
 NA’s :72000 
 
 
 
 You can notice some NaN values in heterogeneity measures: this is when the network is very small (10 agents) and only one community has been detected by Louvain algorithm. As we will  not  study 10-agents network in the heterogeneity part, we can ignore these missing data. 
 
 
 extra_analysis.csv 
 Here is a summary of our dataset  extra_analysis.csv : 
 In order to save some time to compute the replications, we recorded here only the final language value for  all  agents at tick 500. 
 The size of the network is 150 agents, the learners are SAM sampler, and there is an initial value of the language of the society. Go to  Dataset  and  Set of combination 2 - extra_analysis.csv  for more information. 
 
 Table continues below 
 
  
  
  
  
 
 
 
 rep_id 
 prop_biased 
 bias_strength 
 influencers_biased 
 
 
 
 
 Min. : 1 
 Min. : 0 
 Min. :0.01 
 0 :1499850 
 
 
 1st Qu.: 281222 
 1st Qu.: 25 
 1st Qu.:0.25 
 50 :1499850 
 
 
 Median : 649936 
 Median : 50 
 Median :0.50 
 100:1499850 
 
 
 Mean : 669934 
 Mean : 50 
 Mean :0.50 
 NA 
 
 
 3rd Qu.:1024898 
 3rd Qu.: 75 
 3rd Qu.:0.75 
 NA 
 
 
 Max. :1499850 
 Max. :100 
 Max. :0.99 
 NA 
 
 
 
 
 
  
  
  
 
 
 
 ticks 
 langval_all 
 network 
 
 
 
 
 Min. :500 
 Min. :0.09326 
 random :1499850 
 
 
 1st Qu.:500 
 1st Qu.:0.41813 
 scalefree :1499850 
 
 
 Median :500 
 Median :0.58055 
 smallworld:1499850 
 
 
 Mean :500 
 Mean :0.53937 
 NA 
 
 
 3rd Qu.:500 
 3rd Qu.:0.68430 
 NA 
 
 
 Max. :500 
 Max. :0.94574 
 NA 
 
 
 
 
 
 synchr_versus_asynchr.csv 
 Here is a summary of our dataset  synchr_versus_asynchr.csv : 
 The aim of this dataset is just to make a comparison of the results in an synchronous versus asynchronous network. 
 
 Table continues below 
 
  
  
  
  
 
 
 
 rep_id 
 Condition 
 prop_biased 
 strength_bias 
 
 
 
 
 Min. : 1 
 Asynchronous:8008000 
 Min. : 0 
 Min. :0.10 
 
 
 1st Qu.:2001 
 Synchronous :8008000 
 1st Qu.: 10 
 1st Qu.:0.10 
 
 
 Median :4000 
 NA 
 Median : 30 
 Median :0.35 
 
 
 Mean :4000 
 NA 
 Mean : 38 
 Mean :0.35 
 
 
 3rd Qu.:6000 
 NA 
 3rd Qu.: 50 
 3rd Qu.:0.60 
 
 
 Max. :8000 
 NA 
 Max. :100 
 Max. :0.60 
 
 
 
 
 Table continues below 
 
  
  
  
  
 
 
 
 size_net 
 ticks 
 langval_all 
 network 
 
 
 
 
 10 agents :4004000 
 Min. : 0 
 Min. :0.09245 
 Random :8008000 
 
 
 150 agents:4004000 
 1st Qu.: 250 
 1st Qu.:0.41330 
 Scale-free:8008000 
 
 
 50 agents :4004000 
 Median : 500 
 Median :0.59936 
 NA 
 
 
 500 agents:4004000 
 Mean : 500 
 Mean :0.54022 
 NA 
 
 
 NA 
 3rd Qu.: 750 
 3rd Qu.:0.70144 
 NA 
 
 
 NA 
 Max. :1000 
 Max. :0.93073 
 NA 
 
 
 
 
 
  
  
  
 
 
 
 influencers_biased 
 learners 
 init_langval 
 
 
 
 
 Min. :0 
 SAM:16016000 
 Min. :4 
 
 
 1st Qu.:0 
 NA 
 1st Qu.:4 
 
 
 Median :0 
 NA 
 Median :4 
 
 
 Mean :0 
 NA 
 Mean :4 
 
 
 3rd Qu.:0 
 NA 
 3rd Qu.:4 
 
 
 Max. :0 
 NA 
 Max. :4 
 
 
 
 
 
 stabilization.csv 
 Here is a summary of our dataset  stabilization.csv : 
 This dataset aims to compare the precision of two methods (rounding by 10,000 and rounding by 100,000) to compute the stabilization time, for only one condition. 
 
 Table continues below 
 
  
  
  
  
  
 
 
 
 rep_id 
 prop_biased 
 learners 
 bias_strength 
 size_net 
 
 
 
 
 Min. :4701 
 10:100 
 SAM:100 
 0.1:100 
 500:100 
 
 
 1st Qu.:4726 
 NA 
 NA 
 NA 
 NA 
 
 
 Median :4750 
 NA 
 NA 
 NA 
 NA 
 
 
 Mean :4750 
 NA 
 NA 
 NA 
 NA 
 
 
 3rd Qu.:4775 
 NA 
 NA 
 NA 
 NA 
 
 
 Max. :4800 
 NA 
 NA 
 NA 
 NA 
 
 
 
 
 Table continues below 
 
  
  
  
  
 
 
 
 init_langval 
 influencers_biased 
 network 
 stab_control 
 
 
 
 
 4:100 
 10:100 
 Scale-free:100 
 Min. : 51.00 
 
 
 NA 
 NA 
 NA 
 1st Qu.: 88.75 
 
 
 NA 
 NA 
 NA 
 Median :133.00 
 
 
 NA 
 NA 
 NA 
 Mean :128.64 
 
 
 NA 
 NA 
 NA 
 3rd Qu.:157.00 
 
 
 NA 
 NA 
 NA 
 Max. :238.00 
 
 
 
 
 
  
  
  
 
 
 
 stab_biased 
 stab_biased_precise 
 stab_control_precise 
 
 
 
 
 Min. : 66.0 
 Min. :1592 
 Min. : 384.0 
 
 
 1st Qu.:241.8 
 1st Qu.:2323 
 1st Qu.: 590.5 
 
 
 Median :327.5 
 Median :2848 
 Median : 690.0 
 
 
 Mean :339.1 
 Mean :2848 
 Mean : 725.0 
 
 
 3rd Qu.:426.2 
 3rd Qu.:3320 
 3rd Qu.: 852.5 
 
 
 Max. :697.0 
 Max. :4415 
 Max. :1315.0 
 
 
 
 
 
 
 
 Example of language change through time for specific conditions 
 Here, we visualize what happens through time for biased and unbiased agents, in 4 different conditions, using  example_time.csv : 
 
 strong bias and 0% of influencers; 
 weak bias and 0% of influencers; 
 strong bias and 10% of influencers; 
 weak bias and 10% of influencers. 
 
 
 
 
 Figure 17.  Language (vertical axis, as language values) is changing across time (horizontal axis, in ticks) in a scale-free network with 500 SAM agents of which 10% are biased. Each individual curve represents the mean language value of the biased minority (blue) and the unbiased majority (light green) for 100 independent replications. The black curve shows the aggregated mean of the different replications. Top: the minority is strongly biased; bottom: the minority is weakly biased. Left: the biased minority is not overrepresented among the most influential agents in the network; right: the 10% most influential agents are occupied by biased agents.
 
 
 
 
 
 
 
 
 
 
 
 
 
 
 
 
 
 
 
 
 
 
 
 
 
 
 
 
 
 
 
 
 
 
 
 
 
 
 
 
 
 
 
 
 
 
 
 
 
 
 
 
 
 
 
 
 
 
 
 
 
 
 
 
 
 
 
 
 
 
 Analysis 
 In this study, we focus on all our dependent variables, using the file  analysis.csv : 
 
 Language value (after 5000 ticks) for biased, unbiased and all agents 
 Difference between unbiased and biased agents (after 5000 ticks) 
 Stabilization time for biased, unbiased and all agents 
 Dissemination for biased, unbiased and all agents 
 Heterogeneity intra and inter group (after 5000 ticks) 
 
 
 Final value of language 
 
 Regression 
 We apply a classic linear regression model to our data using the function  lm . We study : 
 
 the final value of the language for  all  agents at tick = 5000 ; 
 the final value of the language for  unbiased  agents at tick = 5000 ; 
 the final value of the language for  biased  agents at tick = 5000. 
 
 
 1) For the final value of all agents : 
  
Call:
lm(formula = langval_all_5000 ~ prop_biased + bias_strength + 
    size_net + learners + network + influencers_biased + init_langval, 
    data = subdata_lm)

Residuals:
     Min       1Q   Median       3Q      Max 
-0.58174 -0.08470 -0.00891  0.09004  0.62858 

Coefficients:
                     Estimate Std. Error  t value Pr(>|t|)    
(Intercept)         0.3716610  0.0006272  592.559  < 2e-16 ***
prop_biased        -0.1499061  0.0003136 -478.005  < 2e-16 ***
bias_strength       0.0997760  0.0003136  318.155  < 2e-16 ***
size_net            0.0028496  0.0003136    9.086  < 2e-16 ***
learnersSAM        -0.0032419  0.0006272   -5.169 2.36e-07 ***
networkScale-free  -0.0104092  0.0007682  -13.551  < 2e-16 ***
networkSmall-world  0.0087383  0.0007682   11.375  < 2e-16 ***
influencers_biased -0.0041089  0.0003136  -13.102  < 2e-16 ***
init_langval        0.0919605  0.0003136  293.234  < 2e-16 ***
---
Signif. codes:  0 '***' 0.001 '**' 0.01 '*' 0.05 '.' 0.1 ' ' 1

Residual standard error: 0.1331 on 179991 degrees of freedom
Multiple R-squared:  0.6983,    Adjusted R-squared:  0.6983 
F-statistic: 5.208e+04 on 8 and 179991 DF,  p-value: < 2.2e-16  
 Plot: 
 
 
 
 Figure 18.  Effect of different variables on the final value of language (for all agents).
 
 
 
 
 2) For the final value of unbiased agents : 
  
Call:
lm(formula = langval_control_5000 ~ prop_biased + bias_strength + 
    size_net + learners + network + influencers_biased + init_langval, 
    data = subdata_lm)

Residuals:
     Min       1Q   Median       3Q      Max 
-0.64490 -0.07566  0.00850  0.07953  0.57469 

Coefficients:
                     Estimate Std. Error  t value Pr(>|t|)    
(Intercept)         0.3162329  0.0006841  462.268   <2e-16 ***
prop_biased        -0.2544512  0.0005854 -434.670   <2e-16 ***
bias_strength       0.0940047  0.0003172  296.369   <2e-16 ***
size_net            0.0043042  0.0003172   13.570   <2e-16 ***
learnersSAM        -0.0096914  0.0006344  -15.277   <2e-16 ***
networkScale-free   0.0068113  0.0007769    8.767   <2e-16 ***
networkSmall-world  0.0133597  0.0007769   17.195   <2e-16 ***
influencers_biased -0.0049467  0.0003172  -15.596   <2e-16 ***
init_langval        0.1023487  0.0003172  322.675   <2e-16 ***
---
Signif. codes:  0 '***' 0.001 '**' 0.01 '*' 0.05 '.' 0.1 ' ' 1

Residual standard error: 0.1204 on 143991 degrees of freedom
  (36000 observations deleted due to missingness)
Multiple R-squared:  0.7262,    Adjusted R-squared:  0.7262 
F-statistic: 4.773e+04 on 8 and 143991 DF,  p-value: < 2.2e-16  
 Plot: 
 
 
 
 Figure 19.  Effect of different variables on the final value of language (for unbiased agents).
 
 
 
 
 3) For the final value of biased agents : 
  
Call:
lm(formula = langval_biased_5000 ~ prop_biased + bias_strength + 
    size_net + learners + network + influencers_biased + init_langval, 
    data = subdata_lm)

Residuals:
     Min       1Q   Median       3Q      Max 
-0.47198 -0.07336  0.00784  0.08097  0.57066 

Coefficients:
                     Estimate Std. Error  t value Pr(>|t|)    
(Intercept)         0.2973903  0.0006246  476.153  < 2e-16 ***
prop_biased        -0.0719664  0.0003276 -219.678  < 2e-16 ***
bias_strength       0.1479755  0.0003092  478.601  < 2e-16 ***
size_net           -0.0008235  0.0003092   -2.663  0.00774 ** 
learnersSAM         0.0074573  0.0006184   12.060  < 2e-16 ***
networkScale-free  -0.0507229  0.0007573  -66.975  < 2e-16 ***
networkSmall-world -0.0033064  0.0007573   -4.366 1.27e-05 ***
influencers_biased -0.0060186  0.0003092  -19.466  < 2e-16 ***
init_langval        0.0716033  0.0003092  231.589  < 2e-16 ***
---
Signif. codes:  0 '***' 0.001 '**' 0.01 '*' 0.05 '.' 0.1 ' ' 1

Residual standard error: 0.1173 on 143991 degrees of freedom
  (36000 observations deleted due to missingness)
Multiple R-squared:  0.7007,    Adjusted R-squared:  0.7007 
F-statistic: 4.214e+04 on 8 and 143991 DF,  p-value: < 2.2e-16  
 Plot: 
 
 
 
 Figure 20.  Effect of different variables on the final value of language (for biased agents).
 
 
 
 
 Conclusion: 
 All variables have a statistically significant effect on the language value. However, only  prop_biased ,  bias_strength  and  init_langval  have a big effect size. The variable  network , when its value is  Scale-free , might have a small effect on language value too. Finally, it seems that the effect of the variables  size_net ,  learners  and  influencers_biased  is negligible. 
 
 
 
 Plot specific variables 
 According to the results of the regression analysis, we have hints of what variables are interesting for our analysis. Then, we study specifically some combination of variables, without aggregating, in order to grasp the interesting content of the analysis. 
  Note : when it not specifically mentionned, the parameters we use are  network  =  Scale-free ,  learners  =  SAM ,  size_net  =  150 ,  influencers_biased  =  0 ,  init_langval  =  4 . 
 
 With an initial value of the language in the society 
 
 
 
 Figure 21.  Effect of size and network type in a network with an initial language in the society (SAM, no influencers). The top brown line indicate the initial value of the language for unbiased agents, while the dotted lines indicate the initial value of the language for biased agents (lower line: strongly biased, middle line: weakly biased).
 
 
 
 
 Without an initial value of the language in the society 
 
 
 
 Figure 22.  Effect of size and network type in a network without an initial language in the society (SAM, no influencers). The top brown line indicate the initial value of the language for unbiased agents, while the dotted lines indicate the initial value of the language for biased agents (lower line: strongly biased, middle line: weakly biased)
 
 
 
 
 Main variables only 
 Here, we plot only the 3 variables that had an impact on the final value of the language, namely: 
 
  prop_biased  
  bias_strength  
  init_langval  
 
 
 
 
 Figure 24.  The final language value of the whole population for a scale-free network with 150 SAM agents. The solid line (1) shows the initial value of the language for the unbiased agents, while the dotted lines (2a and 2b) show the initial value of the language for biased agents (a: weakly biased and b: strongly biased). The horizontal axis shows the different cases considered (combinations of bias strength and proportion of biased agents in the populations), the vertical axis is the language value of the population, and the colored boxplots show the distribution of the language values among the biased (purple) and unbiased (green) agents.
 
 
 
 
 
 
 
 
 
 
 
 
 
 
 
 
 
 
 
 
 
 
 % -->
 
 
 
 
 
 
 
  subdata -->
 
 
 
 
 
 
 
 
 
 
 
 
 
 
 
 
 
 
 
 Influencers effect 
 Is there an effect of influencers? In the following plot, we focus  scale-free  networks. 
 
 
 
 Figure 25.  Effect of influencers in Scale-free networks (SAM, 150 agents, initial language).
 
 
 The percentage of influencers biased does not have a strong impact on our results. It has a small effect on the language value of the population if the network is very small, and if there are 10% of strongly biased agents in the network. We also observe a difference between biased and unbiased agents in big network when there are influencers biased; see  Difference between unbiased and biased agents  for more information. 
 
 
 Conclusion: 
 
 Logically, the more biased agents  and  the strongly biased they are, the lower the language value will be after 5000 ticks. 
 The initial society language also has a strong positive impact on the final value of the language of the society. 
 When only 10% of the population is strongly biased and when the network is very small, the presence of influencers will drag down the language value of the society in scale-free networkq. 
 
 
 
 
 Statistics 
 
 Hypothesis 
 Our hypothesis is that the presence of biased agents in the population have an impact on the language of the society. More specifically, it means that the language value of a population in which we introduced biased agents is significantly different from the language value of a population in which we have only unbiased agents. 
  Note : of course, this depends of the amount of biased agents introduced in the network. We used the value 10, 30 and 50% of biased agents to do this analysis. For a finer analysis, please refer to  Systematic bias effect study . 
 
 
 Wilcoxon test 
 In order to test this hypothesis, we performed unpaired Wilcoxon (unpaired) tests for all possible combinations of parameters, comparing: 
 
 the language value of unbiased agents at tick 5000 in a population  without  biased agents 
 the language value of unbiased agents at tick 5000 in a population  with  biased agents (10%, 30% or 50%) 
 
 The goal is to check whether in some condition, these languages values are not statistically significant. We corrected the p-values for multiple testing using the Bonferroni method, and we print here all condition where the difference is not significant (p>0.05). The conditions are written the following way: 
 “ size_net  -  init_langval  -  influencers_biased  -  learners  -  network  -  prop_biased  -  bias_strength ” 
   [1] "  10-0-10-MAP-Random-10-0.6"      "  10-4-10-MAP-Random-10-0.6"     
 [3] " 150-0-10-MAP-Random-10-0.6"      " 500-0-10-MAP-Random-10-0.6"     
 [5] "  10-0- 0-MAP-Random-10-0.6"      "  10-4- 0-MAP-Random-10-0.6"     
 [7] "  50-0- 0-MAP-Random-10-0.6"      "  50-4- 0-MAP-Random-10-0.6"     
 [9] " 150-0- 0-MAP-Random-10-0.6"      " 500-0- 0-MAP-Random-10-0.6"     
[11] "1000-0- 0-MAP-Random-10-0.6"      "  10-4-10-SAM-Random-10-0.6"     
[13] " 500-0-10-SAM-Random-10-0.6"      " 500-4-10-SAM-Random-10-0.6"     
[15] "  10-0- 0-SAM-Random-10-0.6"      "  50-0- 0-SAM-Random-10-0.6"     
[17] "  50-4- 0-SAM-Random-10-0.6"      " 150-0- 0-SAM-Random-10-0.6"     
[19] " 150-4- 0-SAM-Random-10-0.6"      " 500-0- 0-SAM-Random-10-0.6"     
[21] "1000-4- 0-SAM-Random-10-0.6"      "  10-0-10-MAP-Small-world-10-0.6"
[23] "  10-4-10-MAP-Small-world-10-0.6" "  50-0-10-MAP-Small-world-10-0.6"
[25] "  10-4-10-MAP-Small-world-30-0.6" "  10-0- 0-MAP-Small-world-10-0.6"
[27] "  10-4- 0-MAP-Small-world-10-0.6" "  50-0- 0-MAP-Small-world-10-0.6"
[29] "  10-0- 0-MAP-Small-world-30-0.6" "  10-0-10-SAM-Small-world-10-0.6"
[31] "  10-4-10-SAM-Small-world-10-0.6" "  50-0-10-SAM-Small-world-10-0.6"
[33] " 150-0-10-SAM-Small-world-10-0.6" "  10-0-10-SAM-Small-world-30-0.6"
[35] "  10-0- 0-SAM-Small-world-10-0.1" "  10-0- 0-SAM-Small-world-10-0.6"
[37] "  10-4- 0-SAM-Small-world-10-0.6" "  50-4- 0-SAM-Small-world-10-0.6"
[39] "  10-4- 0-SAM-Small-world-30-0.6" "  10-0-10-MAP-Scale-free-10-0.6" 
[41] "  10-0- 0-MAP-Scale-free-10-0.6"  "  10-4- 0-MAP-Scale-free-10-0.6" 
[43] "  50-0- 0-MAP-Scale-free-10-0.6"  "  10-0-10-SAM-Scale-free-10-0.6" 
[45] "  10-0- 0-SAM-Scale-free-10-0.6"  "  10-4- 0-SAM-Scale-free-10-0.6" 
[47] "  50-0- 0-SAM-Scale-free-10-0.6"   
 
 
 Conclusion on statistics 
 Except for 47 cases out of 1080 cases, the language value of network with biased agents is always significantly different from the language value of network without biased agents. 
 These adjusted p-values show that, in the vast majority of the combinations, the language values of the unbiased agents in a society with biased agents are significantly different from those of an homogeneous unbiased population. Among the replications with no significant differences, most were networks with only 10 agents, and the remaining were random or small-world networks with a low percentage (10%) of weakly biased agents. 
 
 
 
 Main conclusion 
 
 Indeed, the presence of biased agents impacts the language value of the society. See  Systematic bias effect study  to see in what extent it is true. 
 The final value of the population language is shaped by proportion of biased agents, the strength of their bias and the initial language of the society. 
 
 
 
 
 Difference between biased and unbiased agents 
 
 Regression 
 We apply a classic linear regression model to our data using the function  lm  on the variable  diff_group.  
  
Call:
lm(formula = diff_group ~ prop_biased + bias_strength + size_net + 
    learners + network + influencers_biased + init_langval, data = subdata_lm)

Residuals:
     Min       1Q   Median       3Q      Max 
-0.19089 -0.04471 -0.01400  0.02952  0.57661 

Coefficients:
                     Estimate Std. Error  t value Pr(>|t|)    
(Intercept)         0.0160487  0.0004923   32.596  < 2e-16 ***
prop_biased        -0.0916558  0.0005189 -176.629  < 2e-16 ***
bias_strength      -0.0368739  0.0002391 -154.217  < 2e-16 ***
size_net            0.0070447  0.0002391   29.463  < 2e-16 ***
learnersSAM        -0.0039402  0.0004782   -8.240  < 2e-16 ***
networkScale-free   0.0649399  0.0005857  110.879  < 2e-16 ***
networkSmall-world  0.0216578  0.0005857   36.979  < 2e-16 ***
influencers_biased  0.0013970  0.0002391    5.843 5.16e-09 ***
init_langval        0.0148948  0.0002391   62.294  < 2e-16 ***
---
Signif. codes:  0 '***' 0.001 '**' 0.01 '*' 0.05 '.' 0.1 ' ' 1

Residual standard error: 0.07858 on 107991 degrees of freedom
  (72000 observations deleted due to missingness)
Multiple R-squared:  0.4019,    Adjusted R-squared:  0.4019 
F-statistic:  9073 on 8 and 107991 DF,  p-value: < 2.2e-16  
 Plot: 
 
 
 
 Figure 26.  Effect of different variables on the difference between unbiased and biased agents.
 
 
 
 Conclusion on regression 
 All variables have a statistically significant effect on the language value. However, only  network  and  prop_biased  have a quite big effect size. The variable  bias_strength  might have a small effect on language value too. Finally, it seems that the effect of the variables  size_net ,  init_langval ,  learners  and  influencers_biased  is negligible. 
 
 
 
 Plot specific variables 
 
 Differences between network types 
 
 
 
 Figure 27.  The difference between the languages of the unbiased and the biased agents after 1,000 iterations, function of network type (panels) and size (color), and bias frequency and strength (horizontal axis). We used SAM agents, there is no enrichment of biased agents among the top influencers, and agents were exposed to an initial language.
 
 
 
 
 Influencers effect 
 
 
 
 Figure 28.  Effect of influencers on the difference between unbiased and biased agents in scale-free networks (SAM, initial language).
 
 
 
 
 Conclusion 
 After exploring a lot of different combinations, we found: 
 
   differences between network types  : 
 
  network size . On the one hand, the differences between biased and unbiased agents become smaller and smaller with size in random networks (no differences in big networks!). On the other hand, in scale-free and small-world networks, the difference between the biased and unbiased increases with the network’s size. 
  influencers effect . The presence of biased influencers has an effect only in scale-free network, especially if  prop_biased  = 10% and the society is big. 
  
   interaction of prop_biased and bias_strength . In all networks, a low proportion of strongly biased agents amplify the differences between biased and unbiased agents.  
 
 
 
 
 Statistics 
 
 Hypothesis 
 Our hypothesis is that the biased agents keep a trace of their bias inside their everyday language, even after interacting with unbiased agents. More specifically, it means that after 5000 ticks, there would still be a difference in the language value of the biased and unbiased agents. 
 
 
 Wilcoxon test 
 For all sets of conditions (100 replications), we computed a Wilcoxon test between: 
 
 the language value of  biased  agents at tick = 5000; 
 the language value  unbiased  agents at tick = 5000. 
 
 Then, we adjusted the p-value using Bonferroni method. 
 
 
 Conclusion: 
 We found that this is  not significant  for 429 out of 1080 of the conditions, when considering a corrected p-value = 0.05. 
 More specifically, we found that it is  not significant  for: 
 
 123 cases out of 216 for networks with only 10 nodes 
 114 cases out of 216 for networks with only 50 nodes 
 77 cases out of 216 for networks with only 150 nodes 
 60 cases out of 216 for networks with only 500 nodes 
 55 cases out of 216 for networks with only 1000 nodes 
 
 Looking more precisely at the network type, we find that it is  not significant  for: 
 
 226 cases out of 360 for random networks 
 66 cases out of 360 for scale-free networks 
 137 cases out of 360 for small-world networks 
 
 To conclude, the adjusted p-values are almost always significant for scale-free networks (except for small networks with 10 or 50 agents, often weakly biased); significant for half of the small-world networks, especially for big networks (with more than 150 agents) with strong biases; however, most random networks do not show a significant difference, with the exception of a few very small networks (10 or 50 agents). 
 
 
 
 Main conclusion 
 
  In  scale-free  networks (and small-world to a smaller extent), biased agents keep something of their bias in their everyday language, even after interacting with other agents.  
  The stronger the bias, the bigger the difference between the biased and unbiased agents at the end, which is expectable. But interestingly, the more biased agents, the less difference!  10% of strongly biased  agents seems to be the condition where the difference is the highest.  
  Influencers have a strong effect on the difference between biased and unbiased agents, especially on scale-free networks: they increase this difference, especially in big networks with 10% of biased agents.  
 
 
 
 
 Stabilisation time 
 
 Regression 
 
 With stabilization for the whole population 
  
Call:
lm(formula = stab_all ~ prop_biased + bias_strength + size_net + 
    learners + network + influencers_biased + init_langval, data = subdata_lm)

Residuals:
    Min      1Q  Median      3Q     Max 
-256.60  -84.36  -27.10   51.14  891.41 

Coefficients:
                   Estimate Std. Error  t value Pr(>|t|)    
(Intercept)        114.0759     0.5796  196.822  < 2e-16 ***
prop_biased        -36.1143     0.2898 -124.620  < 2e-16 ***
bias_strength      -22.7479     0.2898  -78.496  < 2e-16 ***
size_net           -50.0487     0.2898 -172.703  < 2e-16 ***
learnersSAM          5.2985     0.5796    9.142  < 2e-16 ***
networkScale-free   48.8077     0.7099   68.758  < 2e-16 ***
networkSmall-world  -8.0992     0.7099  -11.410  < 2e-16 ***
influencers_biased  -1.6083     0.2898   -5.550 2.87e-08 ***
init_langval        17.5295     0.2898   60.489  < 2e-16 ***
---
Signif. codes:  0 '***' 0.001 '**' 0.01 '*' 0.05 '.' 0.1 ' ' 1

Residual standard error: 122.9 on 179991 degrees of freedom
Multiple R-squared:  0.2587,    Adjusted R-squared:  0.2587 
F-statistic:  7852 on 8 and 179991 DF,  p-value: < 2.2e-16  
 Plot: 
 
 
 
 Figure 30.  Effect of different variables on the the stabilization time (for final agents).
 
 
 
 
 With stabilization only for biased agents 
  
Call:
lm(formula = stab_biased ~ prop_biased + bias_strength + size_net + 
    learners + network + influencers_biased + init_langval, data = subdata_lm)

Residuals:
    Min      1Q  Median      3Q     Max 
-261.63  -90.64  -24.94   52.38 1249.09 

Coefficients:
                   Estimate Std. Error  t value Pr(>|t|)    
(Intercept)         97.7797     0.7310  133.768  < 2e-16 ***
prop_biased        -50.6211     0.3834 -132.030  < 2e-16 ***
bias_strength       33.8842     0.3619   93.641  < 2e-16 ***
size_net           -31.6523     0.3619  -87.473  < 2e-16 ***
learnersSAM          3.2520     0.7237    4.494 7.01e-06 ***
networkScale-free   80.3580     0.8863   90.662  < 2e-16 ***
networkSmall-world  10.1480     0.8863   11.449  < 2e-16 ***
influencers_biased  -5.3389     0.3619  -14.754  < 2e-16 ***
init_langval        14.3716     0.3619   39.717  < 2e-16 ***
---
Signif. codes:  0 '***' 0.001 '**' 0.01 '*' 0.05 '.' 0.1 ' ' 1

Residual standard error: 137.3 on 143991 degrees of freedom
  (36000 observations deleted due to missingness)
Multiple R-squared:  0.2398,    Adjusted R-squared:  0.2397 
F-statistic:  5677 on 8 and 143991 DF,  p-value: < 2.2e-16  
 Plot: 
 
 
 
 Figure 31.  Effect of different variables on the the stabilization time (for biased agents).
 
 
 
 
 With stabilization only for unbiased agents 
  
Call:
lm(formula = stab_control ~ prop_biased + bias_strength + size_net + 
    learners + network + influencers_biased + init_langval, data = subdata_lm)

Residuals:
    Min      1Q  Median      3Q     Max 
-318.11 -100.83  -28.24   67.13 1158.73 

Coefficients:
                   Estimate Std. Error  t value Pr(>|t|)    
(Intercept)        184.3421     0.8300  222.090  < 2e-16 ***
prop_biased         52.4866     0.7103   73.896  < 2e-16 ***
bias_strength      -60.1999     0.3849 -156.421  < 2e-16 ***
size_net           -53.2379     0.3849 -138.331  < 2e-16 ***
learnersSAM          3.7148     0.7697    4.826 1.39e-06 ***
networkScale-free   86.6650     0.9427   91.933  < 2e-16 ***
networkSmall-world  -3.0080     0.9427   -3.191  0.00142 ** 
influencers_biased  -2.8401     0.3849   -7.380 1.60e-13 ***
init_langval        30.4739     0.3849   79.182  < 2e-16 ***
---
Signif. codes:  0 '***' 0.001 '**' 0.01 '*' 0.05 '.' 0.1 ' ' 1

Residual standard error: 146 on 143991 degrees of freedom
  (36000 observations deleted due to missingness)
Multiple R-squared:  0.3178,    Adjusted R-squared:  0.3178 
F-statistic:  8386 on 8 and 143991 DF,  p-value: < 2.2e-16  
 Plot: 
 
 
 
 Figure 32.  Effect of different variables on the the stabilization time (for unbiased agents).
 
 
 
 
 Conclusion: 
 All variables have a statistically significant effect on the language value for the stabilization value of biased and unbiased agents. 
 However, only  network  have a quite big effect size, especially for scale-free networks. The variables  size_net ,  prop_biased  and  bias_strength  (to a lesser extent) might have a small effect on language value too. The effect of  learners ,  init_langval  and  influencers_biased  is negligible. 
 
 
 
 Plot specific variables 
 
 Size and network 
 
 
 
 Figure 33.  Stabilization time for the biased and the unbiased agents (color), in different types of networks (columns) with two different sizes (rows), for various bias frequencies and strength (horizontal axis). The agents are SAM, there are no biased influencers, and there is an initial language.
 
 
 
 
 Focus on biased agents 
 Here, we study the stabilization time for biased agents only: 
 
 
 
 Figure 34.  Effect of network type and size on stabilization time for biased agents only (SAM, no influencers, initial language).
 
 
 
 
 Focus on unbiased agents 
 Here, we study the stabilization time for unbiased agents only: 
 
 
 
 Figure 35.  Effect of network type and size on stabilization time for biased agents only (SAM, no influencers, initial language).
 
 
 
 
 Conclusion: 
 
  Interaction between  size_net  and  network : in random networks, agents stabilize faster when the network is big, while in scale-free and small-world networks, stabilization takes the same amount of time in big versus small networks.  
  In general, the language value (for biased and unbiased agents) stabilizes faster in networks with  weakly biased  agents.  
  In general,  scale-free  networks are the longer to stabilize.  
  Biased agents need more time to stabilize when only a small percentage of agents is strongly biased (10%).  
 
 
 
 
 
 Dissemination 
 We study here the dissemination of results across the 100 replications. The higher the dissemination, the more different the results of language value at final tick across replications. 
 
 Regression 
 
 For whole population 
  
Call:
lm(formula = std_cond_all ~ prop_biased + bias_strength + size_net + 
    learners + network + influencers_biased + init_langval, data = data_dissemination_lm)

Residuals:
     Min       1Q   Median       3Q      Max 
-0.05243 -0.01790 -0.00449  0.01178  0.17186 

Coefficients:
                     Estimate Std. Error t value Pr(>|t|)    
(Intercept)         7.559e-02  2.023e-03  37.365  < 2e-16 ***
prop_biased        -4.529e-04  1.926e-05 -23.518  < 2e-16 ***
bias_strength       4.805e-02  2.600e-03  18.479  < 2e-16 ***
size_net           -4.316e-05  1.837e-06 -23.497  < 2e-16 ***
learnersSAM        -5.615e-03  1.365e-03  -4.114 4.07e-05 ***
networkScale-free  -9.072e-03  1.672e-03  -5.427 6.53e-08 ***
networkSmall-world -3.134e-03  1.672e-03  -1.875    0.061 .  
influencers_biased -1.262e-04  1.365e-04  -0.925    0.355    
init_langval       -2.798e-03  3.412e-04  -8.200 4.52e-16 ***
---
Signif. codes:  0 '***' 0.001 '**' 0.01 '*' 0.05 '.' 0.1 ' ' 1

Residual standard error: 0.02895 on 1791 degrees of freedom
Multiple R-squared:  0.4659,    Adjusted R-squared:  0.4635 
F-statistic: 195.3 on 8 and 1791 DF,  p-value: < 2.2e-16  
 Plot: 
 
 
 
 Figure 36.  Effect of different variables on dissemination (for all agents).
 
 
 
 
 For biased agents 
  
Call:
lm(formula = std_cond_biased ~ prop_biased + bias_strength + 
    size_net + learners + network + influencers_biased + init_langval, 
    data = data_dissemination_lm)

Residuals:
      Min        1Q    Median        3Q       Max 
-0.045393 -0.017175 -0.002626  0.010776  0.154220 

Coefficients:
                     Estimate Std. Error t value Pr(>|t|)    
(Intercept)         3.964e-02  2.032e-03  19.507  < 2e-16 ***
prop_biased        -2.404e-04  1.955e-05 -12.297  < 2e-16 ***
bias_strength       8.376e-02  2.492e-03  33.615  < 2e-16 ***
size_net           -2.914e-05  1.760e-06 -16.558  < 2e-16 ***
learnersSAM        -3.242e-03  1.308e-03  -2.478   0.0133 *  
networkScale-free  -8.671e-03  1.602e-03  -5.413 7.25e-08 ***
networkSmall-world -1.467e-03  1.602e-03  -0.916   0.3598    
influencers_biased -6.587e-05  1.308e-04  -0.504   0.6146    
init_langval       -1.366e-03  3.270e-04  -4.176 3.14e-05 ***
---
Signif. codes:  0 '***' 0.001 '**' 0.01 '*' 0.05 '.' 0.1 ' ' 1

Residual standard error: 0.02482 on 1431 degrees of freedom
Multiple R-squared:  0.5299,    Adjusted R-squared:  0.5272 
F-statistic: 201.6 on 8 and 1431 DF,  p-value: < 2.2e-16  
 Plot: 
 
 
 
 Figure 37.  Effect of different variables on on dissemination (for biased agents).
 
 
 
 
 For unbiased agents 
  
Call:
lm(formula = std_cond_unbiased ~ prop_biased + bias_strength + 
    size_net + learners + network + influencers_biased + init_langval, 
    data = data_dissemination_lm)

Residuals:
      Min        1Q    Median        3Q       Max 
-0.055972 -0.019887 -0.004978  0.013338  0.162304 

Coefficients:
                     Estimate Std. Error t value Pr(>|t|)    
(Intercept)         8.939e-02  2.402e-03  37.214  < 2e-16 ***
prop_biased        -6.287e-04  4.167e-05 -15.086  < 2e-16 ***
bias_strength       3.540e-02  3.049e-03  11.611  < 2e-16 ***
size_net           -5.235e-05  2.154e-06 -24.305  < 2e-16 ***
learnersSAM        -7.458e-03  1.600e-03  -4.660 3.45e-06 ***
networkScale-free  -7.888e-03  1.960e-03  -4.024 6.02e-05 ***
networkSmall-world -4.460e-03  1.960e-03  -2.275    0.023 *  
influencers_biased -1.522e-04  1.600e-04  -0.951    0.342    
init_langval       -3.511e-03  4.001e-04  -8.775  < 2e-16 ***
---
Signif. codes:  0 '***' 0.001 '**' 0.01 '*' 0.05 '.' 0.1 ' ' 1

Residual standard error: 0.03037 on 1431 degrees of freedom
Multiple R-squared:  0.4276,    Adjusted R-squared:  0.4244 
F-statistic: 133.6 on 8 and 1431 DF,  p-value: < 2.2e-16  
 Plot: 
 
 
 
 Figure 38.  Effect of different variables on dissemination (for unbiased agents).
 
 
 
 
 Conclusion: 
 All variables have a statistically significant effect on the language value for the stabilization value of biased and unbiased agents. However, only  bias_strength  and  network  have a quite big effect size, especially for scale-free networks. The variables  learners  and  init_langval  might have a small effect on language value too. The effect of  size_net ,  prop_biased  and  influencers_biased  is negligible. 
 
 
 
 Plot specific variables 
 The dissemination of biased and unbiased agents is approximately the same as the dissemination results for all agents: thus, we will plot only the dissemination for all agents in the following plot. 
 
 
 
 Figure 39.  Effect of network type and size on dissemination (SAM, no influencers, initial language).
 
 
 
 Conclusion: 
 
  the dissemination of results is always higher when the agents were not initialized with an initial language of the society ;  
  dissemination of replications in  random  networks is higher compared to  scale-free  and  small-world  networks;  
  in  random  network, the dissemination of results increases when the bias is weak.  
 
 
 
 
 
 Linguistic communities and heterogeneity 
 Here, we use two variables : 
 
  het_inter_5000  : heterogeneity  between  linguistic communities 
  het_intra_5000  : : heterogeneity  within  linguistic communities 
 
 Please note that when we refer to communities here, we always refer to the  linguistic  communities. The  structural  communities detected by Louvain algorithm are fixed at the beginning of the network (see  Community detection ). 
 
 Heterogeneity between communities 
 
 Regression 
  
Call:
lm(formula = het_inter_5000 * 100 ~ prop_biased + bias_strength + 
    size_net + learners + network + influencers_biased + init_langval, 
    data = subdata_lm)

Residuals:
   Min     1Q Median     3Q    Max 
-9.017 -2.238 -0.522  1.406 36.611 

Coefficients:
                    Estimate Std. Error  t value Pr(>|t|)    
(Intercept)         1.420757   0.017265   82.291  < 2e-16 ***
prop_biased        -1.468146   0.008637 -169.983  < 2e-16 ***
bias_strength      -0.208963   0.008637  -24.195  < 2e-16 ***
size_net            0.063446   0.008636    7.347 2.04e-13 ***
learnersSAM        -0.346938   0.017273  -20.085  < 2e-16 ***
networkScale-free   5.671133   0.021149  268.152  < 2e-16 ***
networkSmall-world  1.790145   0.021155   84.620  < 2e-16 ***
influencers_biased  0.134710   0.008637   15.597  < 2e-16 ***
init_langval        0.094446   0.008637   10.935  < 2e-16 ***
---
Signif. codes:  0 '***' 0.001 '**' 0.01 '*' 0.05 '.' 0.1 ' ' 1

Residual standard error: 3.662 on 179761 degrees of freedom
  (230 observations deleted due to missingness)
Multiple R-squared:  0.3697,    Adjusted R-squared:  0.3697 
F-statistic: 1.318e+04 on 8 and 179761 DF,  p-value: < 2.2e-16  
 
 
 Plot regression 
 
 
 
 Figure 40.  Effect of different variables on heterogeneity between communities.
 
 
 The  network  type has a very strong impact on heterogeneity between groups. The  proportion of biased agents  also has a small effect on it, and there also might be a small difference between SAM and MAP learners. Finally, it seems that the  size of network ,  strength of the bias , the  influencers biased  and the  initial language  have a negligible impact on heterogeneity between groups. 
 
 
 Plot specific variables 
 Is there a difference between  SAM  and  MAP  learners? 
 
 
 
 Figure 41.  Effect of network type and size on heterogeneity between communities (SAM, no influencers, initial language).
 
 
 Is there a difference when agents are  weakly   versus   strongly  biased? 
 
 
 
 Figure 42.  The difference in heterogeneity between linguistic communities function of network type (columns) and size (colors), and bias strength (rows) and frequency (horizontal axis). The networks contain SAM agents, no influencers are biased, and there is an initial language.
 
 
 
 
 Statistics 
 
 Hypothesis 
 Our hypothesis is that the presence of biased agents in the population increase the heterogeneity between communities. 
 
 
 Wilcoxon test 
 In order to test this hypothesis, we performed unpaired Wilcoxon (unpaired) tests for all possible combinations of parameters, comparing: 
 
 the heterogeneity of unbiased agents in a population  with  biased agents 
 the heterogeneity of unbiased agents in a population  without  biased agents 
 
 The goal is to check whether in some condition, these languages values are not statistically significant. We corrected the p-values for multiple testing using the Bonferroni method: 
 
  In  scale-free  networks containing  strongly  biased agents, 16 out of 96 cases are not significant.  
  In  scale-free  networks containing  weakly  biased agents, 44 out of 96 cases are not significant.  
  In  small-world  networks containing  strongly  biased agents, 20 out of 96 cases are not significant.  
  In  small-world  networks containing  weakly  biased agents, 60 out of 96 cases are not significant.  
  In  random  networks containing  strongly  biased agents, 40 out of 96 cases are not significant.  
  In  random  networks containing  weakly  biased agents, 79 out of 96 cases are not significant.  
 
 These adjusted p-values show that, in scale-free networks with a strong bias, having biased agents in the network significantly affects the emergence of linguistic communities; this is also true, to a smaller extent, for small-world networks with strongly biased agents . However, in scale-free and small-world networks containing weakly biased agents, only about half of the time the comparisons are significant; thus, the heterogeneity observed in small-world and scale-free network containing weakly biased nodes is probably mostly due to the structure of the network itself. 
 
 
 
 Conclusion 
 
  There is a clear effect of  network  type on  heterogeneity  between group: heterogeneity is higher for scale-free than for small-world networks, and higher for small-world compared to random networks.  
  The  size  of the network does not impact heterogeneity in scale-free and small-world, but impacts random networks: small random networks have an higher heterogeneity compared to big ones.  
  Finally, heterogeneity between groups is higher when the network has some diversity, and contains both biased agents  and  unbiased agents.  
 
 
 
 
 Heterogeneity within communities 
 
 Regression 
  
Call:
lm(formula = het_intra_5000 * 100 ~ prop_biased + bias_strength + 
    size_net + learners + network + influencers_biased + init_langval, 
    data = subdata_lm)

Residuals:
    Min      1Q  Median      3Q     Max 
-4.1748 -1.4066 -0.3112  0.8956 17.8445 

Coefficients:
                    Estimate Std. Error t value Pr(>|t|)    
(Intercept)         0.913336   0.010415   87.69   <2e-16 ***
prop_biased        -0.812809   0.005208 -156.08   <2e-16 ***
bias_strength      -0.407654   0.005208  -78.28   <2e-16 ***
size_net            0.704999   0.005208  135.38   <2e-16 ***
learnersSAM        -0.125530   0.010415  -12.05   <2e-16 ***
networkScale-free   2.499028   0.012756  195.91   <2e-16 ***
networkSmall-world  1.383570   0.012756  108.47   <2e-16 ***
influencers_biased -0.052981   0.005208  -10.17   <2e-16 ***
init_langval        0.172148   0.005208   33.06   <2e-16 ***
---
Signif. codes:  0 '***' 0.001 '**' 0.01 '*' 0.05 '.' 0.1 ' ' 1

Residual standard error: 2.209 on 179991 degrees of freedom
Multiple R-squared:  0.3301,    Adjusted R-squared:  0.3301 
F-statistic: 1.109e+04 on 8 and 179991 DF,  p-value: < 2.2e-16  
 
 
 Plot regression 
 
 
 
 Figure 43.  Effect of different variables on heterogeneity within communities.
 
 
 The  network  type has a very strong impact on heterogeneity within groups. The  proportion of biased agents  and the  size of the network  also has a small effect on it, and there also might be a small difference between SAM and MAP learners. Finally, it seems that the  strength of the bias , the  influencers biased  and the  initial language  have a negligible impact on heterogeneity within groups. 
 
 
 Plot specific variables 
 
 
 
 Figure 44.  Effect of network type and size on heterogeneity within communities (SAM, no influencers, initial language).
 
 
 
 
 Conclusion: 
 
  The heterogeneity within groups is higher for  scale-free  networks, compared to  small-world  networks; small-world network also have in average a higher heterogeneity within groups compared to  random  networks.  
  In  random  networks, heterogeneity within groups decreases with size, whereas it increases with size in small-world and scale-free networks.  
  Finally, heterogeneity within groups is higher when the network has some diversity: biased agents  and  unbiased agents.  
 
 
 
 
 Conclusion on heterogeneity between and within groups: 
 
  Agents in random networks have a very homogenous language value; they share almost all the same language value. In contrary, agents in small-world and scale-free networks are more heterogeneous, which corrobate the results found in  Difference between unbiased and biased agents , and can explain why the heterogeneity within groups is high in these networks.  
  Furthermore, heterogeneity between linguistic communities seems to naturally emerge in scale-free and small-world networks even with agents who are not too strongly biased; moreover, strongly biased agents amplify the language differences between linguistic communities in scale-free networks.  
 
 
 
 
 
 Systematic bias effect study 
 The following figure shows the joint influence of the proportion of biased agents and the strength of the bias on the population’s language value for the set of values in the “Systematic bias effects study” (see  Set of combination 2 - extra_analysis.csv ). We decided to further investigate the effect of these two parameters due of their large effect sizes. In this study, we ran 50 independent replications for each of all the possible combinations of: 
 
 the bias strength (going from 0.0=very strongly biased to 1.0=very weakly biased, in steps of 0.01) and 
 the proportion of biased agents in the population (going from 0% to 100% in steps of 1%). 
 
 
 
 
 Figure 45.  Limit for language value according to bias strength and % of biased people - aggregated by condition.
 
 
 For each replication, we computed the mean language value of the population after 500 iterations, and we then averaged the 50 independent replications for each combination by taking their mean: for example, the averaged mean language value of the population for the condition  {bias_strength=0.70 & prop_biased=35}  is 0.67, but is 0.22 for the condition  {bias_strength=0.15 & prop_biased=80} . In general, the aggregated mean language value progressively increases with the proportion of biased agents and the strength of the bias. 
 In order to better visualize the shape of the relationship between the bias strength and frequency (i.e., linear or not), and also to check if the proportion of biased influencers impacts the results, we also show the set of isolines for the mean language value of the population. These isolines are defined as the maximum values of the combination of bias_strength and prop_biased for a given set of language values. 
 
 
 
 Figure 46.  Limit for language value according to bias strength and % of biased people - aggregated by condition.
 
 
 Interestingly, the relationship between the strength of the bias and the proportion of biased agents is relatively linear when the proportion of biased agents is high and/or when the bias is strong, but becomes  nonlinear  for low frequencies of the biased agents and for weak biases. In this latter case, the effect of biased agents on the language value of the population is much stronger than expected. 
 Moreover, this analysis helps understanding under what conditions an initial language strongly favouring “1” may change to a language favouring the variant “0”: while only in populations with a large proportion of strongly biased agents (> 50%) does the language strongly favour “0” (a language value of 0.2), it is enough for only 15%-20% of the populations to have a strong bias for the language to reach a moderate preference for “0” (language value of 0.4). However, please note that while these particular values critically depend on the the initial language (i.e., the number of initial utterances and the distribution of “0” and “1” utterances), they do support qualitative inferences concerning the influence of biased agents in a population. 
 We also plot the same type of graphs except that we selected the  maximum  language value over the 50 replication instead of the  mean  aggregated value of the 50 replications: 
 
 
 
 Figure 47.  Limit for language value according to bias strength and % of biased people - maximum value
 
 
 
 
 Understanding SAM-MAP differences 
 During this analysis, we did not report important SAM-MAP differences. However, and thanks to a one of the reviewer’s comment, we realized this might be due to the fact that with time, the difference between SAM and MAP sampling strategies decreases. Indeed, with time, the alpha and beta parameters of the Beta distribution increases as agents hear new utterances. 
 Thus, it could be interesting to focus on the first iterations of the model. We looked at the stability in the use of variant for both strategies. That is, for each agent and for each iteration, we compute the  delta : this value indicate if the utterance at  \(t+1\)  is identical with the utterance at  \(t\) . If yes,  \(delta=0\)  but if different,  \(delta=1\) . 
 Here, each agent start with a weak bias oriented toward 0. Agents are not embedded in any network, but they will successively hear utterances “0”. We reported the delta at each time set for 500 agents. 
 
 
 
 Figure 48.  Figure showing the evolution of delta for 500 SAM and MAP agents. The thin curve is the aggregated result over the 500 agents, and the thick curve is the regression performed on these individuals (geom_smooth() using method = ‘loess’). Note that here, one time step = one utterance (“0”) heard.
 
 
 
 
 Appendix: Netlogo guide 
 
 BehaviorSpace 
 BehaviorSpace is a software tool integrated with NetLogo that allows you to perform experiments with models. BehaviorSpace runs a model many times, systematically varying the model’s settings and recording the results of each model run. This process lets you explore the model’s “space” of possible behaviors and determine which combinations of settings cause the behaviors of interest. After opening the Netlogo model, you can use BehaviorSpace in « Tools », « BehaviourSpace ». BehaviorSpace use is quite intuitive and will not be explained here. In the next part, I will explain the role of each variable in our Netlogo’s model. 
   
 
 
 Generating a network 
 Before generating a network, it is necessary to  setup-clear  the environment. 
 
 Num-agents. 
 Then, you can set the number of agents you want in this network ( num-agents ). In theory, you can select any number of agents > 2. In practice, data visualization will not be efficient for more than ~ 500 agents. In BehaviorSpace, and depending on the computer used, you can go up to 1000 agents without any computational problems, and 2000 agents if the number of conditions is not too high. 
  Note : if you want to select a very low number of agents (< 5), be careful to select the option «  On  » for choose-N-influent. Otherwise, if selected «  Off  », the program will try to give more 2 times more bias for central agents, but as it is impossible with 2 agents, it will keep looking for alternatives and eventually crash. 
 
 
 Directed/Undirected links. 
 In Other parameters, select «  directed  » or «  undirected  » in the box «  links-to-use  ». The default option is  undirected.  Please note that some additional options (centrality check, …) have been created only for  undirected  networks; if you want to use  directed  agents, there might be some problems (will require some more coding :) ) 
 
 
 Synchronicity. 
 Selecting the option “on” will make the network  synchroneous ; in the off mode, the network will be  asynchroneous.  More concretely, in a synchroneous network, the new language value of all nodes will be updated at the same time at the end of the tick, after all agents have talked once. In an asynchroneous network, the language value of the agents will be updated immediately after they hear the utterance. 
 
 
 Layout. 
 The layout affects the disposition of the agents for data visualization. It is useless to select this parameter in BehaviorSpace. You can select 4 layout options: «  spring  », «  circle  », «  radial  », «  tutte  ». 
 It is recommended to use the following parameters:  radial  for Scale-free, wheel and star network,  circle  for Random, small-world and ring networks.  Spring  layout can be a good alternative when plotting many types of networks. 
 The two buttons «  layout  » and «  layout once  » allow you to reset the layout once the network has been created. «  Layout  » will continuously reset the layout (useful for dynamic network) whereas «  layout once  » will reset the layout only once. 
 
 
 Dynamicity. 
 On the bottom left, there are several boxes under the note « Dynamic network parameters ». The default option is a static network, namely a network where  proba-rewire  = 0. 
  Proba-rewire  determines the probability to rewire, at each tick and for each agent. The total number of links remains the same throughout the process. The algorithm was built using the following algorithm: 
 
  for each agent, select a Random float R between 0 and 1: if R < proba-rewire, then start rewiring process for the selected agent i.  
  affect values for all agents in the network according to the selected agent  \(i\) : 
 
 the  distance  (=shortest path) between each agent and agent i. If no possible path exists, value 0 is affected. 
 the  similarity of the language value  between each agent and agent i. Here, similarity = absolute value of (language value of agent i - language value of other agents). 
 the  centrality  of agents (do not depend on agent i). Now, it uses betweenness centrality measure but this can be easily changed for other centrality measures. 
  
  normalize centralize these numbers so all measures vary between 0 and 1.  
  compute the overall probability of agent i to rewire with other agents. It uses the following formula: **(distance import-dist + similarity-language-value import-lang + centrality*import-central) / (import-dist + import-central + import-lang)** The values for import-dist, import-lang and import-central are manually selected by the user and represent weights that can be affected to each previously computed probability: for example, one can select higher importance of connecting with agents having a high centrality.  
  Then, it selects Randomly a agent  \(p\)  according to this probability, randomly remove one link of agent  \(i\) , and wire the agent  \(i\)  with agent  \(p\) .  
 
   
 In this example, agent  \(i\)  has a probability of 0.9 to rewire with agent  \(G\) , and a probability of 0.175 to rewire with agent  \(H\) . As agent  \(i\)  only has one neighbor (agent  \(F\) ), the link between agent  \(i\)  and agent  \(F\)  will be removed and we can imagine that a new link between agent  \(i\)  and  \(G\)  will be created. 
 Please note that dynamicity breaks the structure of scale-free network. Setting a high value for import-central will slow down this process but it will eventually look like a Random network after many ticks. 
 
 
 Network generation 
 Please note that in BehaviorSpace, whereas all variables (num-agents, etc) must be entered in the first box « vary variables », the network generation must be entered in setup commands. 
 So, in setup commands, you must enter first  setup-clear  and then the name of the network you want to generate ( Random  for example). You have several options : 
 
  Random  network: implies to select the  connection-prob  
  Small-world  network: according to watts-strogatz algorithm. Implies to select the  neighborhood-size  (the number of neighbors with whom each agent is connected at first) and the  rewire-prob  (the probability for each agent of rewiring after the initial condition has been set up). 
  preferential attachment : Scale-free network, according to Barabasi algorithm. 
  ring : each agent is connected to only one agent. Exactly the same as a scale-free with neighborhood-size=1 and rewire-prob = 0. 
  star : a central agent exists, relied with all agents. Other agents are only connected with this central agent. 
  wheel : same as star, except that not-central agents are also connected with their neighbors. 
 there are also other options such as  small-world lattice  (lattice-2d, kleinberg) algorithms, but this option is not perfectly working now (it does not fit to the number of agents previously entered but with the options nb-rows, nb-cols, and clustering-exponent). 
 
 More info at  here . 
 The network has been created! Please note that default values were used for the settings of the language value, bias, etc. 
 There are also buttons to  detect communities , to show  agents centralities , show  clusters  and  save matrix . 
 
 
 Affect initial language values 
 The internal value of the language is continuous and goes from 0 (against the feature) to 1 (pro the feature). Utterances are binary: with the feature (=1) and without the feature (=0). 
 
 Initial value of the society’s language 
 This part applies only to Bayesian communication algorithms. First of all, you need to select the initial value of the society’s language. Before it starts, each agent will hear a fixed number of utterances ( total-numb-utt ). If you do not want to have any initial value of the society language, affect 0 to  total-numb-utt . 
 Then, you need to select the number of utterance = 1 heard ( number-utt-heard-start ) among the N  total-numb-utt . For example, for non-biased agents, if they hear 3 utterances = 1 ( number-utt-heard-start  = 3) out of 4 utterances ( total-numb-utt ), these non-biased agents will start with an a-priori language tending toward possessing the feature. 
 
 
 Random initial value language 
 The agents can start with a language value which is determined by the bias ( Random-initial-lang-value = Off) , or with chosen Random initial values for the language ( Random-initial-lang-value = On ). 
 For Bayesian algorithm, we recommend to start with language value determined by the bias. For non-Bayesian communication algorithm, such as probabilistic algorithm, setting this value to «  on  » allows settling the initial value of the society language. Now, it is only possible to affect values according to a normal distribution but it can easily be changed for other types of distributions. You can set  initial-value-language  and the  standard-dev  of the normal distribution; if you want all agents to have the same value, set standard-dev = 0. 
 For non Bayesian communication algorithms, it is also possible to set the values for a unbiased value of the feature. We recommend affecting the same value to the unbiased. 
 
 
 Redistribute/reset buttons 
 If you want to re-use exactly the same language-values as the one you have just used, use  reset language value . 
 If you want to redistribute language values in the same network (give new language values), use the button  redistribute language values . 
 
 
 
 Affect internal bias 
 First of all, you need to select if you want  binary  or  continuous  bias. If you want to split the population into 2 parts: agents biased with a X bias and agents biased with a Y bias (could be non-biased), use  binary bias . If you want the population to be continuously biased on a range from 0 to 1, use  continuous bias . 
 For now, with Bayesian communication algorithms, the program only handles  binary bias . 
 
 Continuous bias (only for non-Bayesian) 
 You have two options: either set Random continuous states ( is-state-Random = On ) or manually choose the distribution of the continuous states ( is-state-Random = Off ). 
 If you set this option to  On , there is no need to fill the other boxes below. 
 If you set this option to  Off , you select the shape of the distribution for the states  (distribution-state ). Then, according to the distribution chosen, you can select the parameters of the curve. For example, if you selected a gamma distribution, you only need to select the options «  alpha-if-gamma  » and «  lambda-if-gamma  ». 
 The last option ( exactly-same-for-unbiased ) will affect the same values for the unbiased bias. Now, the option for which you can manually select the values for the unbiased bias has not been coded yet. Please note that you can visualize the distribution of the state in the graph below. 
 
 
 Binary bias (for all) 
 Use this if you want to split the population into 2 parts. 
 First, affect the value of the bias for population 1 ( init-langval-1 ) and the value of the bias for population 0 ( init-langval-0 ). Please note that if you want to have a biased population and a non-biased population, the biased population must always be the population 1. Indeed, the centrality measures are computed with this population. 
 Second, select the percentage of agents in population 1 ( percent-state-1 ). There is also a unbiased bias for which you can select an other percentage unbiased. The bias unbiased has not been coded for Bayesian algorithms. 
 Third, select how you want the bias to be distributed according to the agents’ centrality. There are two options: select the bias according to the most influential agents ( choose-N-influent = On ), or select the bias according to the ratio of centrality ( choose-N-influent=Off ). 
  choose-N-influent = On : You can use this option with any type of network. If you select this option, you must enter the number of the most influent agent that will have the bias  init-langval-1 . This number must always be inferior to number of agents in population 1 (percent-state-1 * num-agents): in Netlogo, the cursor will automatically adapt but in BehaviorSpace, make sure not to enter a too high number. The algorithm works this way: 
 
 affect init-langval-0 to all agents 
 find most influential agents (now, it uses eigenvector centrality but it can be easily changed in the code) 
 affect init-langval-1 to the  N-influent  most influential agents selected by the user. For example, if this  N-influent=2 , the 2 most influential agents in the network will have init-langval-1. 
 affect randomly  init-langval-1  to the rest of the agents, so that the number of agents with  init-langval-1  fits the  percent-state1 . 
 
  choose-N-influent = Off:  You can select this option only with Scale-free networks. If you select this option, you must enter the  ratio-centrality-Scale-free  that you want to see. This ratio means that the centrality of the agents with  init-langval-1  must be X times higher than the mean centrality of the agents with  init-langval-0 . The algorithm works this way: 
 
 affect  init-langval-0  to all agents 
 « while » loop :
 
 randomly affect  init-langval-1  in the network; 
 compute the mean centrality for agents with  init-langval-1  and  init-langval-0 ; 
 if the ratio mean centrality of population 1 agents = ratio-centrality-Scale-free * mean centrality of population 0 agents +/- 0.01 (we added 0.01 in order to have a loop which runs faster…), then break the loop. If not, redo everything. 
  
 
 The while loop works relatively fast thanks to the addition/subtraction of 0.01. For data visualization, it is easy to test a high ratio, such as 5 or 6. However, in BehaviorSpace, as we run many iterations, affecting a too high value for the ratio can make the program crash. We recommend to use ratio-centrality = 1 or 2. 
 If you don’t want to make this centrality parameter vary, you have 2 options: 
 
 set  choose-N-influent = On  and  N-influent = 0 : in this case,  init-langval-1  will be affected randomly in the population; 
 set  choose-N-influent = Off  and  mean-centrality = 1 : this is different than the previous case, in that the mean centrality of agents with  init-langval-1  and with  init-langval-0  will be approximately the same. For many replications, the 2 options are equivalent but the first option is recommended, because computationally less expensive. 
 
 Same as for language-value, you can  reset  or  redistribute states . Please note that you can visualize the mean centrality of agents with state = 1 and state = 0. 
 
 
 
 Choose communication algorithm 
 First, you need to select the communication algorithm. You have several options: 
 
 Individual algorithm 
 Each tick, affect to the agent the language value of one randomly selected neighbor. 
 (borrowed from Language Change model library) 
 
 
 Threshold algorithm 
 (works with  threshold-val  and  sink-state-1  options): 
 
 sum the language value of all neighbors 
 if this sum is > the number of neighbors * threshold-val, then affect language-value to 1. 
 if you affected  sink-state-1 to Off , and this sum is < the number of neigbors * threshold val, then affect language-value to 0. 
 
 (borrowed from Language Change model library) 
 
 
 Reward algorithm 
 (works with  value-bias  and  logistic ): 
 
  affect to agents either a value-bias = 0 for population 0, or = value-bias (manually selected by the experimenter) for population 1.  
  Create utterances according to the internal value-bias: 
 
 if logistic: create a value: (1 / (1 + exp ( - ((value-bias+0.1) 20 language-value – 1) * 5)) → basically, it means that if you are non biased (value-bias = 0), you produce utterance according to the internal value of your language, but if you’re biased, you tend to product more utterance = 1. 
 if not logistic: agents produce utterance only according to their internal language value (no bias toward the feature) 
  
  Listen to utterances spoken by neighbors: change your internal language value according to the neighbors’ utterances.  
 
 (borrowed from Language Change model library) 
 
 
 Probabilistic algorithm 
 (works with  value-bias-for-state1  and  value-bias-for-state-0  for binary option, and  min-value-bias  and  max-value-bias  for continuous option) 
 
   Speak : extract 30 utterances according to binomial function with the language-value as the probability. The spoken-state is the mean of the 30 utterances. Contrary to reward algorithm, speaking is not affected by the bias.  
   Listen  using the following formula:  
 
 ifelse heard-state >= language-value 
 new-lang-value = ( language-value + ( abs(language-value - heard-state) * value-bias-for-state-0 ) ) 
 new-lang-value = ( language-value - ( abs(language-value - heard-state) * (value-bias-for-state-0 for non-biased agents; value-bias-for-state-1 for biased agents) ) ) 
  
 
 Consequently, biased agents have a tendency to decrease less their internal value of language when they hear utterance lower than their internal value of the language compared to non-biased agents. 
 
 
 Bayesian algorithm 
 (works with  learning-acceptance1 ,  learning-acceptance0 ,  init-langval-0  and  init-langval-1 ) 
 
  The parameters  learning-acceptance  and  init-langval-1  are used in another R script to generate alpha and beta values for a Beta distribution. The code works in Netlogo; but because it was often crashing with BehaviorSpace, I just extracted the alpha and beta value of interests and copy-pasted in Netlogo. According to those values, each agent is initialized with a Beta distribution than will be modified according to the utterances heard.  
   Speak : two options can be set: 
 
  MAP : - extract the value of the mode of the Beta distribution; - use the binomial function to extract an utterance from this probability 
  SAM : - extract a Random number from the Beta distribution; - use the binomial function to extract an utterance from this probability 
  
   Listen : the same for both MAP and SAM algorithms  
 
 Update the Beta distribution using the following formula: - new alpha = alpha + utterance heard (= 0 or 1), - new beta = beta + 1 – utterance heard (= 0 or 1) 
  
 
 
 
 
 
 
 References 
 
 
 Albert, R. (2005). Scale-free networks in cell biology.  Journal of Cell Science ,  118 (21), 4947–4957.  https://doi.org/10.1242/jcs.02714  
 
 
 Albert, R., Jeong, H., & Barabási, A.-L. (1999). Diameter of the World-Wide Web.  Nature ,  401 (6749), 130–131.  https://doi.org/10.1038/43601  
 
 
 Barabási, A.-L., Albert, R., & Jeong, H. (2000). Scale-free characteristics of random networks: The topology of the world-wide web.  Physica A: Statistical Mechanics and Its Applications ,  281 (1), 69–77.  https://doi.org/10.1016/S0378-4371(00)00018-2  
 
 
 Dediu, D. (2008). The role of genetic biases in shaping language-genes correlations.  Journal of Theoretical Biology ,  254 , 400–407.  https://doi.org/doi:10.1016/j.jtbi.2008.05.028  
 
 
 Dediu, D. (2009). Genetic biasing through cultural transmission: Do simple Bayesian models of language evolution generalize?  Journal of Theoretical Biology ,  259 (3), 552–561.  https://doi.org/10.1016/j.jtbi.2009.04.004  
 
 
 Erdős, P., & Rényi, A. (1959). On Random Graphs I.  Publicationes Mathematicae (Debrecen) ,  6 , 290–297. 
 
 
 Griffiths, T. L., & Kalish, M. L. (2007). Language evolution by iterated learning with Bayesian agents.  Cognitive Science ,  31 (3), 441–480. Retrieved from  http://onlinelibrary.wiley.com/doi/10.1080/15326900701326576/full  
 
 
 Jannsen, R. (2018).  Let the agent do the talking: On the influence of vocal tract anatomy on speech during ontogeny and glossogeny . Nijmegen. 
 
 
 Kenett, Y. N., Levy, O., Kenett, D. Y., Stanley, H. E., Faust, M., & Havlin, S. (2018). Flexibility of thought in high creative individuals represented by percolation analysis.  Proceedings of the National Academy of Sciences ,  115 (5), 867–872.  https://doi.org/10.1073/pnas.1717362115  
 
 
 Kirby, S., Dowman, M., & Griffiths, T. L. (2007). Innateness and culture in the evolution of language.  Proc Natl Acad Sci U S A ,  104 (12), 5241–5245.  https://doi.org/10.1073/pnas.0608222104  
 
 
 Kitsak, M., Gallos, L. K., Havlin, S., Liljeros, F., Muchnik, L., Stanley, H. E., & Makse, H. A. (2010). Identification of influential spreaders in complex networks.  Nature Physics ,  6 (11), 888–893.  https://doi.org/10.1038/nphys1746  
 
 
 Milgram, S. (1967). The small-world problem.  Psychology Today ,  1 (1), 61–67. Retrieved from  http://files.diario-de-bordo-redes-conecti.webnode.com/200000013-211982212c/AN%20EXPERIMENTAL%20STUDY%20by%20Travers%20and%20Milgram.pdf  
 
 
 Watts, D. J., & Strogatz, S. H. (1998). Collective dynamics of “small-world” networks.  Nature ,  393 (6684), 440–442.  https://doi.org/10.1038/30918  
 
 
 


 
 

 

 

 

 

 

 

 
 

 
 
